# A specific circuit in the midbrain detects stress and induces restorative sleep

**DOI:** 10.1126/science.abn0853

**Published:** 2022-06-30

**Authors:** Xiao Yu, Guangchao Zhao, Dan Wang, Sa Wang, Rui Li, Ao Li, Huan Wang, Mathieu Nollet, You Young Chun, Tianyuan Zhao, Raquel Yustos, Huiming Li, Jianshuai Zhao, Jiannan Li, Min Cai, Alexei L. Vyssotski, Yulong Li, Hailong Dong, Nicholas P. Franks, William Wisden

**Affiliations:** a Department of Life Sciences, Imperial College, London SW7 2AZ, United Kingdom; b UK Dementia Research Institute, Imperial College, London SW7 2AZ, United Kingdom; c Department of Anesthesiology & Perioperative Medicine, Xijing Hospital, Fourth Military Medical University, Xi’an, China; d Department of Psychiatry, Xijing Hospital, Fourth Military Medical University, Xi’an, China; f UK Dementia Research Institute, King’s College, London SE5 9RT, United Kingdom; g Institute of Neuroinformatics, University of Zürich/ETH Zürich, Zürich, Switzerland; h State Key Laboratory of Membrane Biology, Peking University School of Life Sciences, Beijing, 100871, China; i PKU-IDG/McGovern Institute for Brain Research, Beijing, 100871, China

## Abstract

In mice, social defeat stress (SDS), an ethological model for psychosocial stress, induces sleep. Such sleep could enable resilience, but how stress promotes sleep is unclear. Activity-dependent tagging revealed a subset of ventral tegmental area GABA-somatostatin (VTA^*Vgat-Sst*^) cells that sense stress and drive NREM and REM sleep via the lateral hypothalamus, and also inhibit corticotropin-releasing factor (CRF) release in the paraventricular hypothalamus. Transient stress enhances the activity of VTA*^Vgat-Sst^* cells for several hours, allowing them to exert their sleep effects persistently. Lesioning of VTA*^Vgat-Sst^* cells abolished SDS-induced sleep; without it, anxiety and corticosterone levels remained elevated after stress. Thus, a specific circuit allows animals to restore mental and body functions via sleeping, potentially providing a refined route for treating anxiety disorders.

## Introduction

Acute stress activates the hypothalamic-pituitary-adrenal axis, and the resulting fast increase in blood glucocorticoid levels aids immediate survival (1–3). But chronically elevated levels of glucocorticoids are harmful (1, 2), as can be memories of stressful experiences (4). Although stress can cause insomnia and raise stress hormones (3, 5–8), the opposite is also true: chronic stress elevates REM sleep (9); and sleep in rodents is induced by specific types of stress, such as social defeat stress (SDS). Although the function and benefits of sleep remain unclear, sleep is certainly restorative (10). Thus, sleep has been suggested to be one of the mechanisms for alleviating the malign effects of stress (4, 9, 11, 12). Whether a specific circuit links stress and sleep is, however, unknown. We reasoned that the ventral tegmental area (VTA) in the midbrain could provide a link.

The VTA regulates reward, aversion, goal-directed behaviors and social contact (13–15). It also influences responses to stress and threats (16, 17), and strongly affects sleep and wake: VTA*^Vglut2^* and VTA^*TH*^ neurons promote wakefulness (18, 19); whereas VTA*^Vgat or Gad67^* neurons induce sleep (18, 20, 21). Because some GABAergic VTA neurons are activated by stressful and aversive stimuli (16, 22–24), we hypothesized that this route allows stress to induce sleep.

## Social defeat stress induces sleep

**Fig. 1:**
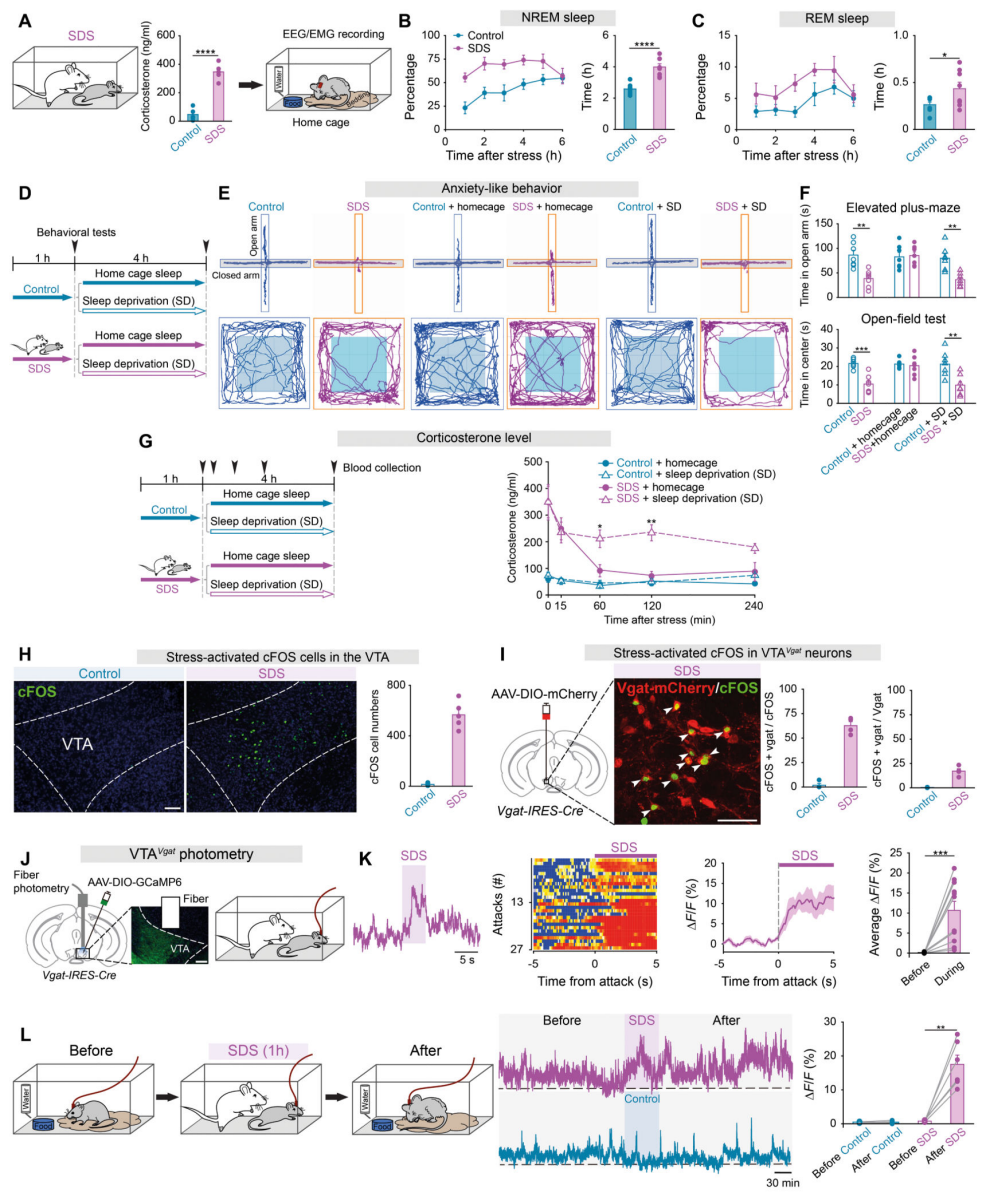
Stress increases sleep while sleep reduces SDS-induced anxiety and stress activates VTA*^Vgat^* neurons. (**A**) The experimental procedure and corticosterone levels (n=6 mice per group). (**B, C**) Percentage and time of NREM (**B**) and REM (**C**) sleep after control or SDS (n=8 mice per group). (**D-F**) Plan of the experimental procedure (**D**), tracing of locomotion for representative animals (**E**), time spent in the open arms of the elevated plus-maze and in the center zone during the open-field test (**F**) (n=7 mice per group). (**G**) Plan of the experimental procedure and corticosterone levels (n=6 mice per group). (**H, I**) cFOS expression and quantification in the VTA after control or SDS (n=5 mice per group) (**H**); or in genetically labeled VTA*^Vgat^* neurons (n=4 mice per group) (**I**). Arrowheads indicate double-labeled cells. Scale bar, 100 μm. (**J, K**) Fiber photometry setup and GCaMP6 expression in VTA*^Vgat^* neurons (**J**). Fiber photometry measuring calcium signals responding to SDS (n=11 mice, 27 trials). Raw calcium signal traces, color matrix of signals for all trials, ΔF/F ratios across the experimental period and average ΔF/F ratios before and during the procedure (**K**). Scale bar, 100 μm. (**L**) Fiber photometry measuring long-term calcium signals in VTA*^Vgat^* neurons. Traces across the experimental procedure and average ΔF/F ratios before and after the procedures (n=6 mice per group). (**A, B, C**) Unpaired *t*-test, *p<0.05, ****p<0.0001; (**F, G**) Two-way ANOVA with bonferroni *post hoc* test, *p<0.05, **p<0.01, ***p<0.001; (**K, L**) Paired *t*-test, **p<0.01, ***p<0.001.

We first assessed the sleep-wake architecture of mice after they had experienced either SDS from aggressors with consecutive episodes of SDS for one hour, or a control procedure, in which the experimental mouse (intruder) was separated from the resident aggressor by a clear partition (Fig. S1A). As a further control, instead of an aggressor mouse, we introduced a juvenile male mouse as the resident for 1 hour (Fig. S1B). During this non-stress procedure, the experimental mice experienced social interaction. As a control for whether physical activity induces sleep, the mice experienced voluntary wheel running or forced treadmill running continuously for 1 hour (Fig. S1C, D); or, alternatively, as another control, the mice were placed in a novel environment with a novel object (Fig. S1E). Corticosterone (CORT) levels in mice increased after the SDS sessions (Fig. 1A), but not following exposures to juvenile mice, physical exercise, or novel environment/objects (Fig. S2A, C, E, G). After SDS, NREM sleep latency was shortened, and both NREM and REM sleep were continuously elevated for 5 hours (Fig. 1B, C and Fig. S2I), consistent with previous observations (11, 25). Mice that experienced a non-stressful procedure, voluntary wheel running, forced treadmill running or that were deprived of one hour of sleep by placing them in a novel environment, however, did not have induced sleep above baseline (Fig. S2A-H, J-M), suggesting social interaction or physical exercise did not induce sleep and SDS procedures did not cause a sleep rebound while the mice were awake.

## Sleep relieves SDS-induced anxiety and CORT levels independently

We explored potential functions of sleep after SDS. For mice allowed sufficient sleep (home cage sleep) after SDS, anxiety-like behaviors caused by SDS were rapidly reduced, as seen in the elevated-plus maze and open-field assays (Fig. 1D-F). If mild sleep deprivation over 4 hours took place immediately after SDS, the mice remained in an anxious state (Fig. 1D-F). For mice allowed sufficient home cage sleep after SDS, raised CORT levels returned to baseline over 60 mins (Fig. 1G). If mild sleep deprivation occurred immediately after stress, however, CORT levels remained elevated (Fig. 1G). But pharmacologically reducing the CORT levels induced by SDS during sleep deprivation, using a corticosterone synthesis inhibitor metyrapone (Fig. S3A), did not reduce anxiety after sleep deprivation (Fig. S3B, C).

## Identification of neurons activated by stress

To identify the circuitry that induces restorative sleep, we mapped cFOS expression throughout the brain. Following the SDS protocol (Fig. S1A), cFOS was elevated strongly in brain areas involved in stress responses (Fig. S4A, B), including the VTA (Fig. 1H and Fig. S4). In the VTA, cells activated (cFOS-positive) by stress predominantly expressed the GABAergic marker *Vgat* (60%) or GABA (57%) (Fig. 1I and Fig. S5A, B), whereas relatively fewer cells expressed the glutamatergic marker *Vglut2* (20%) or the dopaminergic marker tyrosine hydroxylase (10%) (Fig. S5C, D). However, physical exercise did not induce cFOS in the VTA as a whole, and particularly not in VTA*^Vgat^* cells, but forced treadmill running slightly increased cFOS expression in TH-positive cells (Fig. S5E, F, G). For the subsequent studies, we focused on the VTA*^Vgat^* neurons, as only these induce sleep (18).

## VTA*^Vgat^* neurons have persistently increased activity in response to SDS

VTA*^Vgat^* neurons rapidly and strongly responded when mice experienced an attack during SDS (Fig. 1J, K), as assessed by GCaMP6 fiber photometry. The cells did not respond when the mice were presented with novel objects or placed in a novel environment (Fig. S6A, B). During SDS, the calcium signal in VTA*^Vgat^* neurons increased, and stayed enhanced for about 5 hours (Fig. 1L), correlating with the behavioral result of prolonged sleep after SDS (Fig.1B, C). In contrast, voluntary wheel running, forced treadmill running or a novel environment did not affect baseline activity of VTA*^Vgat^* neurons (Fig. S6C, D).

## Subsets of VTA*^Vgat^* neurons mediate SDS-induced sleep

**Fig. 2:**
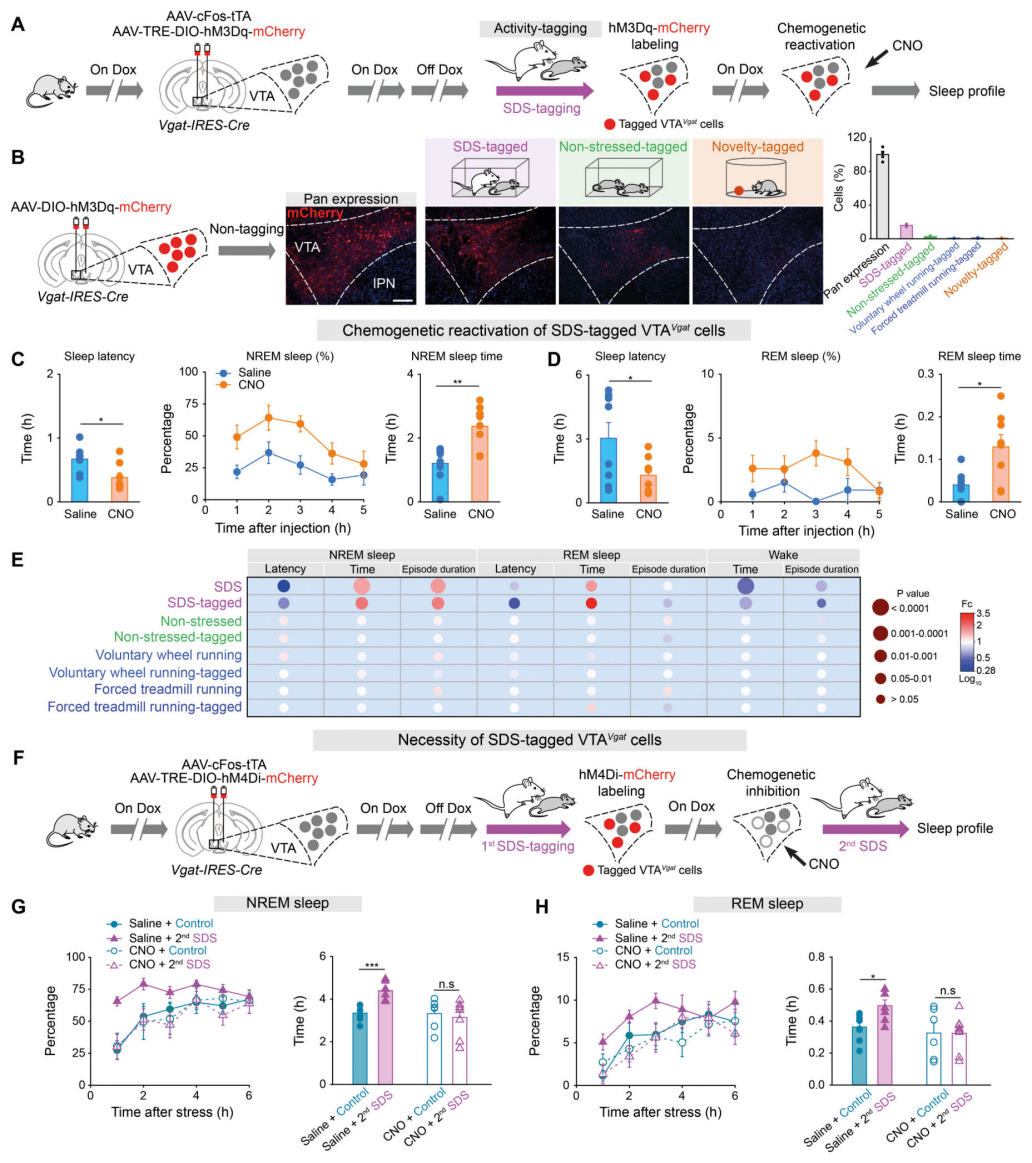
Sufficiency and necessity of the stress-activated VTA*^Vgat^* neurons for SDS-induced sleep (**A**) The activity-tagging protocol for testing the sufficiency of SDS-activated VTA*^Vgat^* cells for sleep. (**B**) Expression and quantification of pan or activity-tagged hM3Dq-mCherry transgene in VTA*^Vgat^* neurons (n=4 mice per group). Scale bar, 100 ^m. (**C, D**) Chemogenetic reactivation of tagged VTA*^Vgat^* neurons for sleep (n=8 mice per group). Graphs show sleep latency, percentage and time of NREM **(C)** or REM (**D**) sleep. Unpaired *t*-test, *p<0.05, **p<0.01. (**E**) Matrix bubble summary shows fold of changes (Fc) of sleep parameters after SDS, non-stressed, voluntary wheel running, forced treadmill running or the chemogenetic reactivation of tagged VTA*^Vgat^* neurons. (**F**) Activity-tagging protocol for testing the necessity of SDS-activated VTA*^Vgat^* cells for sleep. (**G, H**) Percentage and time of NREM (**G**) or REM sleep (**H**) in mice given 2^nd^ SDS after chemogenetic inhibition of 1^st^ SDS-tagged VTA*^Vgat^* cells (n=6-8 mice per group). Two-way ANOVA with bonferroni *post hoc* test. *p<0.05, ***p<0.001, n.s: not significant.

Because only a subset of VTA*^Vgat^* neurons (20%) were excited by SDS (Fig. 1I), we undertook cFOS-dependent activity-tagging linked to expression of DREADD hM3Dq-mCherry to test if this VTA subset could induce sleep (Fig. 2A). Mice experienced either SDS, or a non-stressful procedure, voluntary wheel running or forced treadmill running, while the VTA*^Vgat^* neurons were selectively activity-tagged using Cre-recombinase-dependent tagging vectors (*AAV-cFOS-tTA* and *AAV-TRE-DIO-hM3Dq-mCherry,* Fig. 2A and Fig. S7A, D, G). Compared with pan-VTA*^Vgat^* neurons expressing mCherry, only 15% of the VTA*^Vgat^* neurons were captured by activity-tagging during SDS (Fig. 2B). We then reactivated these SDS-tagged VTA*^Vgat^* neurons with CNO. Chemogenetic reactivation decreased sleep latencies, and increased sleep times (Fig. 2C, D). Thus, reactivation of SDS-activated VTA*^Vgat^* neurons recapitulated sleep architectures induced by SDS (Fig. 2E). Of note, a few cells (2.6%) were tagged during the non-stressed procedures (Fig. 2B). However, chemogenetic reactivation of these particular tagged VTA*^Vgat^* cells did not elicit sleep (Fig. S7B, C). Moreover, only rare cells were tagged when mice experienced physical exercise (Fig. 2B), and therefore chemogenetic reactivation did not induce sleep (Fig. S7D-I).

To examine the necessity of VTA*^Vgat^* subsets for SDS-induced sleep, we chemogenetically inhibited SDS-tagged VTA*^Vgat^* neurons using cFOS-dependent expression of DREADD hM4Di-mCherry. *AAV-cFOS-tTA* and *AAV-TRE-DIO-hM4Di-mCherry* were injected into the VTA of *Vgat-IRES-Cre* mice (Fig. 2F). Mice were subjected to SDS (1^st^ stress episode) to allow VTA*^Vgat^* neurons to become tagged with hM4Di-mCherry, then given CNO to inhibit the tagged neurons, and mice were subsequently challenged with a second bout of SDS (2^nd^ stress episode), followed by measurement of their sleep profile (Fig. 2F). SDS-induced sleep was diminished after chemogentically-inhibiting tagged VTA*^Vgat^* neurons (Fig. 2G, H).

## Circuits linking stress and sleep

**Fig. 3:**
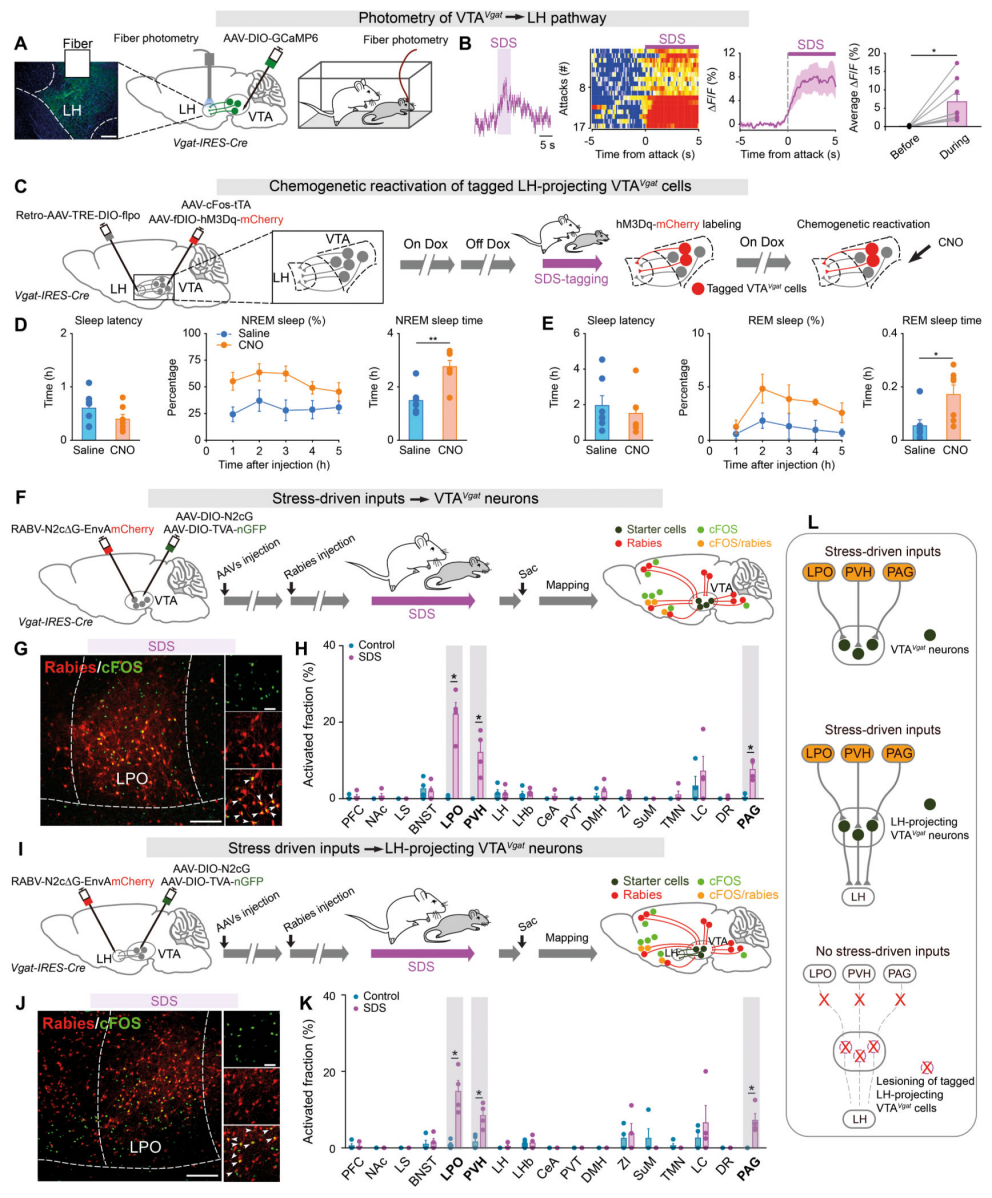
Input-output circuitry linking stress and sleep (**A, B**) Fiber photometry measuring terminal calcium signals of the VTA*^Vgat^*→LH pathway responding to SDS (**A**). Raw traces, color matrix of GCaMP6 signals of VTA*^Vgat^*→LH for all trials, ΔF/F ratios across the experimental period and average ΔF/F before and during SDS (n=8 mice, 17 trials) (**B**). Paired *t*-test, *p<0.05. Scale bar, 100 μm. (**C**) Activity-tagging protocol for reactivating the SDS-tagged LH-projecting VTA*^Vgat^* cells. (**D, E**) Sleep latency, percentage and time of NREM (**D**) and REM (**E**) sleep after reactivation of SDS-tagged LH-projecting VTA*^Vgat^* cells (n=7 mice per group). Unpaired *t*-test, *p<0.05, **p<0.01. (**F**) Rabies virus-based retrograde tracing for identification of stress-driven inputs to VTA^*Vgat*^ neurons. (**G**) Immunostaining images showing presynaptic inputs to VTA*^Vgat^* neurons from LPO, and cFOS-positive cells activated by SDS. Scale bar, 200 μm and 50 μm (inset). (**H**) Summary statistics of activated fractions (cFOS/rabies double-labeled cells/total rabies-positive cells) (n=4 mice per group). For abbreviations, see Fig. S4. Mann-Whitney test, *p<0.05. (**I**) Protocol for identification of stress-driven inputs to VTA*^Vgat^* neurons that output to LH. (**J**) Immunostaining shows presynaptic inputs to LH-projecting VTA*^Vgat^* neurons from LPO, and cFOS-positive cells activated by stress. Scale bar, 200 μm and 50 μm (inset). (**K**) Summary statistics of activated fractions to LH-projecting VTA*^Vgat^* neurons (n=4 mice per group). Mann-Whitney test, *p<0.05. (**L**) Schematic diagram summarizing the stress-driven input-output relations.

We next investigated the circuitry linking SDS and VTA*^Vgat^*-induced sleep. We expressed GCaMP6 selectively in VTA*^Vgat^* cells and used fiber photometry to measure how the VTA^*Vgat*^ terminals in different locations responded to stress. Only the terminals of the VTA*^Vgat^* cells projecting to the LH had increased Ca^2+^ signals following SDS (Fig. 3A, B), whereas the VTA*^Vgat^* projections in the CeA, LHb, and hippocampal dentate granule cells (DG) showed no responses (Fig. S8). To determine the function of the VTA*^Vgat^*→LH pathway activated by stress on sleep, we injected *retro-AAV-TRE-DIO-Flpo* into the LH, together with the injection of *AAV-cFOS-tTA* and *AAV-fDIO-hM3Dq-mCherry* into the VTA of *Vgat-IRES-Cre* mice (Fig. 3C). Following intersectional activity-tagging during SDS and chemogenetic reactivation, VTA→LH pathway promoted sleep (Fig. 3D, E). The hM3Dq-mCherry labeling produced in the VTA*^Vgat^* neurons of this experiment mainly traced out axons to the LH (Fig. S9).

We used optogenetics to confirm the above result. The behavioral experiments were repeated using cFOS-based activity-tagging with ChR2 delivered into the VTA of *Vgat-IRES-Cre* mice (Fig. S10A). VTA*^Vgat^* neurons became selectively ChR2-tagged during SDS (Fig. S10A). Mapping of VTA*^Vgat^* projections by injecting *AAV-DIO-ChR2-EYFP* into *Vgat-IRES-Cre* mice showed broad projections (Fig. S10B) (18). However, those SDS-tagged VTA*^Vgat^* cells detected with ChR2 activity-mapping primarily innervated the LH (Fig. S10C). When SDS-ChR2-tagged terminals in the LH of the VTA*^Vgat^*→LH pathway were reactivated by optogenetic-stimulation, this elicited NREM sleep from waking (Fig. S10D, E).

## Stress-driven input-output organizations

We investigated the identity and activity of VTA*^Vgat^* afferents relevant for stress using a rabies system, combined with activity mapping. VTA*^Vgat^* neurons were seeded with rabies coat protein and its receptor by injecting *AAV-DIO-N2cG* and *AAV-DIO-TVA-nGFP*, followed by injection of *RABV-N2cΔG-EnvAmCherry* into the VTA (Fig. 3F). The animals were then given control experiences or SDS, respectively. Then we conducted brain-wide mapping of rabies-labeled presynaptic inputs and stress-activated cFOS expression (Fig. 3F). cFOS was induced by stress in many brain regions (Fig. S4), and from the rabies tracing, VTA*^Vgat^* inputs originated in many locations (Fig. S11) (26). However, only the LPO, PVH and PAG areas had overlap with cFOS-positive cells and rabies-labeled VTA*^Vgat^* inputs (Fig. 3G, H, L and Fig. S12A-C, S13A). We determined the inputs of VTA*^Vgat^* neurons that project to the LH. *AVV-DIO-N2cG* and *AAV-DIO-TVA-nGFP* were seeded as before in VTA*^Vgat^* neurons, and *RABV-N2cΔG-EnvAmCherry* was injected into the terminal fields of the VTA*^Vgat^* neurons in the LH. As before, the mice were given control experiences or SDS. Then we mapped cFOS expression (Fig. 3I). We obtained an identical result as above: LH-projecting VTA*^Vgat^* neurons received stress-activated inputs from LPO, PVH and PAG (Fig. 3J, K, L and Fig. S12D-F, S13B).

We further determined if these stress-activated inputs were specific to the stress-activated VTA*^Vgat^* subset. The SDS-activated VTA*^Vgat^* cells were specifically ablated with Casp3 using activity-tagging *(AAV-cFOS-tTA* and *AAV-TRE-DIO-Casp3).* Then we conducted rabies tracing and activity mapping (Fig. S14A). Ablation of SDS-activated VTA*^Vgat^* cells largely reduced the stress-driven inputs (cFOS/rabies) (Fig. S14B, C and Fig. 3L).

## VTA somatostatin neurons are necessary for SDS-induced sleep

Given that GABAergic VTA cells are heterogeneous (13, 27, 28), and only a subset of VTA^*Vgat*^ cells responded to SDS (Fig. 2B), we looked for subtypes of VTA^*GABA*^ cells responsible for SDS-induced sleep. First, we examined by single-cell qPCR the molecular identities of SDS-tagged cells (Fig. S15A): a large proportion (42%) expressed *vgat*/somatostatin (*sst*), and others were characterized by *vgat*/parvalbumin *(pv)* (10%) or *vgat expression* alone (32%), the remaining cells being 2% *vgat/vglut2,* 2% *vgat/vglut2/sst* and 2% *vgat/vip* (Fig. S15B).

**Fig. 4:**
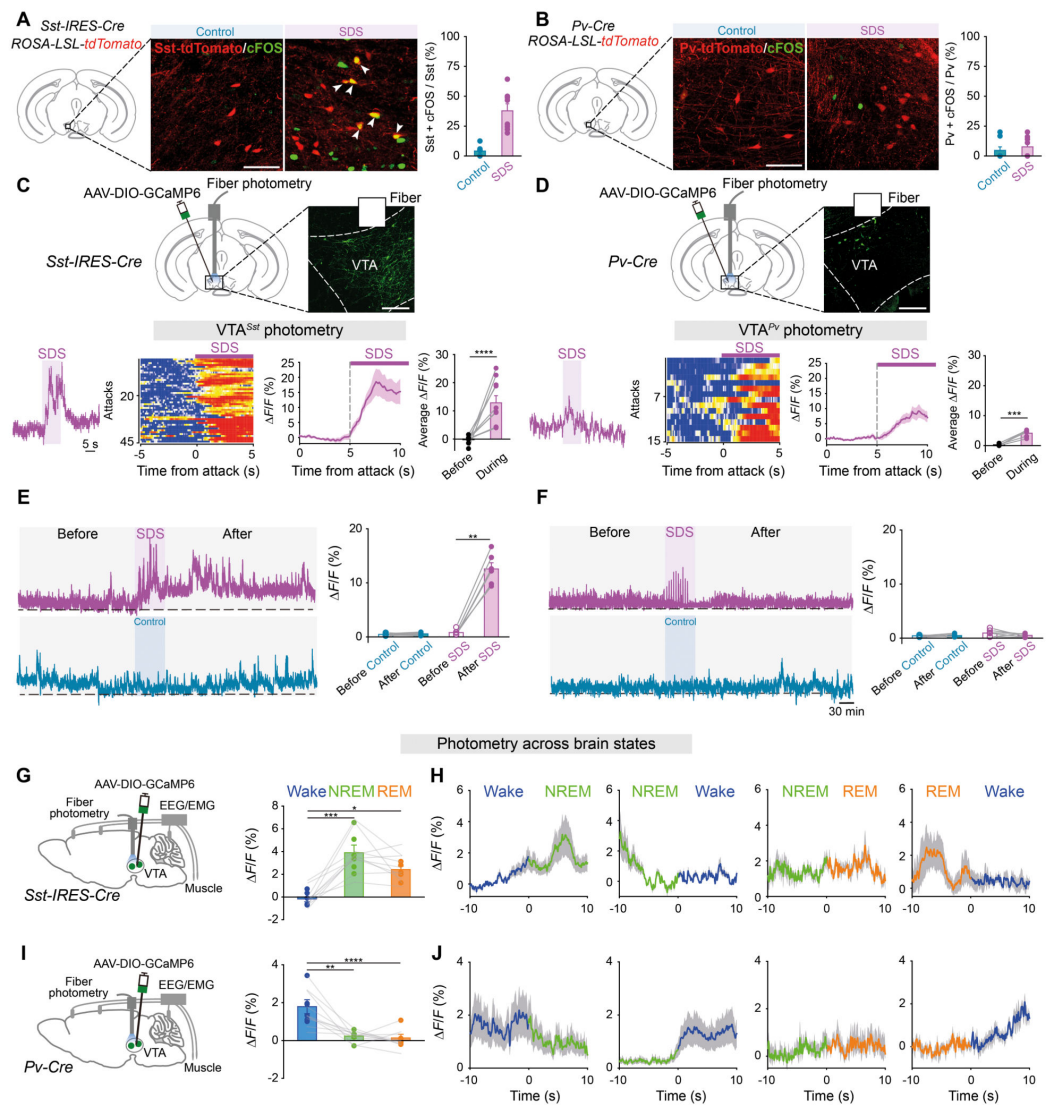
Activity of VTA^*Sst or Pv*^ neurons responding to stress and across brain states (**A, B**) cFOS expression and quantification in genetically labeled VTA*^Sst^* **(A)** or VTA*^Pv^* **(B)** neurons after control experience or SDS (n=8 mice per group). Scale bar, 100 μm. (**C, D**) Fiber photometry measuring calcium signals in VTA*^Sst^* (n=10 mice per group, 45 trials) (**C**) or VTA*^Pv^* (n=6 mice per group, 15 trials) (**D**) neurons responding to SDS. Raw calcium signal traces, color matrix of signals for all trials, ΔF/F ratios across the experimental period and average ΔF/F ratios before and during SDS. Paired *t*-test, ***p<0.001, ****p<0.0001. Scale bar, 200 μm. (**E, F**) Fiber photometry measuring long-term calcium signals in VTA*^Sst^* **(E)** or VTA*^Pv^* **(F)** neurons. Raw traces and average ΔF/F ratios before and after the procedures (n=6 mice per group). Paired *t*-test, **p<0.01. (**G-J**) Fiber photometry with EEG/EMG measuring spontaneous activity across brain states. ΔF/F ratios in VTA*^Sst^* (**G**) or VTA*^Pv^* (**I**) neurons during wakefulness, NREM and REM sleep, and at transitions of vigilance states (n=6 mice per group) (**H, J**). One-way Repeated ANOVA, *p<0.05, **p<0.01, ***p<0.001, ****p<0.0001.

We further characterized the SDS-activated cells using reporter mice (Fig. 4A, B and Fig. S15C, D). Nearly 40% of the VTA*^Sst^* neurons expressed cFOS following SDS (Fig. 4A and Fig. S15E, F), whereas there was no induction of cFOS following SDS in VTA*^Pv^* cells (Fig. 4B).

We next determined the activity of individual subtypes responding to stress using fiber photometry (Fig. 4C, D). Both the VTA*^Sst^* and VTA*^Pv^* populations responded transiently to SDS, but the collective calcium signal for VTA*^Sst^* cells was larger (Fig. 4C, D), and only VTA^*Sst*^ neurons had persistent activation following SDS, with enhanced activity for a few hours (Fig. 4E). In contrast, the transient activity of VTA*^Pv^* neurons after SDS was not sustained (Fig. 4F). We next used activity-tagging with hM3Dq to capture SDS-tagged VTA*^Sst^* neurons (Fig. S16A, B). Because VTA*^Sst^* cells are heterogeneous (28), we examined the molecular identities of SDS-tagged cells (Fig. S16C). These tagged cells predominantly expressed *vgat/gadl* (90%) (Fig. S16D).

We tested whether VTA*^Sst^* cells could respond to two types of insomnia-inducing stress, restraint and cage-change (7, 29). However, these procedures did not affect the acute or long-term calcium activity in VTA*^Sst^* neurons (Fig. S17A-D). In addition, we did not observe any VTA*^Sst^* neurons becoming tagged by restraint stress or cage-change stress (Fig. S17E).

Next, we measured spontaneous activities of VTA*^Sst^* neurons across brain states. From calcium photometry, VTA*^Sst^* neurons were primarily active during spontaneous NREM and REM sleep (Fig. 4G, H), whereas VTA*^Pv^* neurons were wake-active (Fig. 4I, J). Chemogenetic stimulation of VTA*^Sst^* neurons directly increased sleep (Fig. S18). We further defined how VTA*^Sst^* neurons link stress and sleep. We recorded the spontaneous activity of stress-tagged VTA*^Sst^* populations across brain states (Fig. S19A). The tagged cells were also primarily active during NREM and REM sleep (Fig. S19B). Chemogenetic reactivation of SDS-tagged VTA*^Sst^* cells was sufficient to promote NREM and REM sleep (Fig. S19C-E).

To explore if the VTA^*Sst*^→LH pathway links stress and sleep, we conducted fiber photometry to measure terminal activity in the LH responding to stress by expressing GCaMP6 in VTA^*Sst*^ neurons (Fig. S20A). The VTA^*Sst*^→LH projection responded to SDS (Fig. S20B). Following intersectional activity-tagging during SDS (Fig. S20C), chemogenetic reactivation of VTA*^Sst^*→LH pathway promoted sleep (Fig. S20D, E).

**Fig. 5:**
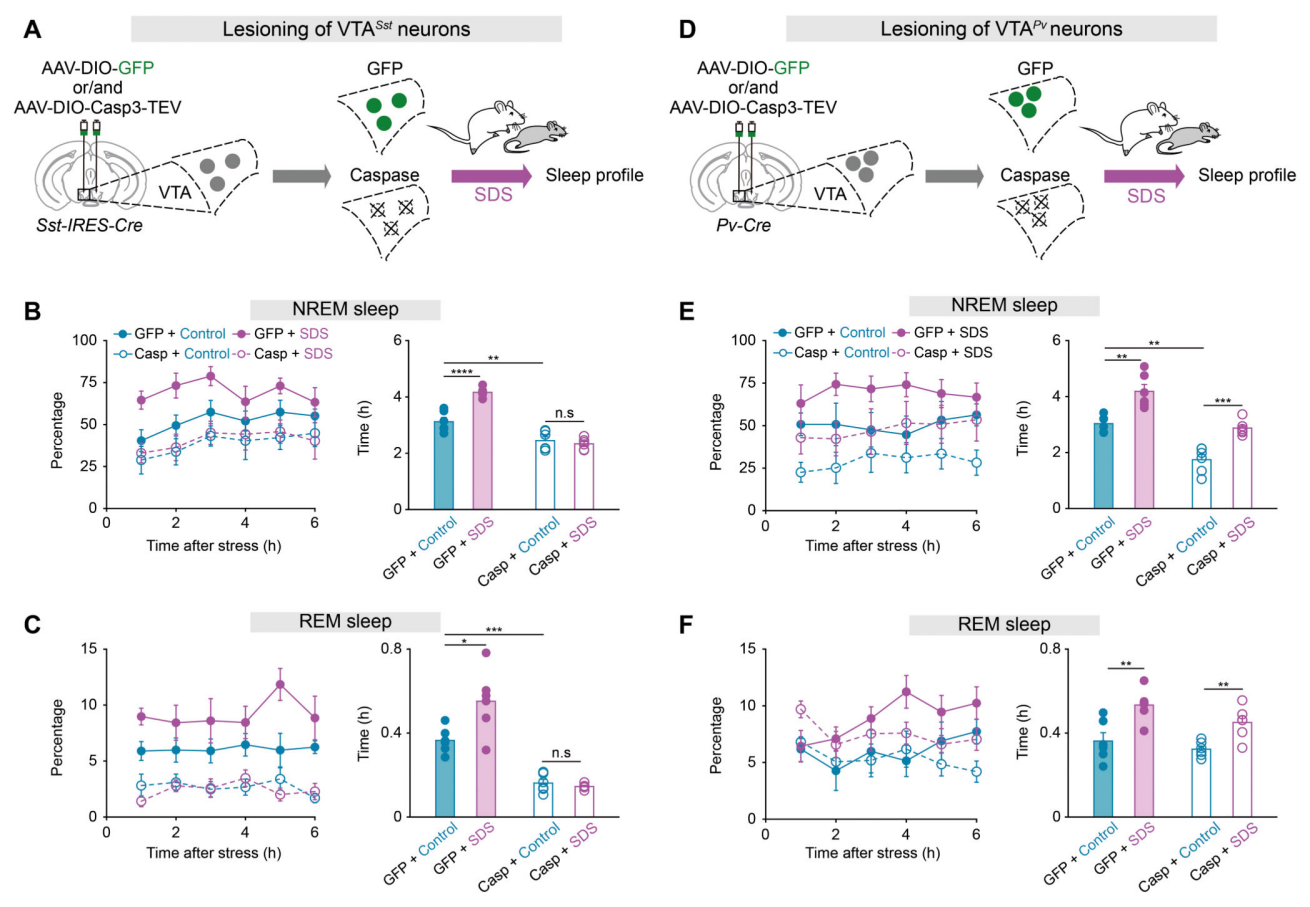
VTA*^Sst^* neurons are necessary for SDS-induced sleep (**A, D**) Genetic ablation of VTA*^Sst^* (**A**) or VTA*^Pv^* (**D**) neurons. (**B, C, E, F**) Percentage and time of NREM or REM sleep in VTA*^Sst^* (**B, C**) or VTA*^Pv^* (**E, F**) ablated mice or control mice given control or SDS. Two-way ANOVA with bonferroni *post hoc* test. *p<0.05, **p<0.01, ***p<0.001, ****p<0.0001, n.s: not significant.

Finally, we examined directly whether VTA*^Sst^* neurons are necessary for SDS-induced sleep. Genetic ablation specifically depleted VTA*^Sst^* neurons (Fig. S21). Lesioning of VTA*^Sst^* neurons decreased baseline sleep (Fig. 5A-C). When VTA*^Sst^*-caspase mice were challenged with SDS, SDS-induced sleep was abolished (Fig. 5B, C). This was also confirmed by chemogenetic manipulation, inhibition of VTA*^Sst^* neurons also decreased SDS-induced sleep (Fig. S22). In contrast, ablation of VTA*^Pv^* neurons decreased baseline NREM sleep, but SDS-induced sleep could still be elicited (Fig. 5D-F).

## SDS-induced sleep by VTA*^Sst^* neurons reduces stress-induced anxiety

**Fig. 6:**
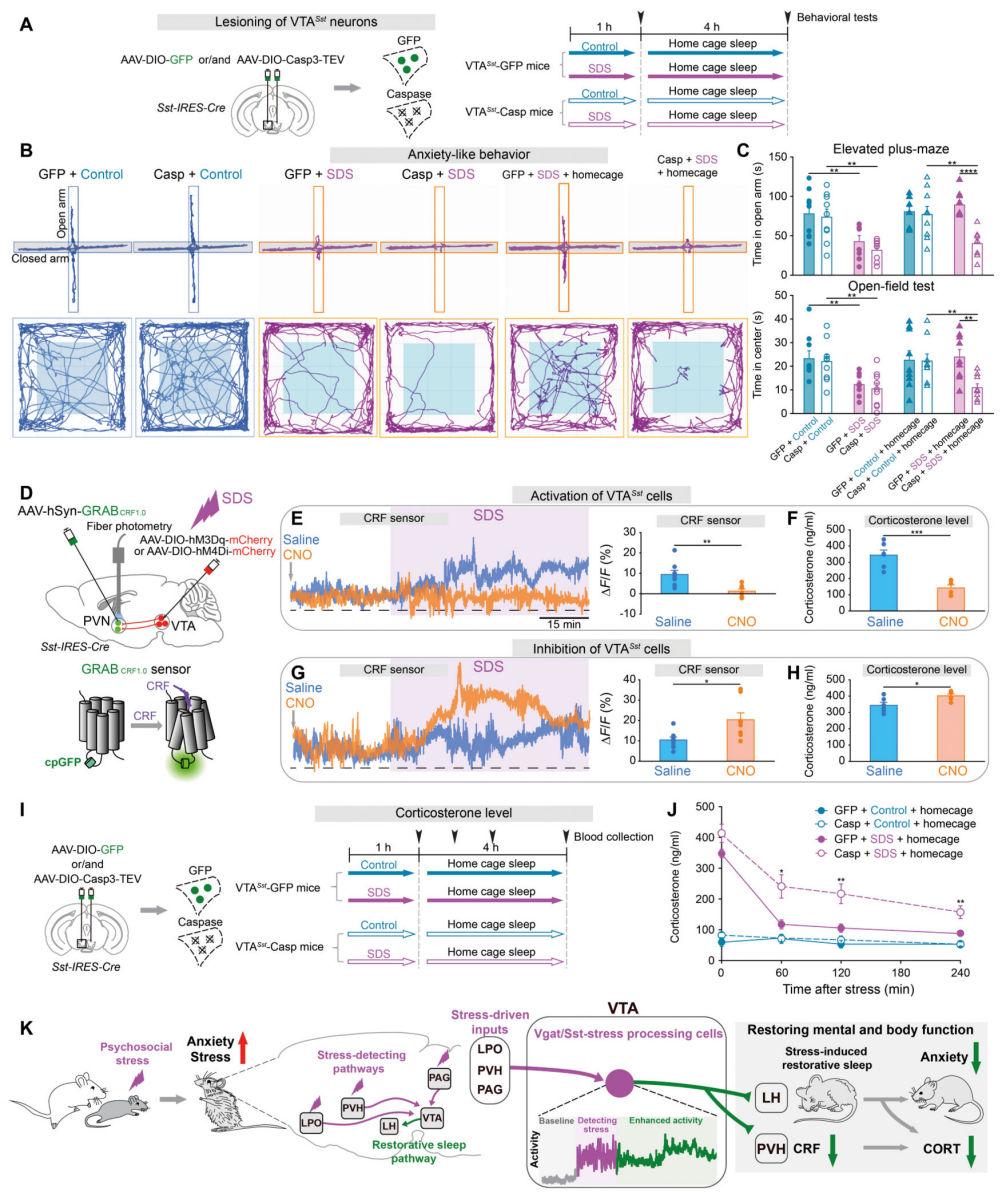
Activity and SDS-induced sleep by VTA*^Sst^* neurons reduces anxiety and corticosterone levels (**A-C**) Plan of the experimental procedure (**A**), tracing of locomotion for representative animals (**B**), time spent in the open arms of the elevated plus-maze and in the center zone during the open-field test (n=9 mice per group) (**C**). (**D**) Delivering genetically-encoded CRF sensor in the PVN hypothalamus alongside chemogenetic manipulation of VTA*^Sst^* neurons. (**E, G**) Raw PVN CRF sensor traces and ΔF/F ratios during SDS after chemogenetic activation (**E**) or inhibition **(G)** of VTA*^Sst^* neurons (n=8 mice per group). (**F, H**) Corticosterone levels following SDS after chemogenetic activation (**F**) or inhibition (**H**) of VTA*^Sst^* neurons (n=6 mice per group). (**I, J**) Plan of the procedure **(I)** and corticosterone levels (n=4-9 mice per group) (**J**). (**K**) Conceptual summary diagram. (**C, J**) Two-way ANOVA with bonferroni *post hoc* test, *p<0.05, **p<0.01, ****p<0.0001. (**E-H**) Unpaired *t*-test, *p<0.05, **p<0.01, ***p<0.001.

Given the proposed restorative function of sleep after SDS (Fig. 1D-G), we explored if this function was linked to VTA*^Vgat-Sst^* neurons. Ablation of VTA*^Sst^* neurons or chemogenetic inhibition of SDS-tagged VTA*^Vgat^* neurons had no effect on baseline anxiety-like behaviors (Fig. S23). However, after SDS, mice lacking SDS-induced sleep (because of selective lesioning/inhibition of VTA*^Sst^* neurons or inhibition of SDS-tagged VTA*^Vgat^* neurons (Fig. 5A-C, Fig. S22 and Fig. 2F-H)) remained in an anxious state (Fig. 6A-C and Fig. S24), similar to the effects of sleep deprivation after SDS (Fig. 1E, F and Fig. S25). When VTA*^Vgat-Sst^* neurons were unimpeded, while the mice had sufficient SDS-induced sleep, the SDS-induced anxiety-like behaviors were reduced to baseline (Fig. 6B, C and Fig. S24). We found that sleep deprivation after SDS suppressed activity in VTA*^Sst^* neurons induced by SDS (Fig. S26). However, during the sleep deprivation procedure after SDS while mice were awake, VTA^*Sst*^ cell stimulation did not reduce anxiety even if VTA*^Sst^* neurons were activated (Fig. S27), suggesting the anxiolytic effects require SDS-induced sleep.

## Activation of VTA*^Sst^* neurons suppresses corticotrophin releasing factor levels induced by SDS

How do VTA*^Sst^* neurons regulate CORT production? VTA*^Sst^* neurons expressing hM3Dq-mCherry sent numerous mCherry-positive axons into the PVN area (Fig. S28A, B), a major site of corticotrophin releasing factor (CRF) production. Following SDS, cells in the PVH are excited, as inferred from their strong expression of cFOS; but stimulation of VTA*^Sst^* neurons inhibited SDS-activated cells in the PVN (Fig. S28C).

We next employed a novel genetically-encoded CRF sensor (*AAV-hSyn-GRAB_CRF1.0_*)(30) to determine the dynamics of CRF release around the PVN (Fig. S28D and Fig. 6D). CRF sensor signals were indistinguishable between control and chemogenetic activation of VTA*^Sst^* neurons (Fig. S28E), consistent with CORT levels also not changing with VTA*^Sst^* stimulation (Fig. S28F). But following SDS, there were large increases in CRF (Fig. S28G). However, chemogenetic activation of VTA*^Sst^* neurons prevented this increase (Fig. 6E), consistent with correspondingly decreased CORT levels (Fig. 6F). On the other hand, chemogenetic inhibition of VTA*^Sst^* neurons further elevated SDS-induced CRF levels (Fig. 6G), thereby increasing CORT levels after SDS (Fig. 6H).

## SDS-induced sleep by VTA*^Sst^* neurons reduces CORT levels

For mice unable to have SDS-induced sleep, either because their VTA*^Sst^* neurons had been ablated or were inhibited (Fig. 6I and Fig. S29A), CORT levels remained higher during their home cage sleep after SDS (Fig. 6J and Fig. S29B), similar to the effects of sleep deprivation after SDS (Fig. 1G). However, when VTA^*Vgat-Sst*^ neurons were unimpeded, the SDS-induced sleep correlated with CORT levels returning to baseline (Fig. 6J and Fig. S29B). In addition, activation of VTA*^Sst^* cells during sleep deprivation after SDS, (i.e. activation of these cells while mice were awake), partially reduced CORT levels (Fig. S29C, D), but the overall CORT levels still remained elevated (Fig. S29D), suggesting that sleep after SDS is also needed to reduce CORT levels.

## Discussion

Our proposed circuit model for how SDS translates to sleep and reduction of anxiety, with VTA*^Vgat-Sst^* cells playing a central role, is shown in Fig. 6K. Once activated by SDS, VTA^*Vgat-Sst*^ cells drive sleep through the lateral hypothalamus, a brain region containing a diverse population of cells implicated in regulating stress, anxiety and sleep/wake behaviours (31, 32). VTA*^Vgat-Sst^* cell activity is maintained for some hours beyond the stress episode, suggesting a form of plasticity which enables them to keep promoting NREM and REM sleep episodes for a sustained period. In parallel to their sleep-inducing and anxiety-reducing effects, VTA^*Vgat-Sst*^cells inhibit CRF-producing neurons in the PVN hypothalamus, thereby reducing CORT levels after SDS. We found that SDS-induced anxiety persisted even in the presence of CORT inhibitors. These results suggest that physiological activation of VTA*^Sst^* neurons during and after SDS represses CRF and therefore CORT production, guarding against overproduction of CORT. Persistently elevated CORT levels have deleterious effects on body organs (1). We propose the reduced anxiety comes from the sleep component. After SDS, the restorative sleep by VTA*^Vgat-Sst^* cells also aids CORT levels returning to baseline, so there seem to be parallel routes to reducing CORT levels, but with VTA*^Sst^* cells coordinating both mechanisms.

The output pathways regulated by VTA*^Vgat-Sst^* cells in the LH to induce sleep and reduce anxiety are unclear. VTA^*Gad67*^ neurons inhibit orexin/Hcrt neurons in the LH (20). However, chemogenetic inhibition of LH^Hcrt^ cells did not reduce anxiety after SDS (Fig. S30), and orexin receptor antagonists did not restore the anxiolytic effects that were missing in VTA*^Sst^*-lesioned mice that had undergone SDS (Fig. S31), suggesting that orexin/Hcrt cell inhibition is not required for the anxiolytic actions of VTA*^Vgat-Sst^* cells. Thus, identifying the targets of VTA*^Vgat-Sst^* cells requires further study. Local action within the VTA of the VTA*^Sst^* neurons is also possible.

In summary, GABA^*Sst*^ neurons in the VTA respond to SDS, an ethological model for psychosocial stress, by inducing restorative sleep and decreasing CRF production. Targeting these neurons could potentially provide a new route for treating anxiety disorders.

## Supplementary Material

Supp Material.Methods

Supp. Fig S1

Supp. Fig S2

Supp. Fig. S3

Supp. Fig. S4

Supp. Fig. S5

Supp. Fig. S6

Supp. Fig. S7

Supp. Fig. S8

Supp. Fig. S9

Supp. Fig. S10

Supp. Fig. S11

Supp. Fig. S12

Supp. Fig. S13

Supp. Fig. S14

Supp. Fig. S15

Supp. Fig. S16

Supp. Fig. S17

Supp. Fig. S18

Supp. Fig. S19

Supp. Fig. S20

Supp. Fig. S21

Supp. Fig. S22

Supp. Fig. S23

Supp. Fig. S24

Supp. Fig. S25

Supp. Fig. S26

Supp. Fig. S27

Supp. Fig. S28

Supp. Fig. S29

Supp. Fig. S30

Supp. Fig. S31

Supp. Fig. S32

Supp. Fig. S33

## Data Availability

All data necessary to understand and assess the conclusions of this study are available in the manuscript or the supplementary materials. Constructs generated in this study have been deposited at Addgene with accession numbers provided in the Supplementary Material.
