# Supplementary material for: A specific circuit in the midbrain detects stress and induces restorative sleep
Source: Science. Author manuscript; Available in PMC 2022 Jul 2. (PMC7612951; doi:10.1126/science.abn0853)
Supplement: Supp Material.Methods [file EMS145530-supplement-Supp_Material_Methods.pdf]

Materials and Methods

Animals

All experiments were performed in accordance with the UK Home Office Animal Procedures Act (1986); all procedures were approved by the Imperial College Ethical Review Committee and the Ethics Committee for Animal Experimentation of the Fourth Military Medical University, Xijing Hospital, Xi'an, and were conducted according to the Guidelines for Animal Experimentation of the Chinese Council institutes. The following strains of mice were used: *Vgat-IRES-Cre*: $Slc32a1^{tm2(cre)Lowl}/J$, JAX stock 016962 and *Vglut2-IRES-Cre*: $Slc17a6^{tm2(cre)Lowl}/J$, JAX stock 016963, both generated by B.B. Lowell (*33*); *Sst-IRES-Cre*: $Sst^{tm2.1(cre)Zjh/J}$, JAX stock 013044, generated by Z. J. Huang (*34*); *Pv-Cre*: *B6.129P2-*$Pvalb^{tm1(cre)Arbr/J}$, JAX stock 008069, kindly generated by S. Arber (*35*), and Ai9 (*Rosa-LSL-tdTomato*), JAX stock 007909, generated by H. Zeng (*36*); *Hcrt-IRES-Cre;* $Hcrt^{tm1.1(cre)Ldl}$, Stanford_006_mHcrt_1B1, generated by Luis de Lecea (*37*) and kindly provided by Zhian Hu (Third Military Medical University, Chongqing, China) (*38*). CD-1 mice, 6–8 months old and C57BL/6j mice were purchased from Charles River Laboratories, Inc., and Beijing Vital River Laboratory Animal Technology, Beijing, China. Mice were maintained on a 12 h:12 h light:dark cycle at constant temperature and humidity with *ad libitum* food and water.

AAV vectors, AAV production, and rabies system

Plasmid *pAAV-cFOS-tTA* (Addgene plasmid#66794) was generated in our lab as previously described (*39*). *pAAV-TRE-DIO-hM3Dq-mCherry* (Addgene plasmid#115161) (*39, 40*) and *pAAV-TRE-DIO-hM4Di-mCherry* (Addgene plasmid#182532) were constructed in our lab, where the *hM3Dq-mCherry* or *hM4Di-mCherry* reading frame is inverted between heterologous pairs of lox sites (double inverted orientation, DIO) downstream of the *TRE* (Tet-Response-Element) promoter (Clontech). To make this plasmid, we did the following: the

promoter *TRE* fragment was cut out and isolated from *pAAV-TRE-hM3Dq-mCherry* plasmid that we made before (Addgene plasmid #66795) using MluI and SalI restriction enzymes. The plasmid *pAAV-hSyn-DIO-hM3Dq-mCherry* or *pAAV-hysn-DIO-hM4Di-mCherry* (gift from Bryan L. Roth, Addgene plasmid #44361(*41*) or #50475) was double-digested with MluI and SalI to remove the *hSynapsin* promoter. The *TRE* promoter was ligated into the backbone between MluI and SalI sites to give *pAAV-TRE-DIO-hM3Dq-mCherry* or *pAAV-TRE-DIO-hM4Di-mCherry*, respectively. To make *pAAV-TRE-DIO-ChR2-EYFP* (Addgene plasmid#183765), the backbone, *AAV-TRE-DIO-*, was purified from *pAAV-TRE-DIO-hM3Dq-mCherry-*, after double digestion with AscI + NheI and gel purification. The insert, *ChR2-EYFP*, was taken from Addgene plasmid #20298 (humanized ChR2 with H134R mutation fused to EYFP, gift from Karl Deisseroth), after double digestion with AscI + NheI and gel purification. Both fragments were ligated to produce the final construct *pAAV-TRE-DIO-ChR2-EYFP*. The plasmid *pAAV-hSyn-DIO-GCaMP6s* was created as we previously described (*42*); we used the GCaMP6s reading frame from *pGP-CMV-GCaMP6s* (gift of Douglas Kim, Addgene plasmid #40753) (*43*). Then, to make *pAAV-TRE-DIO-GCaMP6s* (Addgene plasmid#183809), *pAAV-hSyn-DIO-GCaMP6s* and *pAAV-TRE-hM3Dq-mCherry* were digested with MluI and SalI. After double digestion, the 6.2 kb band corresponding to the backbone (*AAV-MluI-...-SalI-DIO-GCaMP6s*) was gel purified, leaving behind the hSyn promoter; similarly, the *MluI-TRE-SalI* promoter was gel purified (band around 344bp). The two fragments were ligated to produce *pAAV-TRE-DIO-GCaMP6s*. Plasmid *pAAV-fDIO(FLP-Cre-dependent)-hM3Dq-mCherry* was a gift from Kevin T. Beier generated in Robert C. Malenka's lab (*44*). *pAAV-TRE-DIO-Casp3-TEV* was generated in our lab. To make *pAAV-TRE-DIO-Casp3-TEV* (Addgene plasmid#183766), the backbone *AAV-DIO-Casp3-TEV* was gel purified from *pAAV-hsyn-DIO-Casp3-TEV* (Addgene plasmid #45580) (*45*) after double digestion with MluI and SalI to remove the *h-synapsin* promoter. At the same time, TRE was

also gel purified from *AAV-TRE-hM3Dq-mCherry* after double digestion with MluI and SalI. The two fragments were ligated to give *pAAV-TRE-DIO-Casp3-TEV*.

We packaged the transgenes (*AAV-DIO-ChR2-EYFP*, *AAV-DIO-GCaMP6s*, *AAV-cFOS-tTA*, *AAV-TRE-DIO-hM3Dq-mCherry*, *AAV-TRE-DIO-hM4Di-mCherry*, *AAV-TRE-DIO-ChR2-EYFP*, *AAV-TRE-DIO-GCaMP6s*, *AAV-TRE-DIO-FLPo*, *AAV-TRE-DIO-Casp3-TEV* and *AAV-fDIO-hM3Dq-mCherry*) into AAV capsids (capsid serotype 1/2) in house as described previously (*46*). To produce retro-*AAV-TRE-DIO-FLPo*, we used the AAVpro Purification Kit (all Serotypes) (Takara-Clontech, Cat#6666) and the rAAV2 packaging plasmid, a gift from Alla Karpova and David Schaffer (Addgene plasmid #81070) (*47*), and the helper plasmid *pFΔ6*, together with *AAV-TRE-DIO-FLPo* from Minmin Luo (Addgene plasmid #118027) (*48*).

For the rabies system, *pAAV-DIO-N2cG*, *pAAV-DIO-TVA-nGFP* and *RABV-N2c□G-EnvAmCherry* were kindly provided by Andrew Murray (Sainsbury Wellcome Center, UCL, UK) (*49*). *AAV-DIO-GFP* was purchased from Addgene (#50457-AAV2). *AAV-DIO-hM3Dq-mCherry*, *AAV-DIO-hM4Di-mCherry*, *AAV-DIO-taCasp3-TEVp* and *AAV-DIO-mCherry* were provided by BrainVTA Technology Co., Ltd. (Wuhan, China).

Surgery

The surgical procedure was conducted as previously described (*42*). 10-14-week-old male mice were anesthetized with 2% isoflurane in oxygen by inhalation and received buprenorphine (0.1 mg/kg) and carprofen (5 mg/kg) injections, and then placed on a stereotaxic frame (Angle Two, Leica Microsystems, Milton Keynes, Buckinghamshire, UK). The AAVs were injected through a stainless steel 33-gauge/15mm/PST3 internal cannula (Hamilton) attached to a 10 µl Hamilton syringe at a rate of 0.1 µl min^{-1}. For the AAV injections, the virus was bilaterally or

unilaterally injected into the VTA, LH, LHb, DG or CeA. The injection coordinates and volumes were:

VTA: ML = ± 0.35 mm, AP = - 3.52 mm, DV = - 4.25 mm; 50 nl + 50 nl

LH: ML = ± 1.00 mm, AP = - 1.56 mm, DV = - 5.20 mm; 100 nl + 100 nl

After injection, the cannula was left at the injection site for 5 min and then slowly pulled out. After injections, mice that were to undergo the sleep experiments were implanted with three gold-plated miniature screw electrodes (–1.5 mm Bregma, +1.5 mm midline; +1.5 mm Bregma, –1.5 mm midline; –1 mm Lambda, 0 mm midline – reference electrode) with two EMG wire (AS634, Cooner Wire, CA). The EMG electrodes were inserted between the neck musculatures. EEG-EMG connector for the Neurologger 2A was affixed to the skull with Orthodontic Resin power and Orthodontic Resin liquid (Tocdental, UK).

For the telemetry EEG and EMG surgery, the mice were anesthetized with isoflurane as above and implanted in the abdominal subcutaneous with wireless TL11M2-F20-EET device (Data Sciences International) biotelemetry transmitters. Four of the wires were attached subcutaneously to the neck of the mice through a guide cannula. Then, mice were placed into a stereotaxic instrument in a prone position. Next, two surface EEG electrodes (biopotential leads tethered to miniature stainless-steel screws) were implanted over the left frontal lobe (AP 0.2 mm, ML 1.5 mm, DV −0.1 mm) and the contralateral parietal lobe (AP −1.7 mm, ML −0.2 mm, DV −0.1 mm) and lowered until they contacted the dura of the skull, with dental cement to secure in place. Two biopotential EMG electrodes were embedded through the small incision, which was made at the oblique cervical muscle with a 21G needle and fixed with insoluble silk sutures. The incision site was sutured and treated with local anesthetic (2% lidocaine) and topical antibiotic. For EEG/EMG recording, mice were individually housed following surgery in standard Plexiglas home cages placed on RPC-1 PhysioTel receivers,

which receive signals from the transmitters. RPC-1 PhysioTel receivers are concatenated to a data exchange matrix, which transmits continuous and synchronized EEG, EMG and motor activity.

For the fiber photometry or optogenetic experiments, after virus injection, mice received unilateral surgical implantations above the VTA, LH, LHb, DG or CeA of a monofiberoptic cannula (200 µm; Doric Lenses, Inc., Quebec, Canada). The fiber placement coordinates were:

VTA: ML = 0.35 mm, AP = - 3.52 mm, DV = - 3.9 mm

LH: ML = 1.00 mm, AP = -1.56 mm, DV = -5.10 mm

LHb: ML = 0.5 mm, AP = - 1.6 mm, DV = - 2.3 mm

DG: ML = 1.3 mm, AP = - 2.2 mm, DV = - 1.6 mm

CeA: ML = 2.7 mm, AP = - 1.4 mm, DV = - 4.15 mm

Cannula Implantation and administration of orexin receptor antagonists

Mice were placed in an induction chamber with 1.2-1.4% isoflurane (Baxter Healthcare, Puerto Rico) vaporized by oxygen flowing at 1.0 L/min and then transferred and fixed to a stereotaxic frame while keeping the mice anesthetized by 0.8% isoflurane via a mask. After cleaning and and exposure of the skull, *AAV-DIO-taCasp3-TEV* was injected into the VTA of *Sst-IRES-Cre* mice as described above. Three weeks after virus injection, a guide cannula (Stoelting, Co., US) was stereotaxically directed into the lateral cerebral ventricle (ML = −1.00 mm, AP = 0.50 mm, DV = −2.00 mm). The mice were allowed to recover for at least 7 days after the surgery.

Behavioral protocols

Social defeat stress, the non-stressful procedure, restraint stress, exposure to novel environments, voluntary wheel running, forced treadmill running and activity-tagging all took place one hour before the start of "lights on". The cage-change mild stress procedure was conducted at the beginning of "lights on". Mice were habituated for 10 min in the experimental room for a continuous period of 3 days before the experiments. Open-field tests (OFT) and elevated plus-maze (EPM) were performed at specific time points below: 1, before control or SDS (1-h before the start of "lights on"); 2, straight after control or SDS (at the beginning of "lights on"); 3, after control or SDS followed by 4-h home cage sleep or 4-h sleep deprivation (4-h after "lights on").

Social defeat stress was performed as reported with small modifications (Fig. S1A) (*50*). Before performing social defeat experiments, male CD-1 retired breeder mice at 4–6 months of age, were singly housed, allowing habituation for a minimum of 7 d prior to screening. The appropriate CD-1 aggressive mice were selected from the 3-d screening process to meet the social defeat criterion (*51*). For screening the aggressors, C57BL/6j mice between 8 and 20 weeks of age were used. The CD-1 mice that showed extreme aggressive behavior or no aggression were excluded from experiments. Only mice showing aggression within 1 min and persistent aggressive intention were used in social defeat sessions (*51*).

For social defeat stress, an intruder mouse (experimental mouse) was introduced into the home cage of the CD1 resident mouse for 5 min. During these 5 min, there was usually 5-8 conflicts. Then a transparent partition was used to separate the intruder and resident mice, but the intruder remained in olfactory, visual and auditory contact with the resident for 10 min. During the social defeat sessions, the procedure was repeated four times at 15 min intervals, for a total of 60 min (Fig. S1A). At the end of the stress procedure, the intruder exhibited an apparent freezing behavior or a submissive posture. Normally, no wound was found on intruders

following this stress procedure; however, when the intruder mouse had visible wounds, the intruder and corresponding CD-1 mouse were excluded. For the controls, the intruder mouse was placed into the home cage of the aggressive resident mouse without contact for 60 min using a transparent partition to separate the intruder and resident mice, but remained in olfactory, visual and auditory contact with the resident (Fig. S1A). Video recording was performed with recordings of fiber photometry signals.

Non-stressful procedure. This protocol was used as the control for the physical movements during the social defeat stress. In detail, an intruder mouse (experimental mouse) was introduced into the home cage of the juvenile (younger) mouse for 5 min. During these 5 min, the mice experienced movements, physical activity and social interaction but typically had no conflicts. If the mice had any conflicts, the intruder and corresponding juvenile mouse were excluded. Then a transparent partition was used to separate the intruder and resident mice, but the intruder remained in olfactory, visual and auditory contact with the resident for 10 min. During the non-stressful sessions, the procedure was repeated four times at 15 min intervals, for a total of 60 min (Fig. S1B). At the end of the non-stressful procedure, the intruders exhibited normal behaviors without having freezing behavior or a submissive posture. For the controls, the intruder mouse was placed into the home cage of the juvenile resident mouse without contact for 60 min using a transparent partition to separate the intruder and resident mice, but remained in olfactory, visual and auditory contact with the resident (Fig. S1B).

Restraint stress. The restraint paradigm was performed as previously described (*7*). Mice were individually placed into a well-ventilated 50-ml Falcon conical tube with a narrow open window on top to allow for movement and sliding of the fiber patch cord. During the entire restraint procedure, mice were in a natural body position without physical harm. For the control experiment of long-term fiber photometry, mice were placed into a 5L glass cylinder for 60 min without any restraint.

Cage-change mild stress produce was conducted as reported (*52*). At the beginning of ‘‘lights on’’, animals’ home cages were exchanged with new cages with fresh bedding and food in the holding room. For the control experiment of long-term fiber photometry, mice were
in the home cages for 60 min.

Novel environments. Mice were placed in a novel cage with a novel object in the experimental room for 60 min (Fig. S1C). For the controls, the mice were in their home cages in the holding room (Fig. S1C).

Novel objects. An object was placed in the resident mouse’s home cage in the experimental room. Video recording was performed with recordings of fiber photometry signals.

Mild sleep deprivation was performed as we previously reported (*39*). Mice were sleep deprived at the start of “lights on” for 4-h using novel cages and novel objects. When mice showed signs of drowsiness, a soft brush was used to touch the mouse for 1-2 s.

Voluntary wheel running. Wheel running exercise experiments were performed using activity cages (37 (h) x 26 (w) x 35 (d) cm) with a running wheel installed (diameter 25 cm) (Shanghai Yuyan Instruments Co., Ltd.). Animals were acclimatized to the activity cage for 10 min on three consecutive days before the experiments. Mice were allowed voluntary wheel running for 60 min. For the control group, animals remained sedentary in their home cages in the behavioral room. For fiber photometry experiments, we used a cage-free running wheel on the table. The mice run freely on the wheel for 60 min with fiber photometry recorded.

Forced treadmill running. The treadmill exercise regimen was adapted from previous study (*53*). To reduce the amount of stress to the animals, prior to the running experiment, mice were acclimatized to the stationary treadmill apparatus (Shanghai Yuyan Instruments Co., Ltd.) for 10 min, increasing to a speed of 4-10 m/min on three consecutive days. After this adaption period, mice experienced exercise training for 60 min, with the treadmill set at a speed of 6 m/min. The protocol corresponded to approximately 50% of maximal oxygen consumption.

Open-field tests

The Open Field Test (OFT) was used to test the anxiety-like behaviors of mice as reported (*54*). The mice were gently placed in a 40 cm × 40 cm × 40 cm open-field, which was invisibly divided into 25 small squares. During the experiment, mice were placed in the central square and then allowed free movement in the open field with real-time position tracking and video recording for 10 minutes. The average speed and time in the center area within the last 5 min were captured and analyzed using an ANY-maze 7.0 (Stoelting, USA).

Elevated plus-maze

The Elevated Plus-Maze test (EPM) was used to test the anxiety-like behaviors of the mice, as we previously performed (*55*). Following the Open Field Test, the experimental mice were taken out and placed in the central area of the elevated plus-maze. The head and upper limbs of mice were randomly placed in the open arm, and then mice moved freely on the elevated platform for 5 min. At the end of the test, we cleaned each arm of the elevated plus-maze and eliminated the odor with 20% alcohol. The position track and real-time performance of mice during the EPM test were recorded. Time in the Open Arm was analyzed using the ANY-maze 7.0 (Stoelting, USA).

Activity-tagging

The activity-tagging procedure was performed as we previously reported (*39, 40, 56*). *Vgat-IRES-Cre* or *Sst-IRES-Cre* mice were placed on doxycycline (200 mg/kg) (Envigo TD.09265) for at least one week prior to the virus injection. Mice were injected with the activity-tagging transgenes. 2-4 weeks after injection, mice were taken off doxycycline 24-h before activity-tagging (mice were kept undisturbed during this period). VTA^{Vgat} or VTA^{Sst} neurons were allowed to become tagged while mice experienced 1-h social defeat (SDS), or the non-stressful

procedure, or voluntary wheel running, or forced treadmill running, or restraint stress, or cage-change or novel environments (novelty). For the control tagging, mice experienced 1-h control for social defeat or non-stressful procedure (using a transparent partition to separate the intruder and resident mice) (**Behavioral protocols – social defeat stress and non-stressful procedure**). To shut down the activity-tagging system, mice were put back on doxycycline for 5 to 7 days (without any disturbance for the first 2 days) before anatomical or behavioral assessments.

Assessment of the activity-tagging system

We assessed the efficacy and specificity of the activity-tagging by delivering transgenes into the VTA (**Fig. S32A**) or intersectionally into the VTA → LH pathway (**Fig. S32B**).

The efficacy of Dox: activity-tagging AAVs were injected into the VTA (**Fig. S32A**) or VTA → LH pathway (**Fig. S32B**) of *Vgat-IRES-Cre* mice. After injection, mice were constantly on Dox while tagged by SDS (On Dox → On Dox → SDS-tagging → On Dox). We did not observe many tagged cells, suggesting Dox is sufficient to inhibit the expression of the transgenes.

The specificity of tagging for SDS: activity-tagging AAVs were injected into the VTA (**Fig. S32A**) or VTA → LH pathway (**Fig. S32B**) of *Vgat-IRES-Cre* mice. 24-h before activity-tagging, Dox was removed, and mice underwent SDS, control or home cage procedure. After activity-tagging, mice were put back on dox for 5 to 7 days (On Dox → Off Dox → SDS-, control- or home cage-tagging → On Dox). Compared to SDS-tagging, we did not observe many control- or home cage-tagged cells, suggesting the cells that have been labeled in the experiments were specific for SDS.

Fiber photometry

Fiber photometry was performed as described (*42, 57*). In detail, a Grass SD9 stimulator was used to control a 473-nm Diode-pumped solid-state (DPSS) blue laser with a fiber coupler (Shanghai Laser & Optics Century Co., Shanghai, China). The laser light was passed through a single source fluorescence cube (FMC_GFP_FC, Doric Lenses, Quebec, Canada) through an optical fiber patch cord (Ø 200 μm, 0.22 NA, Doric Lenses). From the filter cube, a multimodal optical patch cord (Ø 200 μm, 0.37 NA, Doric Lenses) was connected to the mouse chronically implanted fiber (Ø 200 μm, 0.37 NA) with a ceramic split mating sleeves ferrules (Thorlabs, Newton, New Jersey). The GCaMP6 output was then filtered at 500-550 nm using a second dichroic in the fluorescence cube and converted to a voltage by an amplified photodiode (APD-FC, Doric Lenses). The photodiode output was amplified by a lock-in amplifier (SR810, Stanford Research Systems, California, USA), also used to drive the laser at 125 Hz with an average power of 80 μW at the fiber tip. The signal was then digitized using a CED 1401 Micro box (Cambridge Electronic Design, Cambridge, UK) and recorded at 1 kHz using Spike2 software (Cambridge Electronic Design, Cambridge, UK).

The photometry signals during the stress experiences were matched with video recordings. For each experiment, the photometry signal F was converted to ΔF/F by ΔF/F(t)=(F(t)-median (F))/median (F) (*42*). Mice with an implanted optical fiber were connected to a patch cord for recording of the Ca^{2+} signal. After connection, the mice were habituated in their home cages, novel cages or other experimental cages for 30 min. In some recordings, we observed decay of the photometry signal at the beginning of the recordings. All the trials were conducted after the photometry signal became stable. For photometry recordings across brain states (vigilance states), the photometry signals were matched with EEG/EMG signals. The photometry signals were not due to movement, as the GFP signal originating from control *AAV-DIO-GFP* expression in VTA^{Vgat} neurons did not change during the stress (Fig. S33).

Fiber photometry to measure CRF

The $GRAB_{CRF1.0}$ sensor was developed by Yulong Li' s laboratory (Peking University, China) (30).

AAV-hSyn-CRF1.0-WPRE-hGHpA was packaged by WZ Biosciences Inc (Shandong, China). *Sst-IRES-Cre* mice were anesthetized and fixed to the stereotaxic apparatus. The AAV expressing the CRF sensor was injected into the PVH (AP = -0.80 mm, ML = 0.20 mm, DV = -4.15 mm). Meanwhile, *AAV-DIO-hM3Dq/hM4Di-mCherry* was injected into the VTA using a Nanoject II apparatus (Drummond Scientific, Broomall, PA) at a rate of 23 nL/min. After virus injection, an optical fiber (diameter: 200 μm) was implanted into the PVH (AP = -0.80 mm, ML = 0.20 mm, DV = -4.1 mm). Three weeks after implantation of the optical fiber in the PVH, fiber photometry recordings were performed for social defeat stress experiments (detected by QAXK-FPS-DC-LED, ThinkerTech Nanjing Bioscience Inc).

Chemogenetic protocols

We used the clozapine-N-oxide (CNO) chemogenetic method (*58*). CNO (C0832, Sigma-Aldrich, dissolved in saline, 1 mg/kg) or saline was injected *i.p.* Mice were split into random groups that received either saline or CNO injection. To test the sleep-promoting effects, we injected saline or CNO during the "lights off" period when the mice were in the active phase. In detail, for the activity-tagged VTA^{Vgat} neurons or activity-tagged VTA^{Sst} neurons with hM3Dq-mCherry, saline or CNO was given during the "lights off" period. For non-tagged VTA^{Sst} neurons expressing hM3Dq-mCherry, saline or CNO was given during the "lights off" period.

To chemogenetically reactivate activity-tagged LH-projecting VTA^{Vgat} neurons or VTA^{Sst} neurons, saline or CNO was given during the "lights off" period. For the activity-tagged VTA^{Vgat} neurons with hM4Di-mCherry, saline or CNO was given 30 min before stress

producers. Mice experienced SDS 1-h before the “lights on” period. Therefore, saline or CNO was given to these mice 90 min before the “lights on” period.

Optogenetic protocols

We used ChR2-based optogenetics (*59*). For optogenetic experiments, after the fiber patch cord was connected to the laser generator, the optical power meter (SANWA Electric Instrument, Tokyo, Japan) was used to measure laser intensity, to which the optic fiber was attached to obtain laser intensity at 10-15 mW. Then, the optic fibers on the head of mice were concatenated to the fiber patch via the rotary joint (ThinkerTech, Nanjing, China). During the experiments, the mice were stimulated optically (ChR2, 473 nm, 20 Hz, 30ms duration) every 60 s with 30 s interval. To test the sleep-promoting effects, opto-stimulation was conducted when the mice were awake.

Sleep-wake behavior and EEG analysis

Mice were attached with mock Neurologgers before experiments. EEG and EMG signals were recorded using Neurologger 2A devices (*60*). Wake, NREM or REM sleep was automatically classified at 5-s epoch using sleep analysis software Spike2 (Cambridge Electronic Design) and then manually re-scored. Episode duration was analyzed using MATLAB (R2020b) as we previously described (*42*).

For telemetry EEG and EMG, EEG data were analyzed using the NeuroScore Software (DSI, Harvard Bioscience, Inc). Vigilance states, including NREM sleep, REM sleep, and wake states, were first automatically assigned to each 5-s epoch using an automated scoring algorithm and then manually scored. The duration and bouts of vigilance state were calculated. Meanwhile, spectral power was quantified from the raw EEG data through a multitaper method

for each vigilance state. Analysis was performed using a custom MATLAB script via the Chronux toolbox (http://chronux.org) (*61*).

Immunohistochemistry

Mice were transcardially perfused with 4% paraformaldehyde (Thermo scientific) in phosphate-buffered saline (Sigma). Brains were post-perfused with 4% paraformaldehyde in PBS for 24-h, and subsequently transferred in 30% sucrose/PBS. 40-µm-thick coronal sections were cut using a cryostat microtome (Leica CM1900, Germany) or a sliding microtome (HM 450, Cambridge Scientific). Free-floating sections were washed in PBS three times for 5 min, permeabilized in PBS plus 0.4% Triton X-100 for 30 min, blocked by incubation in PBS plus 5% normal goat serum (NGS) (Vector), 0.2% Triton X-100 for 1-h. Sections were incubated with primary antibody diluted in PBS plus 2% NGS overnight at 4°C in a shaker. Incubated brain sections were washed three times in PBS for 10 min and incubated for 2 hours with secondary antibody in PBS and subsequently washed 4 times in PBS for 10 min (all at room temperature). For the cFOS staining, the mice were perfused 90 min after SDS or control procedure. To examine the activation of VTA^{Sst} neurons by CNO during sleep deprivation, the mice were perfused 3 hours after saline or CNO injection (**Fig. S27B**).

Primary antibodies: Rabbit polyclonal cFOS (1:3000, ABE457, Millipore, UK); Rat monoclonal mCherry (1:1000, M11217, ThermoFisher); Rabbit polyclonal GFP (1:1000, A-11122, ThermoFisher); and Mouse monoclonal TH (1:2000, T2928, Sigma); Rabbit polyclonal GABA (1:200, GeneTex); Rat monoclonal somatostatin (1:1000, Merck); Mouse monoclonal orexin (OA) (1:200, MAB763, R&D Systems); Rabbit polyclonal cFOS (1:1000, Sigma-Aldrich)

Secondary antibodies were Alexa Fluor 488 goat anti-rabbit, Alexa Fluor 488 goat anti-mouse, Alexa Fluor 594 goat anti-rabbit, Alexa Fluor 594 goat anti-mouse and Alexa Fluor 594 goat

anti-rat (1:1000, Invitrogen Molecular Probes, UK). Alexa Fluor 488 donkey anti-rabbit, Alexa Fluor 488 donkey anti-mouse, Alexa Fluor 594 donkey anti-rabbit, Alexa Fluor 594 donkey anti-mouse, and Alexa Fluor 594 donkey anti-rat (1:1000, Jackson ImmunoResearch Laboratories). For the Ai9 reporter mouse, tdTomato was detected with primary fluorescence without antibody staining.

Slices were mounted on slides, embedded in Mowiol (with DAPI) (Vector Labs, H-1200), cover-slipped (BIOTIUM), and analyzed using an upright fluorescent microscope (Nikon Eclipse 80i or 90i Nikon Corporation, JAPAN) or a Zeiss LSM 510 inverted confocal microscope or a Leica SP5 MP confocal microscope (Facility for Imaging by Light Microscopy, FILM, Imperial College London).

Enzyme immunoassay of serum corticosterone

Mice were decapitated immediately at each time point after the non-stressful procedure, SDS, restraint stress or novel environments, and their control protocols, respectively. Blood was rapidly collected in 1.5-ml plastic tubes placed in ice and centrifuged at 1,500 × g for 20 min, and serum was collected and stored at −80°C until use. Serum samples were tested for corticosterone concentration using the Enzo ELISA kit (ADI-900-097; Enzo Life Sciences, Exeter, Devon, UK) according to the manufacturer's instructions.

Metyrapone

Metyrapone (Merck) was administrated (*i.p*) as reported (*62*). Metyrapone was dissolved in 5% ethanol saline solution and injected *i.p* (50 mg/kg) into the mice 30 min before the SDS.

Orexin/Hcrt receptor antagonists

Infusion of orexin receptor antagonists (*i.c.v*) was performed as previously described (*63*). Before the experiments, SB-334867 (Tocris Bioscience, USA), Orexin1 receptor antagonist,

and TCS-OX2-29 (Tocris Bioscience, USA), Orexin2 receptor antagonist, were dissolved in 5% DMSO at 33.3 μg/μL. SB-334867 or TCS-OX2-29 was infused via *i.c.v.* at 0.1 μL/min. Injector cannula were connected with micro-syringe with Polyethylene pipe filled with mineral oil. 500 nL of SB-334867 or TCS-OX2-29 was infused into the lateral cerebral ventricle through the injector cannula, respectively. Mice were allowed to move freely in their home cage during infusions. Afterwards, cannulas were left in place for an additional 2–3 min to allow drug diffusion. 20 min after drug infusion, SDS or behavioral tests were conducted.

The double Orexin/Hcrt receptor antagonist MK6096 (filorexant, Cambridge Bioscience) was dissolved in 50% saline/50% PEG-400 administered *i.p.* at a dose of 5.0 mg/kg 30 min before the behavioral study, as previously reported (*37*).

Single-cell RT-qPCR from acute brain slice of the VTA

Single-cell RT-qPCR was performed as we previously described (*42, 56*). SDS-tagged VTA^{Vgat} or VTA^{Sst} mice were killed by cervical dislocation. The brains were quickly removed and placed into cold oxygenated N-Methyl-D-glucamine (NMDG) solution (NMDG 93 mM, HCl 93 mM, KCl 2.5 mM, NaH2PO4 1.2 mM, NaHCO3 30 mM, HEPES 20 mM, glucose 25 mM, sodium ascorbate 5 mM, Thiourea 2 mM, sodium pyruvate 3 mM, MgSO4 10 mM, CaCl2 0.5 mM). Coronal brain slices (200-μm thickness) encompassing the VTA were obtained using a vibratome (VT1200S, Leica). Slices were kept in NMDG solution at 33°C for 15min with constant oxygenation and transferred to fully oxygenated standard ACSF (NaCl 120 mM, KCl 3.5 mM, NaH2PO4 1.25 mM, NaHCO3 25 mM, glucose 10 mM, MgCl2 1 mM, CaCl2 2 mM) and were maintained in a chamber that was gently and continuously aerated with carbogen gas for at least 45 min at room temperature (20–22 ^{o}C). Slices were transferred to a submersion recording chamber and were continuously perfused at a rate of 3-4 ml/ min with fully oxygenated ACSF at room temperature.

The brain slices were visualized with a fixed upright microscope (BX51WI, Olympus) equipped with a water immersion lens (40×/0.8 W) and a digital camera (C13440, Hamamatsu). Patch pipettes were pulled from borosilicate glass capillary tubes using a pipette puller (P97, Sutter). The resistance of pipettes varied between 3 and 5 MΩ. The patch pipettes were filled with RNase-free intracellular solution containing (in mM): 140 K-gluconate, 5 NaCl, 10 HEPES, 0.1 EGTA, 2 MgCl2, 2 Mg-ATP, and 0.3 Na-GTP (pH 7.35, osmolality 285 mOsm). The hM3Dq-mCherry-positive neurons were visually identified and randomly selected. The internal solution in the patch pipette was limited up to 1 μl.

The single-cell RT-PCR assays were performed using the Single Cell-to-CT Kit (Thermo Fisher Scientific). The cytoplasm was aspirated into the patch pipette and expelled into a PCR tube containing lysate buffer. The content of the tagged neuron was aspirated into the recording pipette and expelled into cell lysis/DNase I solution. Reverse transcription and cDNA pre-amplification were performed according to the kit protocol. qPCR was performed using the QuantStudio Real-Time PCR system (Thermo Fisher Scientific). The mouse TaqMan assay probes were designed by TaqMan® Gene Expression Assays (Thermo Fisher Scientific) and purchased from Thermo Fisher Scientific. *18srRNA*, Mm03928990_g1; *Slc17a6* (*Vglut2*), Mm00499876_m1; *Slc6a3* (*dat*), Mm00438388_m1; *Gad1*, Mm04207432_g1; *Slc32a1* (*Vgat*), Mm00494138_m1; *Sst*, Mm00436671_m1; *Pv*, Mm00443100_m1; *Vip*, Mm00660234_m1. The single-cell gene expression matrix was made in Origin.

Quantification and statistics: Mice were assigned randomly to the experimental and control groups. For chemogenetic experiments, saline or CNO injections were blinded. For optogenetic experiments, control-tagged or stress-tagged groups were blinded. The analysis of EEG data and measurement of ELISA were done blinded. Animal behavior data that was scored from videos with fiber photometry was not blinded. The individual tests and the number (n) of mice

for each experimental group are shown in the figure legends. A normality test was performed on each dataset using the Shapiro-Wilk test. The parametric tests (paired, unpaired *t*-tests, one-way or two-way ANOVA and *post hoc* test) were used if the datasets were normally distributed. The Mann–Whitney U-test was used for non-parametric tests. All data are given as mean ± SEM. Statistical tests were run in “Origin 2021” (Origin Lab).

Fig. S1 Behavioral procedures of SDS, non-stressful procedure, voluntary wheel running, forced treadmill running and novel environment (Related to Fig. 1)

Plan of the SDS / control (**A**), non-stressful procedure / control (**B**), voluntary wheel running / control (**C**), forced treadmill running / control (**D**) and novel environment / control (**E**) experimental procedures. See the 'Behavioral protocols' section in Materials and Methods.

Fig. S2 NREM and REM sleep parameters after SDS, non-stressful procedure, voluntary wheel running, forced treadmill running and novel environments (Related to Fig. 1)

(**A**) The experimental procedure and corticosterone levels after control or non-stressful procedure (NS) (n = 5 mice per group). Unpaired *t*-test, p > 0.05.

(**B**) Percentage and time of NREM and REM sleep after control or non-stressful procedure (NS) (n = 6 mice per group). Unpaired *t*-test, p > 0.05.

(**C**) The experimental procedure and corticosterone levels after control or voluntary wheel running (n = 4 mice per group). Unpaired *t*-test, p > 0.05.

(**D**) Percentage and time of NREM and REM sleep after control or voluntary wheel running (n = 9 mice per group). Unpaired *t*-test, p > 0.05.

(**E**) The experimental procedure and corticosterone levels after control or forced treadmill running (n = 4 mice per group). Unpaired *t*-test, p > 0.05.

(**F**) Percentage and time of NREM and REM sleep after control or forced treadmill running (n = 9 mice per group). Unpaired *t*-test, p > 0.05.

(**G**) The experimental procedure and corticosterone levels after control or novel environment (novelty) (n = 6 mice per group). Unpaired *t*-test, p > 0.05.

(**H**) Percentage and time of NREM and REM sleep after control or novel environment (novelty) (n = 8 mice per group). Unpaired *t*-test, p > 0.05.

(**I-M**) Sleep latency and episode duration of NREM and REM sleep after SDS / control (n = 8 mice per group) (**I**), non-stressful procedure (NS) / control (n = 6 mice per group) (**J**), voluntary wheel running / control (n = 9 mice per group) (**K**), forced treadmill running / control (n = 9 mice per group) (**L**) and novel environment (novelty) / control (n = 8 mice per group) (**M**). Unpaired *t*-test, **p < 0.01, ***p < 0.0001.

Fig. S3 Pharmacologically decreasing corticosterone levels induced by SDS during sleep deprivation does not reduce anxiety

(**A**) Plan of the experimental procedure and corticosterone levels during home cage sleep or sleep deprivation following SDS after vehicle or metyrapone injection (n = 4 mice per group). Two-way ANOVA with bonferroni *post hoc* test, *p < 0.05, **p < 0.01.

(**B**, **C**) Tracing of locomotion for representative animals (**B**), and time spent in the open arms of the elevated plus-maze and in the center zone during the open-field test (**C**) (n = 8 mice per group). Two-way ANOVA with bonferroni *post hoc* test, **p < 0.01, ***p < 0.001, ****p < 0.001.

Fig. S4 Brain-wide mapping of SDS-activated regions (Related to Fig. 1)

(**A**) Immunostaining images showing cFOS-positive cells after control or SDS in diverse brain areas (n = 3 mice per group). Scale bar, 150 μm.

(**B**) Summary of numbers of cFOS-positive cells after control or SDS in diverse brain areas (n = 3 mice per group). PFC, prefrontal cortex; NAc, nucleus acumbens; LS, lateral septum; BNST, bed nucleus stria terminalus; LPO, lateral preoptic area; PVH, paraventricular hypothalamus; LH, lateral hypothalamus; LH, lateral habenula; CeA, central amygdala; PVT, paraventricular thalamus; DMH, dorsal medial hypothalamus; ZI, zona incerta; SuM, supramammilary nucleus; TMN, tuberomamillary nucleus; LC, locus ceruleus; DR, dorsal raphe; PAG, periaquiductal grey.

Fig. S5 Characterization of SDS-activated cells in the VTA (Related to Fig. 1)

(**A**) cFOS expression by immunohistochemistry (green) after control or SDS in genetically labeled VTA^{Vgat} neurons (mCherry expression – *AAV-DIO-mCherry* injected into the VTA of *Vgat-IRES-Cre* mice). Arrowheads indicate double-labeled cells. Scale bar, 100 μm. Schematic diagram summarizing SDS-activated VTA^{Vgat} neurons (mCherry / cFOS) (n = 4 mice).

(**B**) cFOS expression by immunohistochemistry (green) after control or SDS in VTA^{GABA} neurons. Arrowheads indicate double-labeled cells. Schematic diagram summarizing SDS-activated VTA^{GABA} neurons (GABA / cFOS) (n = 4 mice). Quantification represents cFOS and GABA double-labeled cells / total cFOS-positive cells (n = 4 mice for control group; n = 6 mice for SDS group).

(**C**) cFOS expression by immunohistochemistry (green) and quantification after control or SDS in genetically labeled VTA^{Vglut2} neurons (mCherry expression – *AAV-DIO-mCherry* injected into the VTA of *Vglut2-IRES-Cre* mice). Arrowheads indicate double-labeled cells. Scale bar, 50 μm. Diagram summarizing SDS-activated VTA^{Vglut2} neurons (mCherry / cFOS) (n = 4

mice). Quantification represents cFOS and *vglut2* double-labeled cells / total cFOS-positive cells, and cFOS and *vglut2* double-labeled cells / total *vglut2*-positive cells (n = 4 mice per group).

(**D**) cFOS expression and quantification after control or SDS in VTA^{TH} neurons (wild-type mice, immunohistochemistry with tyrosine hydroxylase, TH). Scale bar, 50 μm. Diagram summarizing SDS-activated VTA^{TH} neurons (TH / cFOS) (n = 4 mice). Quantification represents cFOS and TH double-labeled cells / total cFOS-positive cells, and cFOS and TH double-labeled cells / total TH-positive cells (n = 4 mice per group). IF, interfasicular nucleus; RLi, rostral linear nucleus; PBP, parabrachial pigmented nucleus; IPN, interpeduncular nucleus.

(**E**, **F**) cFOS expression by immunohistochemistry (red) after control / voluntary wheel running (**E**) or control / forced treadmill running (**F**) in VTA^{TH} neurons (immunohistochemistry with tyrosine hydroxylase, TH) or genetically labeled VTA^{Vgat} neurons (GFP expression – *AAV-DIO-GFP* injected into the VTA of *Vgat-IRES-Cre* mice).

(**G**) Quantification of total cFOS-positive cells in the VTA, cFOS and TH double-labeled cells / total cFOS-positive cells or cFOS and vgat double-labeled cells / total cFOS-positive cells in control, voluntary wheel running and forced treadmill running group (n = 6 mice per group). Scale bar, 100 μm.

Fig. S6 VTA^{Vgat} neurons do not respond to novel objects, voluntary wheel running, forced treadmill running or novel environments (Related to Fig. 1)

(**A**, **B**) Fiber photometry experiments measuring calcium signals in VTA^{Vgat} neurons when mice were presented with a novel object (**A**) or placed in a novel environment (novelty) (**B**). Raw calcium signal traces, color matrix of GCaMP6 signals for all trials, ΔF/F ratios across the experimental period and average ΔF/F ratios before and during the novel object presentation (n = 6 mice, 17 trials) or novel environment (novelty) (n = 4 mice, 5 trials). Paired two-tailed *t*-test, $p > 0.05$.

(**C, D**) Fiber photometry measuring long-term calcium signals in VTA^{Vgat} neurons. Traces across the experimental procedure and average ΔF/F ratios before and after 1-h voluntary wheel running or 1-h forced treadmill running (**C**), or in a novel environment (novelty) (**D**) for an hour (n = 6 mice per group). Paired two-tailed *t*-test, $p > 0.05$.

Fig. S7 No sleep is induced after chemogenetic reactivation of non-stressful procedure, voluntary wheel running or forced treadmill running tagged VTA^{Vgat} neurons (activity-tagging control of social interaction or physical activity) (Related to Fig. 2)

(**A**) The activity-tagging protocol for testing non-stressful procedure activated VTA^{Vgat} cells for sleep.

(**B**, **C**) Sleep latency, percentage and time of NREM (**B**) or REM (**C**) sleep after chemogenetic reactivation of non-stressful procedure tagged VTA^{Vgat} neurons (n = 6 mice per group). Unpaired *t*-test, $p > 0.05$.

(**D**) The activity-tagging protocol for testing voluntary wheel running activated VTA^{Vgat} cells for sleep and activity-tagged hM3Dq-mCherry transgene in VTA^{Vgat} neurons. Scale bar, 100 μm.

(**E**, **F**) Sleep latency, percentage and time of NREM (**E**) or REM (**F**) sleep after chemogenetic reactivation of voluntary wheel running tagged VTA^{Vgat} neurons (n = 6 mice per group). Unpaired *t*-test, $p > 0.05$.

(**G**) The activity-tagging protocol for testing forced treadmill running activated VTA^{Vgat} cells for sleep and activity-tagged hM3Dq-mCherry transgene in VTA^{Vgat} neurons. Scale bar, 100 μm.

(**H**, **I**) Sleep latency, percentage and time of NREM (**E**) or REM (**F**) sleep after chemogenetic reactivation of forced treadmill running tagged VTA^{Vgat} neurons (n = 6 mice per group). Unpaired *t*-test, $p > 0.05$.

Fig. S8 Fiber photometry shows no changes in calcium signals in VTA^{Vgat} terminals in the CeA, DG or LHb during SDS (Related to Fig. 3)

(**A**-**C**) Fiber photometry measuring terminal activity of VTA^{Vgat} neurons in CeA (**A**), DG (**B**) or LHb (**C**) during SDS. Color matrix of GCaMP6 signals in CeA (**A**), DG (**B**) or LHb (**C**) for all trials, ΔF/F ratios across the experimental period and average ΔF/F ratios before and during the SDS (n = 4 mice per group). Paired *t*-test, $p > 0.05$. Scale bar, 200 μm.

Fig. S9 Collateral tracing of SDS-tagged LH-projecting VTA^{Vgat} cells shows that the LH is their main projection site (Related to Fig. 3)

(**A**) The activity-tagging protocol and expression of the hM3Dq-mCherry transgene in SDS-tagged LH-projecting VTA^{Vgat} cells. Scale bar, 100 μm.

(**B**) Tracing of SDS-tagged LH-projecting VTA^{Vgat} cells in the LH and collateral tracing in other brain regions. Scale bar, 200 μm. LPO, lateral preoptic area; LH, lateral hypothalamus; LHb, lateral habenula; CeA, central amygdala; BLA, basolateral amygdala; DG, dentate gyrus of the hippocampus.

Fig. S10 Optogenetic manipulation of SDS-tagged VTA^{Vgat} neurons (Related to Fig. 3)
(**A**) The activity-tagging protocol for anterograde tracing of activity-tagged VTA^{Vgat} neurons using ChR2-EYFP and the expression of control- or SDS-tagged ChR2-EYFP in VTA^{Vgat} neurons. Scale bar, 200 μm.
(**B**) Projections of non-tagged VTA^{Vgat} neurons (pan expression) in diverse brain areas (*AAV-DIO-ChR2-EYFP* injected into the VTA of *Vgat-IRES-Cre* mice). Scale bar, 100 μm.
(**C**) Projections of control- or SDS-tagged VTA^{Vgat} neurons in diverse brain areas. PFC, prefrontal cortex; MPO, medial preoptic area; LPO, lateral preoptic area; LH, lateral hypothalamus; LHb, lateral habenula; CeA, central amygdala; BLA, basolateral amygdala; DG, dentate gyrus of the hippocampus; 4V, 4th ventricle of the brain. Scale bar, 100 μm.
(**D**) The activity-tagging protocol for opto-reactivation of the tagged VTA^{Vgat} → LH pathway.
(**E**) Opto-reactivation of SDS-tagged VTA^{Vgat} → LH pathway from waking. EMG, EEG, spectra, percentage and time of wakefulness, NREM or REM sleep (n = 6 control-tagged mice; n = 9 SDS-tagged mice). Mann-Whitney test, $^{**}p < 0.01$.

Fig. S11 Presynaptic inputs to VTA^{Vgat} neurons (Related to Fig. 3)
The protocol using rabies virus-based retrograde tracing for identification of presynaptic inputs to VTA^{Vgat} neurons and a schematic color matrix showing the percentage of total inputs from the whole brain to the VTA^{Vgat} neurons as determined by N2CDG rabies retrograde tracing. The heat code indicates the percentage of total inputs as determined by counting the numbers of rabies-labeled presynaptic cells.

Fig. S12 Stress-driven inputs to VTA^{Vgat} neurons from PAG and PVH (Related to Fig. 3)
(**A**) Rabies virus-mediated tracing protocol for identification of stress-driven inputs to VTA^{Vgat} neurons.
(**B**) Immunostaining images, diagram and the number of VTA^{Vgat} starter cells (n = 4 mice) in the VTA and surrounding areas. Scale bar, 200 μm and 50 μm (inset).

(**C**) Immunostaining images showing presynaptic inputs to VTA^{Vgat} neurons from PAG or PVH and cFOS-positive cells activated by SDS. cFOS (green) and mCherry (rabies, red) were stained by immunocytochemistry. Scale bar, 200 μm and 50 μm (inset).

(**D**) Rabies virus-mediated tracing protocol for identification of stress-driven inputs to LH-projecting VTA^{Vgat} neurons.

(**E**) Immunostaining images, diagram and the number of LH-projecting VTA^{Vgat} starter cells in the VTA and surrounding areas (n = 4 mice). Scale bar, 200 μm and 50 μm (inset).

(**F**) Immunostaining images showing presynaptic inputs to LH-projecting VTA^{Vgat} neurons from PAG or PVH and cFOS-positive cells activated by SDS. Scale bar, 200 μm and 50 μm (inset). PVH, paraventricular hypothalamus; PAG, periaquiductal grey; 3V, 3rd ventricle.

Fig. S13 Control inputs to VTA^{Vgat} neurons from LPO, PAG and PVH (Related to Fig. 3)

(**A**) Tracing protocol and immunostaining images showing presynaptic inputs to VTA^{Vgat} neurons from LPO, PAG or PVH and cFOS-positive cells of control. cFOS (green) and mCherry (rabies, red) were stained by immunocytochemistry. Scale bar, 200 µm.

(**B**) Tracing protocol and immunostaining images showing presynaptic inputs to LH-projecting VTA^{Vgat} neurons from LPO, PAG or PVH and cFOS-positive cells of control. Scale bar, 100 mm. LPO, lateral preoptic area; PVH, paraventricular hypothalamus; PAG, periaquiductal grey; 3V, 3rd ventricle of the brain.

Fig. S14 Specificity of stress-driven inputs to SDS-activated VTA^{Vgat} cells (Related to Fig. 3)

(**A**) The work-flow protocol for identifying SDS-driven inputs to VTA^{Vgat} neurons lacking SDS-tagged VTA^{Vgat} cells (activity-tagging-induced expression of caspase).

(**B**) Immunostaining images showing that stress-driven inputs to LH-projecting VTA^{Vgat} neurons identified by rabies labeling (mCherry) disappeared when SDS-tagged VTA^{Vgat} cells have been specifically ablated by caspase (SDS-tagging-induced expression of Caspase, thereby ablation of SDS-activated VTA^{Vgat} cells). In control, images where VTA^{Vgat} neurons were control-experience-tagged with caspase (control experience did not induce caspase expression). Scale bar, 150 µm and 100 µm (inset).

(**C**) Summary of activated fractions in diverse brain areas for all groups shown in **B** (n = 3 mice per group for control-tagged; n = 4 mice per group for SDS-tagged).

PFC, prefrontal cortex; NAc, nucleus acumbens; LS, lateral septum; BNST, bed nucleus of the stria terminalis; LPO, lateral preoptic area; PVH, paraventricular hypothalamus; LH, lateral hypothalamus; LH, lateral habenula; CeA, central amygdala; PVT, paraventricular thalamus; DMH, dorsal medial hypothalamus; ZI, zona incerta; SuM, supramammilary nucleus; TMN, tuberomamillary nucleus; LC, locus ceruleus; DR, dorsal raphe; PAG, periaquiductal grey. 3V, 3rd ventricle.

Fig. S15 Molecular identities of SDS-tagged VTA^{Vgat} neurons and topology of VTA^{Sst} and VTA^{Pv} neurons (Related to Fig. 4)

(**A**) The activity-tagging and single-cell RT-qPCR protocol to profile SDS-tagged VTA^{Vgat} neurons. Scale bar, 25 µm.

(**B**) Heat map and doughnut chart for the single-cell qPCR of patched SDS-tagged VTA^{Vgat} cells (n = 70 cells from 9 mice). The genes tested were: *Slc32a1* (*vgat*); *Sst*; *Pv*; *Vip*; *Slc17a6* (*vglut2*); *Slc6a3* (*dat*).

(**C**) Immunostaining image showing *Sst-Ai9-tdTomato* cells (*Sst-IRES-Cre* X Ai9 reporter mice) in the VTA and diagram summarizing *Sst-Ai9-tdTomato* cells in the VTA (n = 3 mice). Scale bar, 100 μm.

(**D**) Immunostaining image showing *Pv-Ai9-tdTomato* cells (*Pv-Cre* X Ai9 reporter mice) in the VTA and diagram summarizing *Pv-Ai9-tdTomato* cells in the VTA (n = 3 mice). Scale bar, 100 μm.

(**E**) cFOS expression by immunohistochemistry (green) after SDS in VTA$^{Sst\text{-}tdTomato}$ neurons (*Sst-Ai9-tdTomato*). Arrowheads indicate double-labeled cells. Scale bar, 100 μm. Schematic diagram summarizing SDS-activated VTA$^{Sst\text{-}tdTomato}$ neurons (Sst-tdTomato / cFOS) (n = 4 mice). Scale bar, 100 μm.

(**F**) cFOS expression by immunohistochemistry (green) after SDS in VTA$^{Sst\text{-}mCherry}$ neurons (*AAV-DIO-hM3Dq-mCherry* injected into the VTA of *Sst-IRES-Cre* mice). Arrowheads indicate double-labeled cells. Diagram summarizing SDS-activated VTA$^{Sst\text{-}mCherry}$ neurons (Sst-mCherry / cFOS) (n = 4 mice). Quantification represents cFOS and Sst-mCherry double-labeled cells / total Sst-mCherry cells (n = 4 mice per group). Scale bar, 100 μm.

Fig. S16 Molecular identities of SDS-tagged VTA^{Sst} neurons (Related to Fig. 4)

(**A**, **B**) The activity-tagging protocol (**A**), expression of the hM3Dq-mCherry transgene in pan (*AAV-DIO-mCherry* injected into the VTA of *Sst-IRES-Cre* mice) or SDS-tagged VTA^{Sst} neurons (n = 5 mice per group) (**B**). Scale bar, 100 μm.

(**C**) Single-cell RT-qPCR protocol to profile SDS-tagged VTA^{Sst} neurons. Scale bar, 25 μm.

(**D**) Heat map and doughnut chart for the single-cell qPCR of patched SDS-tagged VTA^{Sst} cells (n = 48 cells from 6 mice). The genes tested were: *Slc32a1* (*vgat*); *Gad1*; *Slc17a6* (*vglut2*); *Slc6a3* (*dat*).

Fig. S17 VTA^{Sst} neurons do not respond to restraint or cage-change stress (Related to Fig. 4)

(**A**, **B**) Fiber photometry measuring calcium signals in VTA^{Sst} neurons responding to restraint (n = 9 mice per group, 13 trials) (**A**) or cage-change (n = 6 mice per group, 9 trials) (**B**). Raw calcium signal traces, color matrix of GCaMP6 signals for all trials, ΔF/F ratios across the

experimental period and average ΔF/F ratios before and during the procedures. Paired *t*-test, $p > 0.05$.

(**C**, **D**) Fiber photometry measuring long-term calcium signals in VTA^{Sst} neurons (n = 8 per group) in response to restraint (**C**) or cage-change stress (**D**). Raw traces and average ΔF/F ratios before and after procedures. Paired *t*-test, $p > 0.05$.

(**E**) The activity-tagging protocol, expression and quantification of restraint- or cage-change-tagged hM3Dq-mCherry transgene expression in VTA^{Sst} neurons (n = 4 mice per group).

Fig. S18 Chemogenetic activation of VTA^{Sst} neurons promotes NREM and REM sleep (Related to Fig. 5)

(**A**, **B**) Percentage and time of NREM (**A**) or REM (**B**) sleep after chemogenetic activation of VTA^{Sst} neurons (n = 6 mice per group). Unpaired *t*-test, $^{**}p < 0.01$, $^{****}p < 0.0001$.

Fig. S19 SDS-tagged VTA^{Sst} neurons are NREM and REM sleep-active and activation of SDS-tagged VTA^{Sst} neurons promotes NREM and REM sleep (Related to Fig. 5)

(**A**) The activity-tagging protocol for fiber photometry with EEG/EMG measuring the spontaneous activity of SDS-tagged VTA^{Sst} neurons across brain states.

(**B**) ΔF/F ratio of the Ca^{2+} signal in SDS-tagged VTA^{Sst} neurons during wakefulness, NREM and REM sleep (n = 6 mice per group). Photometry signals in SDS-tagged VTA^{Sst} neurons at transitions of vigilance states (n = 6 mice per group). One-way repeated ANOVA, $^{*}p < 0.05$, $^{***}p < 0.001$.

(**C**) The activity-tagging protocol for testing the sufficiency of SDS-tagged VTA^{Sst} cells for sleep.

(**D**, **E**) Sleep latency, percentage and time of NREM (**D**) or REM (**E**) sleep after chemogenetic reactivation of SDS-tagged VTA^{Sst} neurons (n = 6 mice per group). Unpaired *t*-test, $^{*}p < 0.05$, $^{**}p < 0.01$, $^{****}p < 0.0001$.

Fig. S20 VTA^{Sst} neurons mediate SDS-induced sleep via the LH (Related to Fig. 5)

(**A**, **B**) Fiber photometry measuring the terminal calcium signals of the VTA^{Sst}→LH pathway during SDS. The image shows GCaMP6 fiber in the LH (**A**). Raw traces, color matrix of GCaMP6 signals of VTA^{Sst}→LH pathway for all trials, ΔF/F ratios across the experimental period, and average ΔF/F before and during the SDS (**B**) (n = 8 mice, 17 trials). Paired *t*-test, $^{**}p < 0.01$. Scale bar, 100 μm.

(**C**) The intersectional activity-tagging protocol for chemogenetic reactivation of SDS-tagged LH-projecting VTA^{Sst} cells.

(**D**, **E**) Sleep latency, percentage and time of NREM (**D**) and REM (**E**) sleep after chemogenetic reactivation of SDS-tagged LH-projecting VTA^{Sst} cells (n = 6 mice per group). Unpaired *t*-test, *p < 0.05, ****p < 0.0001.

Fig. S21 Efficacy and specificity of genetic ablation (Related to Fig. 5)

(**A**, **B**) Genetic ablation of VTA^{Sst} (**A**) or VTA^{Pv} (**B**) neurons. Image showing GFP expression in *AAV-DIO-GFP* injected controls or *AAV-DIO-GFP* and *AAV-DIO-Casp3-TEV* injected group. Scale bar, 100 μm.

(**C**, **D**) Immunostaining images (**C**) and quantifications (**D**) showing somatostatin (SST)-positive cells in the VTA and surrounding areas in control or caspase mice (n = 5 mice per group). Scale bar, 100 μm. RN, red nucleus; IPN, interpeduncular nucleus; SuM, supramammillary nucleus; RMTg, rostromedial tegmental nucleus; SNr, substantia nigra. Unpaired *t*-test, ***p < 0.001.

Fig. S22 VTA^{Sst} neurons are necessary for SDS-induced sleep (Related to Fig. 5)

(**A**) Chemogenetic inhibition for testing the necessity of VTA^{Sst} cells for SDS-induced sleep.

(**B**, **C**) Percentage and time of NREM (**B**) or REM sleep (**C**) in mice given SDS after chemogenetic inhibition of VTA^{Sst} cells (n = 6 mice per group). Two-way ANOVA with bonferroni *post hoc* test. **p<0.01, ***p<0.001, n.s: not significant.

Fig. S23 Lesioning of VTA^{Sst} neurons or inhibition of SDS-tagged VTA^{Vgat} neurons does not affect baseline anxiety levels in mice (Related to Fig. 6)

(**A**) Plan of the experimental procedure. Behavioral tests (EPM and OFT) were performed as arrowhead indicates.

(**B**, **C**) Tracing of locomotion for representative animals (**B**), time spent in the open arms of the elevated plus-maze and in the center zone during the open-field test of GFP mice and caspase mice (n = 9 mice per group) (**C**). Unpaired *t*-test, p > 0.05.

(**D**) Plan of the experimental procedure and the activity-tagging protocol for chemogenetic manipulation of SDS-activated VTA^{Vgat} cells. Behavioral tests (EPM and OFT) were performed as arrowhead indicates.

(**E**, **F**) Tracing of locomotion for representative animals (**E**), time spent in the open arms of the elevated plus-maze and in the center zone during the open-field test of saline- and CNO-injected mice (n = 6 mice per group) (**F**). Unpaired *t*-test, p > 0.05.

Fig. S24 SDS-induced sleep by VTA^{Sst} neurons and SDS-tagged VTA^{Vgat} neurons reduces SDS-induced anxiety (Related to Fig. 6)

(**A**) Plan of the experimental procedure for chemogenetic inhibition of VTA^{Sst} cells followed by control or SDS for behavioral tests, as arrowheads indicate.

(**B**, **C**) Tracing of locomotion for representative animals (**B**), time spent in the open arms of the elevated plus-maze and in the center zone during the open-field test of saline- or CNO-injected mice (n = 8 mice per group) (**C**). Two-way ANOVA with Bonferroni *post hoc* test, *p < 0.05, **p < 0.01, ***p < 0.001.

(**D**) Plan of the experimental procedure and the activity-tagging protocol for chemogenetic manipulation of SDS-activated VTA^{Vgat} cells (1st SDS-tagging) followed by control or SDS (2nd SDS) for behavioral tests, as arrowheads indicate.

(**E**, **F**) Tracing of locomotion for representative animals (**E**), time spent in the open arms of the elevated plus-maze and in the center zone during the open-field test of saline- or CNO-injected mice (**F**) (n = 6 mice per group). Two-way ANOVA with Bonferroni *post hoc* test, **p < 0.01, ***p < 0.001, ****p < 0.0001.

Fig. S25 Lacking SDS-induced sleep by lesioning of VTA^{Sst} neurons does not reduce SDS-induced anxiety, and is similar to the effect of sleep deprivation (Related to Fig. 6)

(**A**) Plan of the experimental procedure. Behavioral tests were performed as arrowhead indicates.

(**B**, **C**) Tracing of locomotion for representative animals (**B**), time spent in the open arms of the elevated plus-maze and in the center zone during the open-field test of GFP and caspase mice after control or SDS followed by 4-h home cage sleep or sleep deprivation (**C**) (n = 9 mice per group). Two-way ANOVA with Bonferroni *post hoc* test, **p < 0.01, ****p < 0.0001.

Fig. S26 Sleep deprivation suppresses the activity in VTA^{Sst} neurons induced by SDS (Related to Fig. 6)

Fiber photometry measuring long-term calcium signals in VTA^{Sst} neurons in response to SDS followed by sleep deprivation or home cage sleep. Raw traces (**A**) and average ΔF/F ratios (**B**)

during sleep deprivation or home cage sleep after SDS (n = 8 mice per group). Unpaired *t*-test, ***$p < 0.001$.

Fig. S27 Activation of VTA^{Sst} neurons during sleep deprivation (in the absence of sleep) after SDS does not reduce anxiety

(**A**) Plan of the experimental procedure for chemogenetic activation of VTA^{Sst} neurons during sleep deprivation after SDS for behavioral tests, as arrowhead indicate.

(**B**) cFOS expression and cFOS expression in VTA^{Sst} neurons (hM3Dq-mCherry) 3 hours after saline or CNO injection during sleep deprivation after SDS. Scale bar, 100 µm (left) and 25 µm (right).

(**C**, **D**) Tracing of locomotion for representative animals (**B**) and time spent in the open arms of the elevated plus-maze and in the center zone during the open-field test (**C**) (n = 8 mice per group). Two-way ANOVA with bonferroni *post hoc* test, $p > 0.05$, n.s, not significant.

Fig. S28 Innervations of VTA^{Sst} neurons and PVN (Related to Fig. 6)

(**A**, **B**) mCherry-positive terminals in the PVN region from VTA^{Sst} neurons (**A**) with *AAV-DIO-hM3Dq-mCherry* injection into the VTA of *Sst-IRES-Cre* mice (**B**). Scale bar, 200 µm.

(**C**) cFOS expression and quantification in the PVN region following SDS after chemogenetic activation of VTA^{Sst} neurons (n = 4 mice per group). Scale bar, 100 µm.

(**D**) Expression of $GRAB_{CRF1.0}$ sensor in the surrounding area of PVN after delivery of *AAV-hSyn-CRF1.0* into the PVN area of *Sst-IRES-Cre* mice. Scale bar, 200 µm.

(**E**) Plan of the experimental procedure for chemogenetic activation of VTA^{Sst} neurons for measuring CRF levels in the surrounding area of PVN. Raw traces and ΔF/F ratios after saline or CNO injection (n = 8 mice per group). Unpaired *t*-test, $p > 0.05$.

(**F**) Corticosterone levels after chemogenetic activation of VTA^{Sst} neurons (n = 6 mice per group). Two-way ANOVA with Bonferroni *post hoc* test, $p > 0.05$.

(**G**) Plan of the experimental procedure for measuring CRF levels around PVN area in response to SDS. Raw traces and ΔF/F ratios before or during SDS (n = 8 mice per group). Paired *t*-test, **$p < 0.01$.

Fig. S29 SDS-induced sleep by VTA^{Sst} neurons aids alleviating corticosterone levels after SDS (Related to Fig. 6)

(**A**) Plan of the experimental procedure for chemogenetic inhibition of VTA^{Sst} neurons followed by control or SDS for measuring corticosterone levels.

(**B**) Corticosterone levels of saline- or CNO-injected mice during home cage sleep after control or SDS (n = 4-6 mice per group). Two-way ANOVA with Bonferroni *post hoc* test, *p < 0.05, **p < 0.01.

(**C**) Plan of the experimental procedure for chemogenetic activation of VTA^{Sst} neurons during sleep deprivation (in the absence of sleep) after SDS for measuring corticosterone levels.

(**D**) Corticosterone levels of saline- or CNO-injected mice during home cage sleep or sleep deprivation after SDS (n = 6 mice per group). Two-way ANOVA with Bonferroni *post hoc* test, *p < 0.05, **p < 0.01, ***p < 0.001.

Fig. S30 Chemogenetic inhibition of LH^{Hcrt} neurons does not reduce SDS-induced anxiety (Related to Fig. 6)

(**A**) Plan of the experimental procedure for chemogenetic inhibition of LH^{Hcrt} cells followed by control or SDS for behavioral tests, as arrowheads indicate.

(**B**, **C**) Tracing of locomotion for representative animals (**B**), time spent in the open arms of the elevated plus-maze and in the center zone during the open-field test of saline- or CNO-injected mice (n = 8 mice per group) (**C**). Two-way ANOVA with Bonferroni *post hoc* test, *p < 0.05, **p < 0.01.

Fig. S31 Blocking orexin/hcrt receptors does not restore the anxiolytic effects on VTA^{Sst} lesioned mice (Related to Fig. 6)

(**A**) Plan of the experimental procedure for infusion of (*i.c.v*) orexin receptor antagonist (SB-334867 or TCS-OX-29) into the VTA^{Sst} lesioned mice followed by SDS for behavioral tests, as arrowhead indicate.

(**B**, **C**) Tracing of locomotion for representative animals (**B**), time spent in the open arms of the elevated plus-maze and in the center zone during the open-field test (**C**) (n = 6 mice per group). Two-way ANOVA with bonferroni *post hoc* test, *p < 0.05, n.s, not significant.

(**D**) Plan of the experimental procedure for injection of (*i.p*) dual orexin receptor antagonist MK6096 (filorexant) into the VTA^{Sst} lesioned mice followed by SDS for behavioral tests, as arrowhead indicate.

(**E**, **F**) Tracing of locomotion for representative animals (**E**), time spent in the open arms of the elevated plus-maze and in the center zone during the open-field test (**F**) (n = 7 mice per group). Unpaired *t*-test, p > 0.05, n.s, not significant.

Fig. S32 Specificity of the activity-tagging system for SDS (Related to Fig. 2 and Fig. 3)

(**A**, **B**) Testing the efficacy and specificity of the activity-tagging system. The activity-tagging transgenes were delivered into the VTA (**A**) or intersectionally into the LH and VTA (**B**) of *Vgat-IRES-Cre* mice. Expression and quantification of activity-tagged hM3Dq-mCherry transgenes in VTA^{Vgat} neurons under different activity-tagging conditions (n = 4 mice per group). Scale bar, 100 μm.

Fig. S33 Control experiments for GCaMP6 photometry of VTA^{Vgat} neurons (Related to Fig. 1, Fig. 2 and Fig. 3)

(**A**) Fiber photometry set up and control experiments measuring baseline fluorescence and controlling for movement artifacts in VTA^{Vgat}-GFP (*AAV-DIO-GFP* injected into the VTA of *Vgat-IRES-Cre* mouse) neurons when the mouse underwent SDS.

(**B**) Raw calcium signal traces, a color matrix of GCaMP6 signals for all trials, the ΔF/F ratios across the experimental period and average ΔF/F ratios before and during the SDS (n = 4 mice). Paired *t*-test, $p > 0.05$.

33. L. Vong *et al.*, Leptin action on GABAergic neurons prevents obesity and reduces inhibitory tone to POMC neurons. *Neuron* **71**, 142-154 (2011).
34. H. Taniguchi *et al.*, A resource of Cre driver lines for genetic targeting of GABAergic neurons in cerebral cortex. *Neuron* **71**, 995-1013 (2011).
35. S. Hippenmeyer *et al.*, A developmental switch in the response of DRG neurons to ETS transcription factor signaling. *PLoS Biol* **3**, e159 (2005).
36. L. Madisen *et al.*, A robust and high-throughput Cre reporting and characterization system for the whole mouse brain. *Nat Neurosci* **13**, 133-140 (2010).
37. W. J. Giardino *et al.*, Parallel circuits from the bed nuclei of stria terminalis to the lateral hypothalamus drive opposing emotional states. *Nature Neuroscience* **21**, 1084-1095 (2018).
38. S. Ren *et al.*, The paraventricular thalamus is a critical thalamic area for wakefulness. *Science* **362**, 429-434 (2018).
39. Z. Zhang *et al.*, Neuronal ensembles sufficient for recovery sleep and the sedative actions of α2 adrenergic agonists. *Nature Neuroscience* **18**, 553-561 (2015).
40. E. C. Harding *et al.*, A Neuronal Hub Binding Sleep Initiation and Body Cooling in Response to a Warm External Stimulus. *Current Biology* **28**, 2263-2273.e2264 (2018).
41. M. J. Krashes *et al.*, Rapid, reversible activation of AgRP neurons drives feeding behavior in mice. *J Clin Invest* **121**, 1424-1428 (2011).
42. X. Yu *et al.*, GABA and glutamate neurons in the VTA regulate sleep and wakefulness. *Nat Neurosci* **22**, 106-119 (2019).
43. T. W. Chen *et al.*, Ultrasensitive fluorescent proteins for imaging neuronal activity. *Nature* **499**, 295-300 (2013).
44. K. T. Beier *et al.*, Rabies screen reveals GPe control of cocaine-triggered plasticity. *Nature* **549**, 345-350 (2017).
45. C. F. Yang *et al.*, Sexually dimorphic neurons in the ventromedial hypothalamus govern mating in both sexes and aggression in males. *Cell* **153**, 896-909 (2013).
46. A. J. Murray *et al.*, Parvalbumin-positive CA1 interneurons are required for spatial working but not for reference memory. *Nat Neurosci* **14**, 297-299 (2011).
47. D. G. Tervo *et al.*, A Designer AAV Variant Permits Efficient Retrograde Access to Projection Neurons. *Neuron* **92**, 372-382 (2016).
48. R. Lin *et al.*, Cell-type-specific and projection-specific brain-wide reconstruction of single neurons. *Nat Methods* **15**, 1033-1036 (2018).
49. T. R. Reardon *et al.*, Rabies Virus CVS-N2cΔG Strain Enhances Retrograde Synaptic Transfer and Neuronal Viability. *Neuron* **89**, 711-724 (2016).
50. S. Fujii, M. K. Kaushik, X. Zhou, M. Korkutata, M. Lazarus, Acute Social Defeat Stress Increases Sleep in Mice. *Front Neurosci* **13**, 322 (2019).
51. S. A. Golden, H. E. Covington, 3rd, O. Berton, S. J. Russo, A standardized protocol for repeated social defeat stress in mice. *Nat Protoc* **6**, 1183-1191 (2011).
52. M. K. Kaushik, K. Aritake, A. Takeuchi, M. Yanagisawa, Y. Urade, Octacosanol restores stress-affected sleep in mice by alleviating stress. *Scientific Reports* **7**, 8892 (2017).
53. N. Charoenphandhu *et al.*, Mild-intensity physical activity prevents cardiac and osseous iron deposition without affecting bone mechanical property or porosity in thalassemic mice. *Scientific Reports* **12**, 5959 (2022).
54. G.-W. Zhang *et al.*, Medial preoptic area antagonistically mediates stress-induced anxiety and parental behavior. *Nature Neuroscience* **24**, 516-528 (2021).

55. X. Yu *et al.*, Dysfunction of ventral tegmental area GABA neurons causes mania-like behavior. *Molecular Psychiatry* **26**, 5213-5228 (2021).
56. K. Tossell *et al.*, Sleep deprivation triggers somatostatin neurons in prefrontal cortex to initiate nesting and sleep via the preoptic and lateral hypothalamus. *bioRxiv*, 2020.2007.2001.179671 (2020).
57. L. A. Gunaydin *et al.*, Natural neural projection dynamics underlying social behavior. *Cell* **157**, 1535-1551 (2014).
58. G. M. Alexander *et al.*, Remote control of neuronal activity in transgenic mice expressing evolved G protein-coupled receptors. *Neuron* **63**, 27-39 (2009).
59. E. S. Boyden, F. Zhang, E. Bamberg, G. Nagel, K. Deisseroth, Millisecond-timescale, genetically targeted optical control of neural activity. *Nat Neurosci* **8**, 1263-1268 (2005).
60. V. N. Anisimov *et al.*, Reconstruction of vocal interactions in a group of small songbirds. *Nature Methods* **11**, 1135-1137 (2014).
61. P. Mitra, H. Bokil, Observed Brain Dynamics, Oxford Univesrity Press, New York. (2008).
62. C. Tronche *et al.*, Increased stress-induced intra-hippocampus corticosterone rise associated with memory impairments in middle-aged mice. *Neurobiology of Learning and Memory* **93**, 343-351 (2010).
63. R. M. Sears *et al.*, Orexin/hypocretin system modulates amygdala-dependent threat learning through the locus coeruleus. *Proc Natl Acad Sci U S A* **110**, 20260-20265 (2013).
