# Supplementary figures and images for: A specific circuit in the midbrain detects stress and induces restorative sleep
Source: Science. Author manuscript; Available in PMC 2022 Jul 2. (PMC7612951; doi:10.1126/science.abn0853)

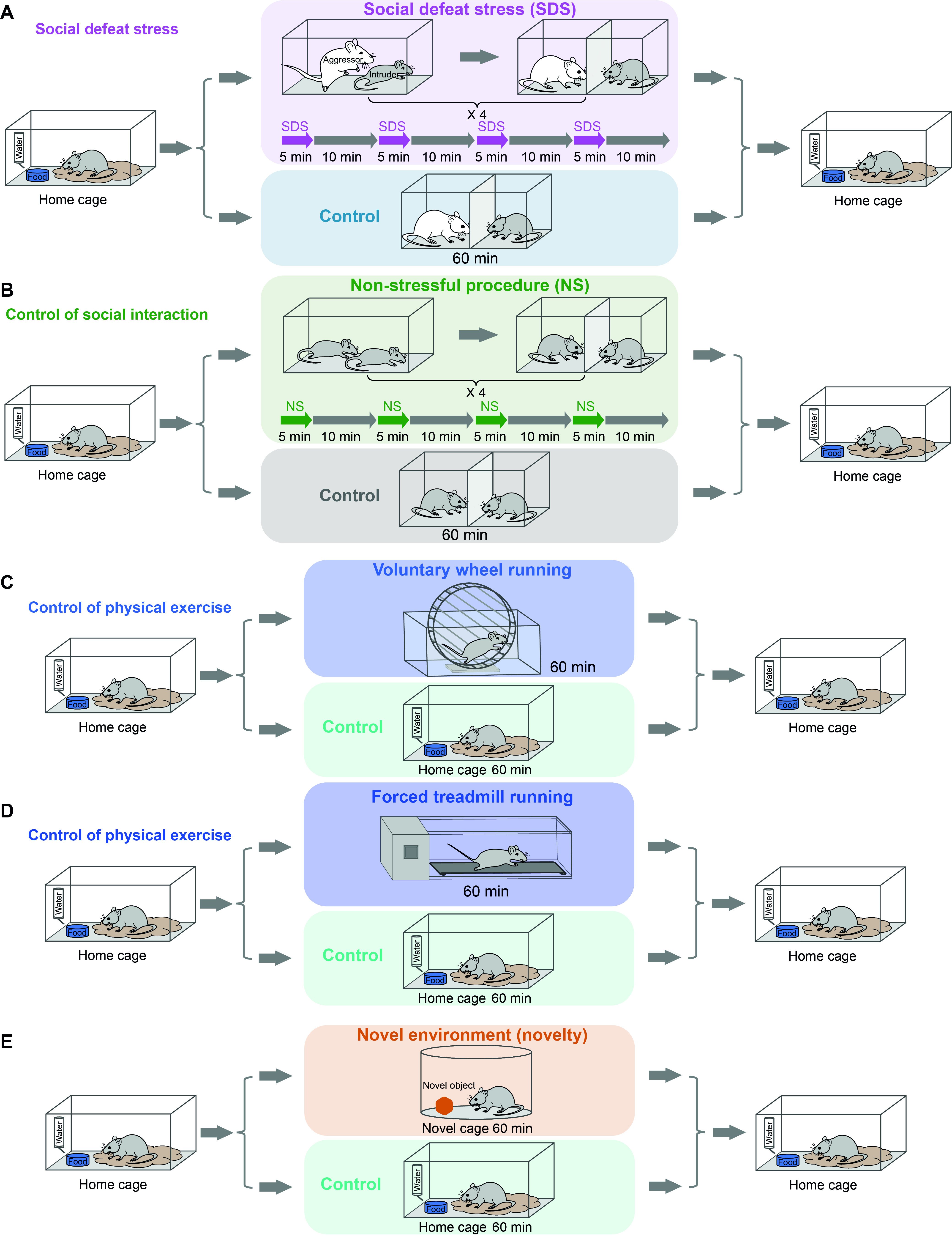

Supplement: Supp. Fig S1 [file EMS145530-supplement-Supp__Fig_S1.jpg]

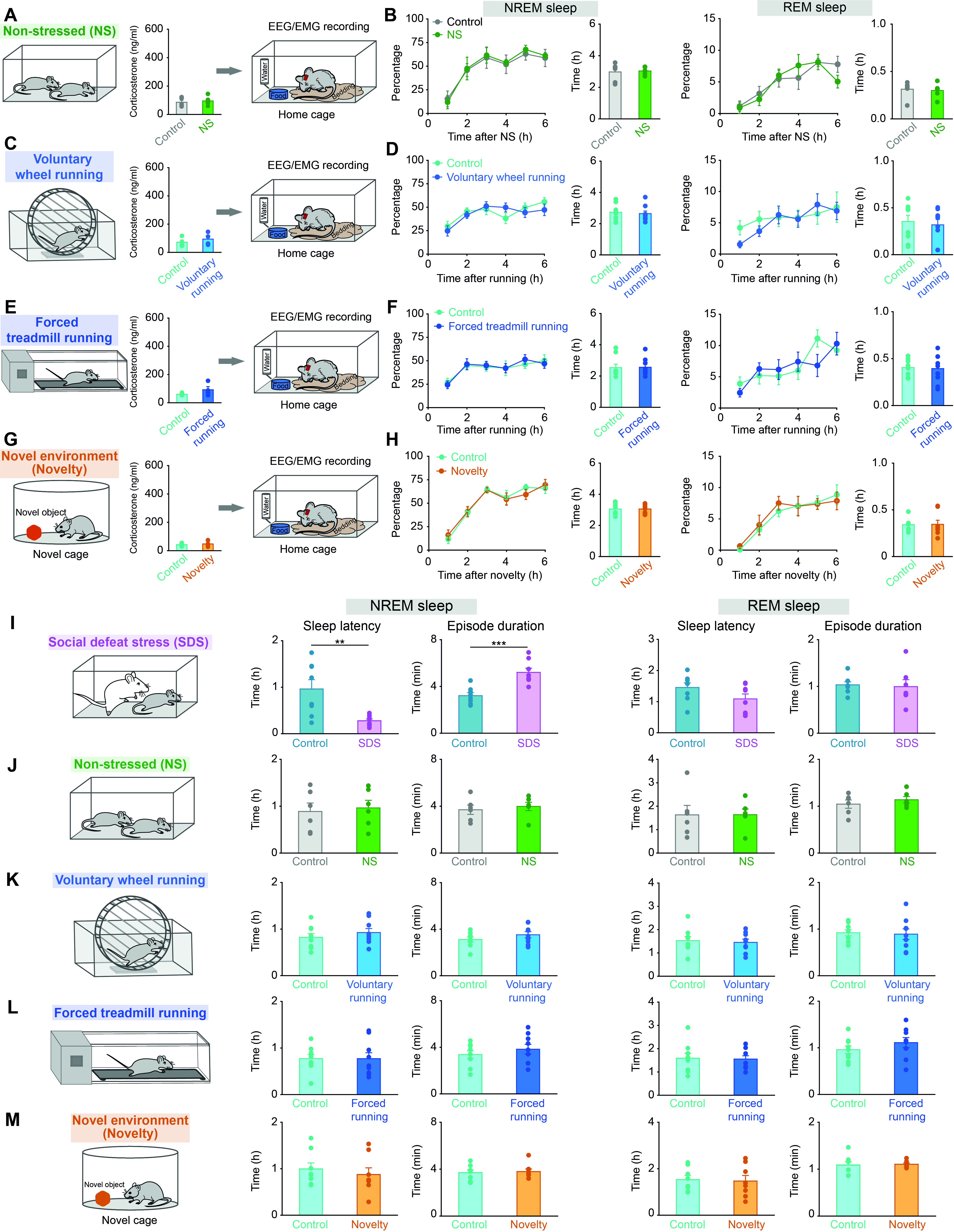

Supplement: Supp. Fig S2 [file EMS145530-supplement-Supp__Fig_S2.jpg]

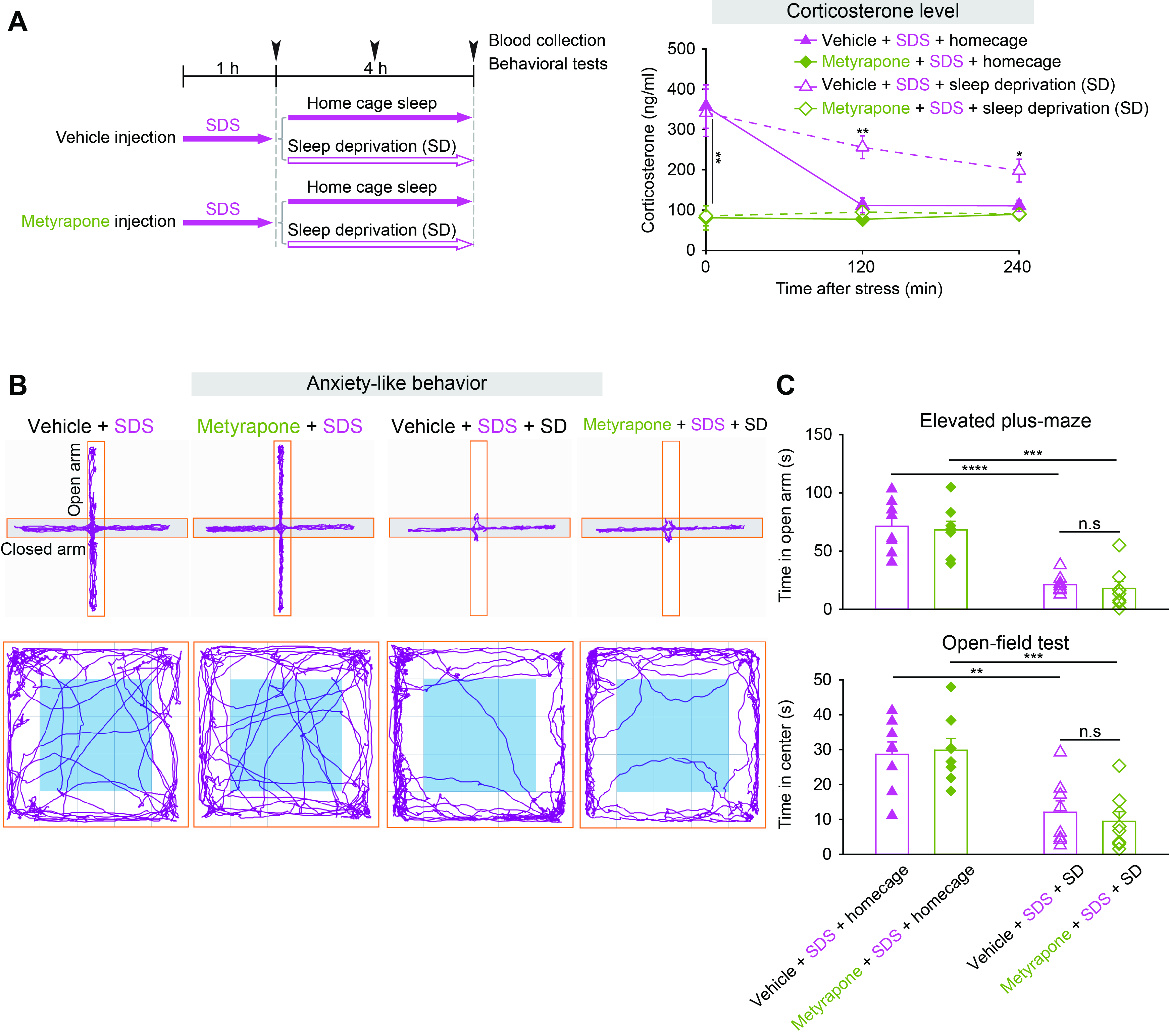

Supplement: Supp. Fig. S3 [file EMS145530-supplement-Supp__Fig__S3.jpg]

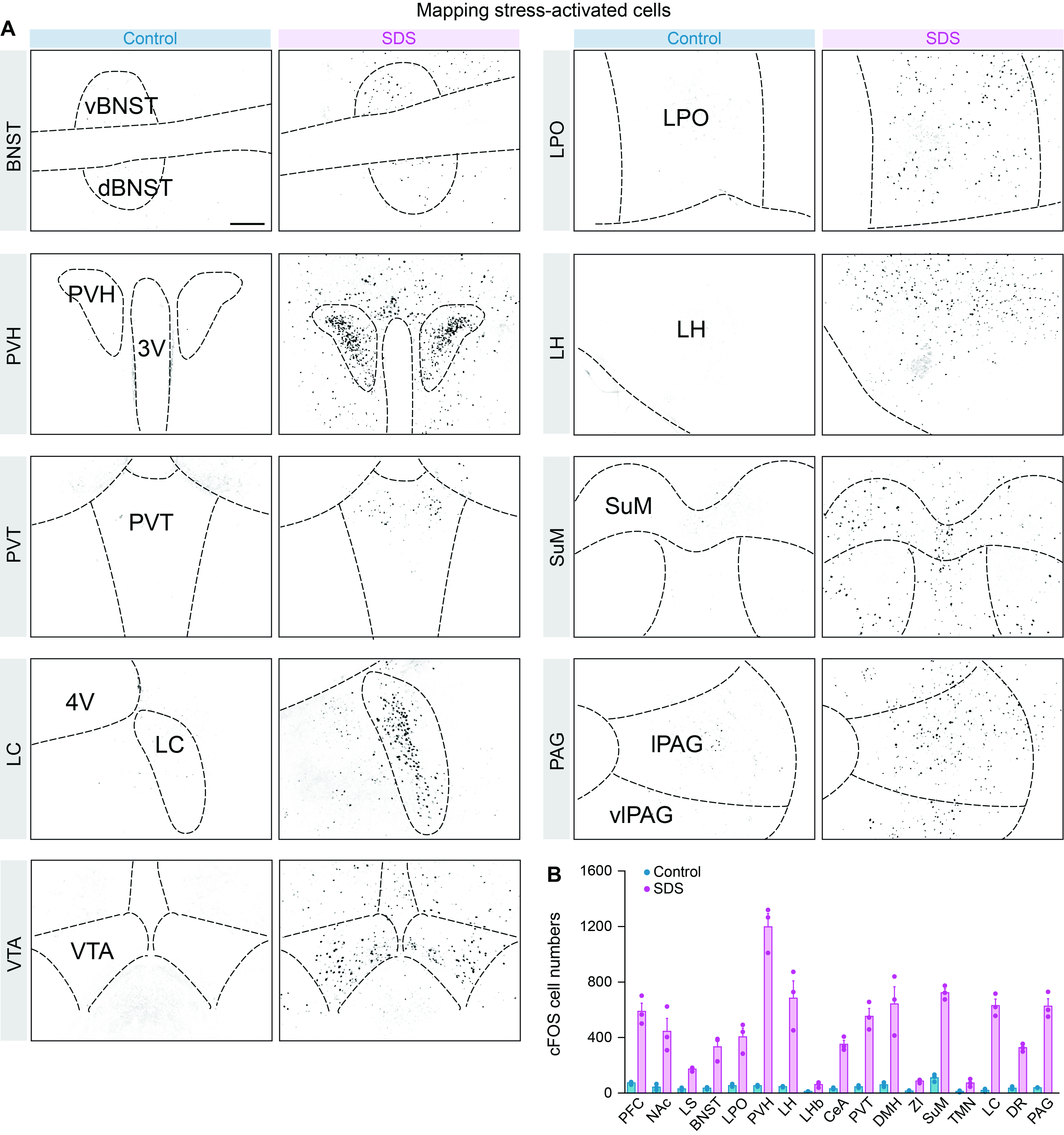

Supplement: Supp. Fig. S4 [file EMS145530-supplement-Supp__Fig__S4.jpg]

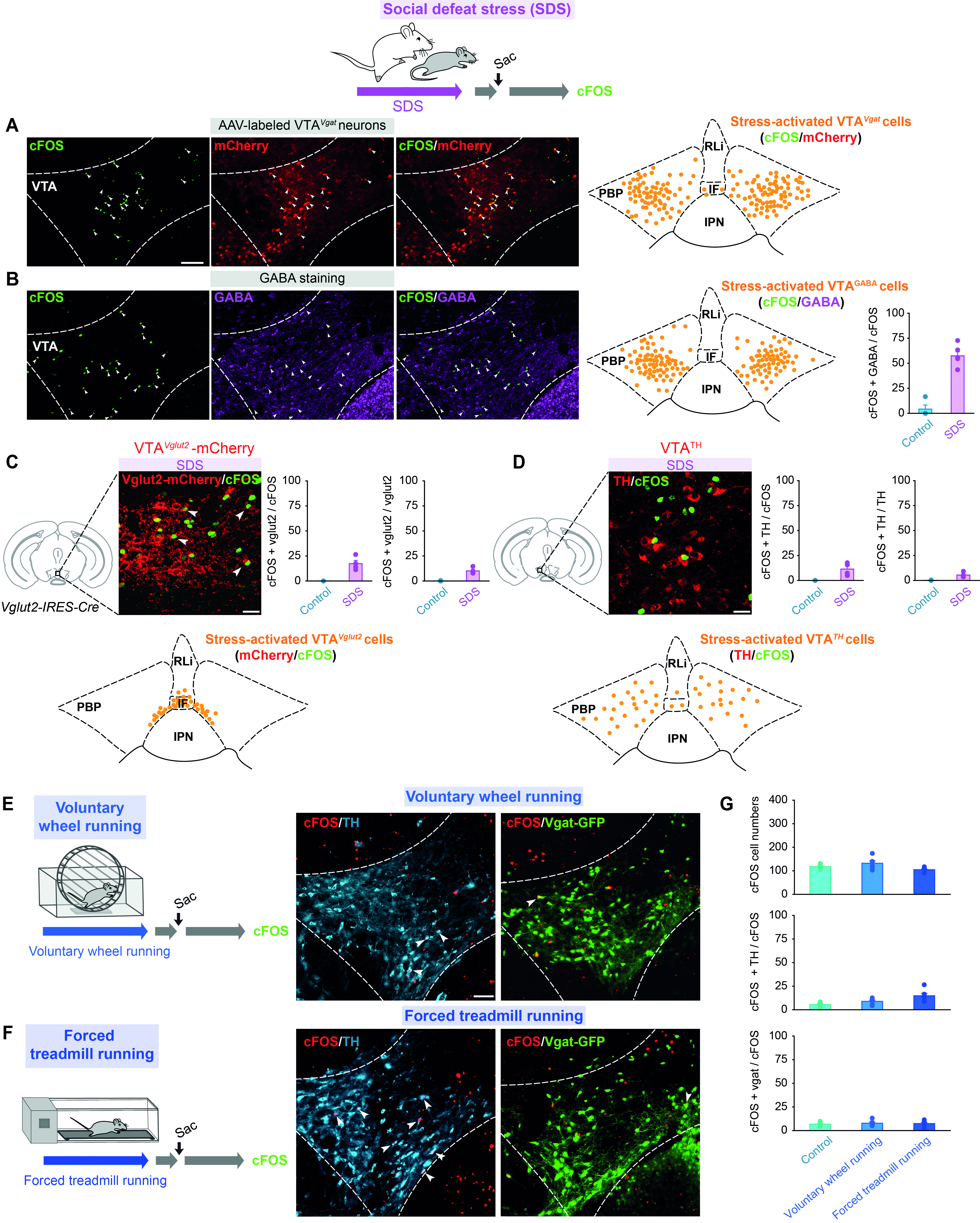

Supplement: Supp. Fig. S5 [file EMS145530-supplement-Supp__Fig__S5.jpg]

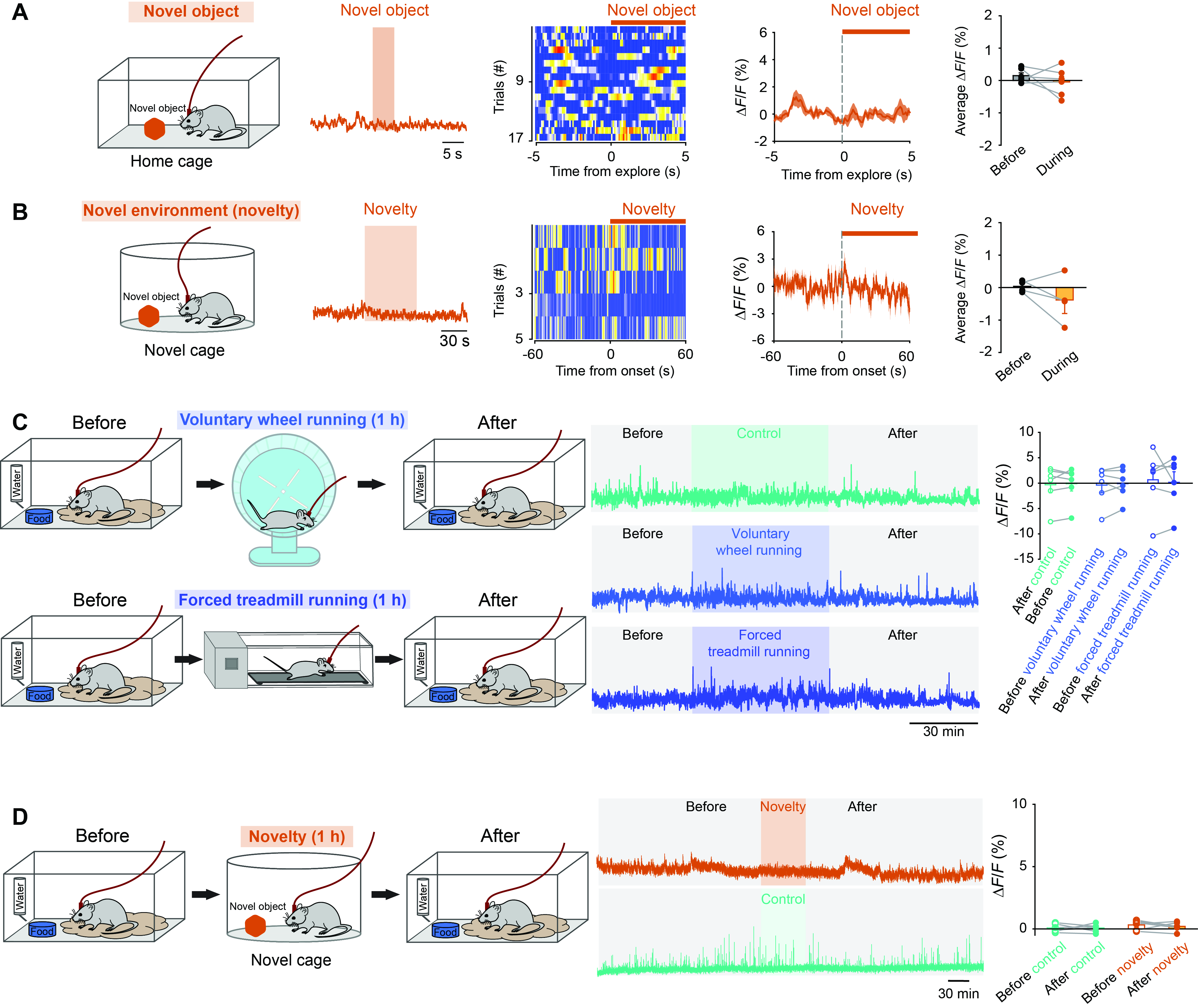

Supplement: Supp. Fig. S6 [file EMS145530-supplement-Supp__Fig__S6.jpg]

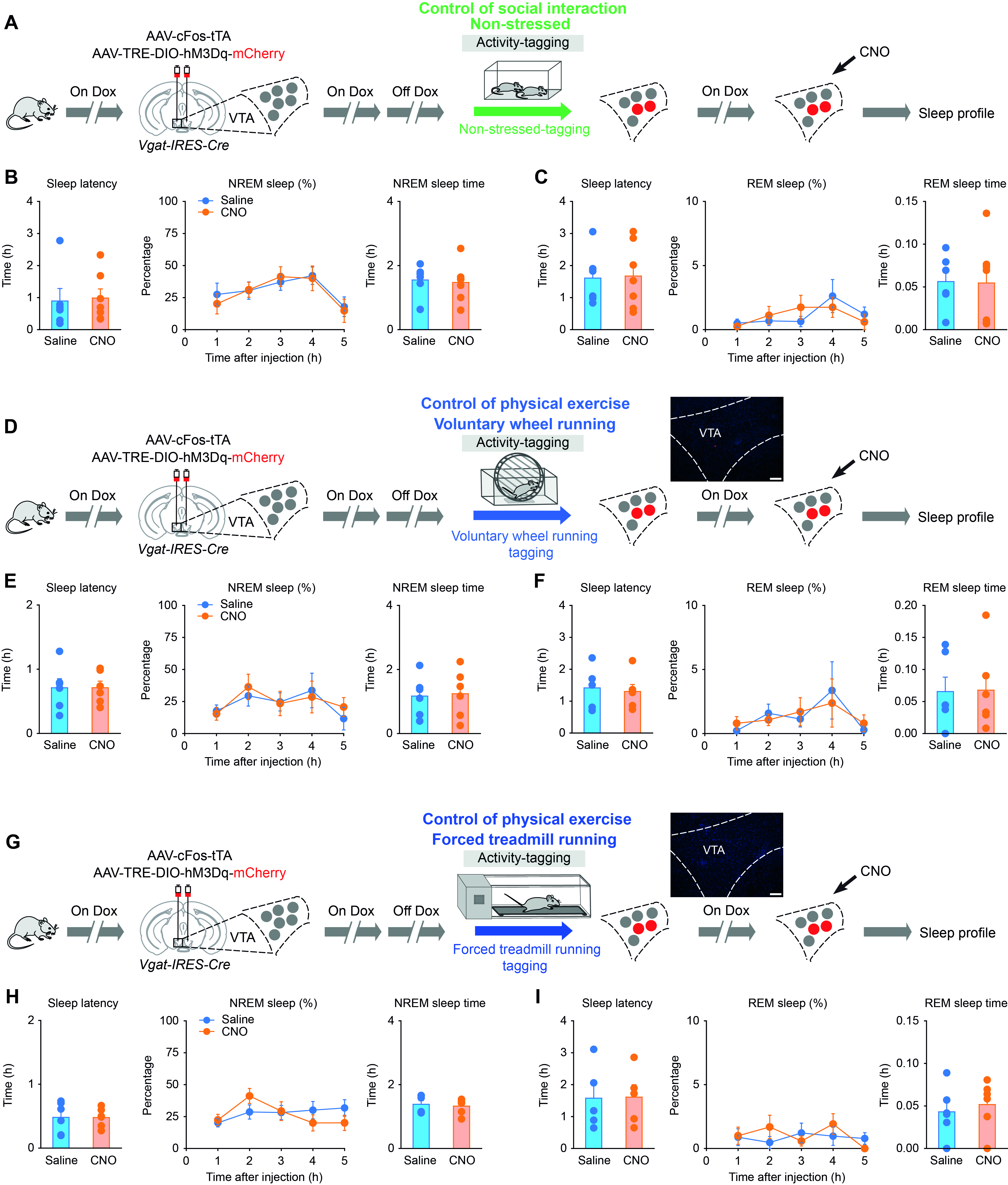

Supplement: Supp. Fig. S7 [file EMS145530-supplement-Supp__Fig__S7.jpg]

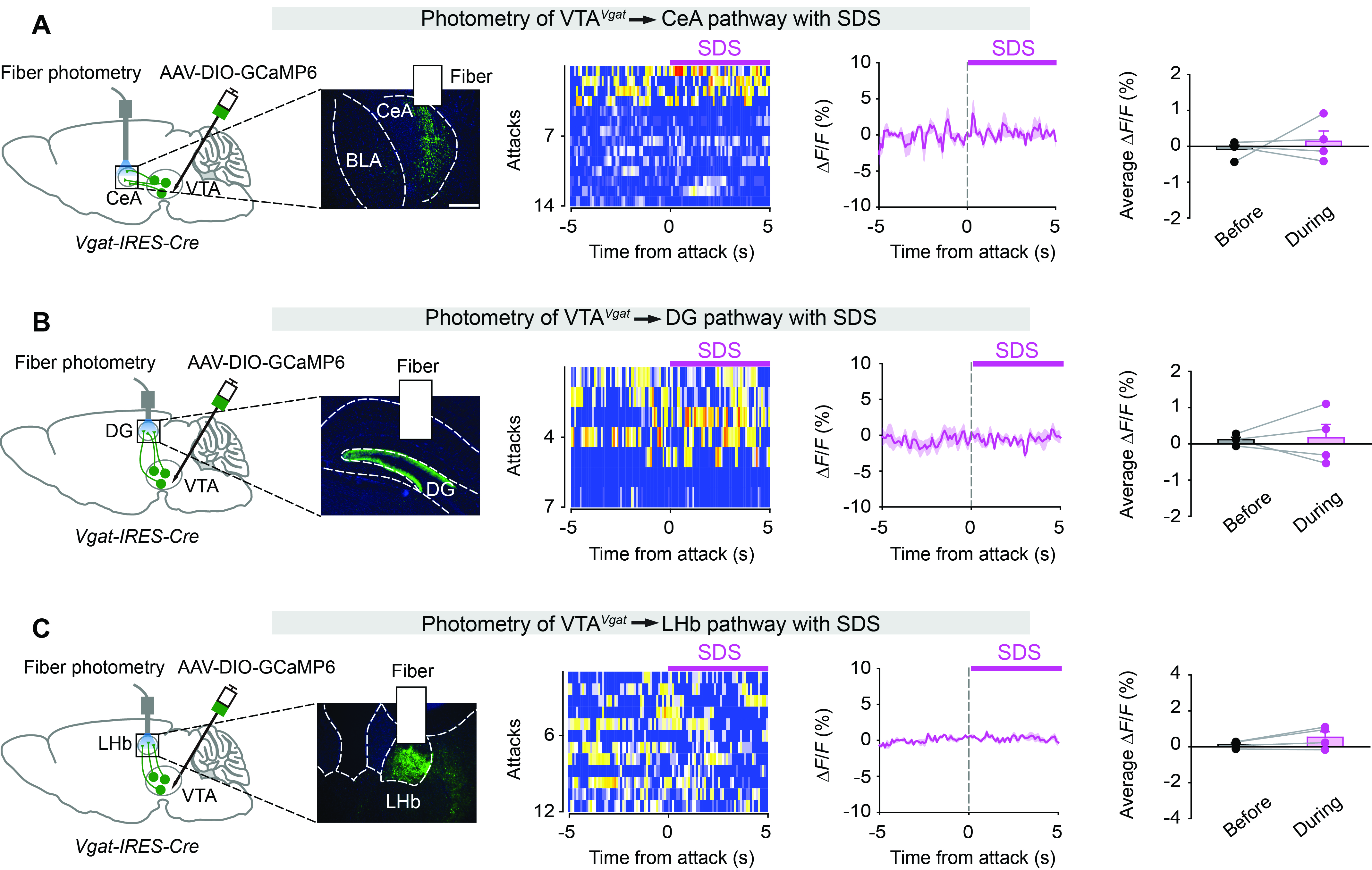

Supplement: Supp. Fig. S8 [file EMS145530-supplement-Supp__Fig__S8.jpg]

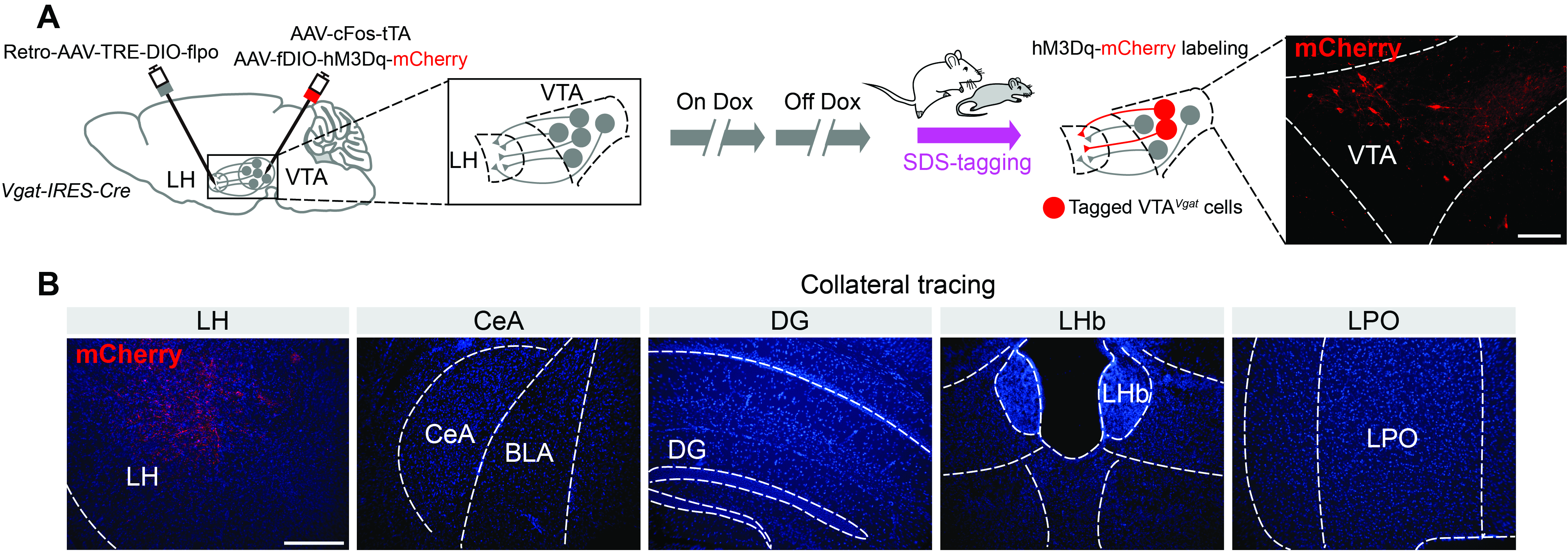

Supplement: Supp. Fig. S9 [file EMS145530-supplement-Supp__Fig__S9.jpg]

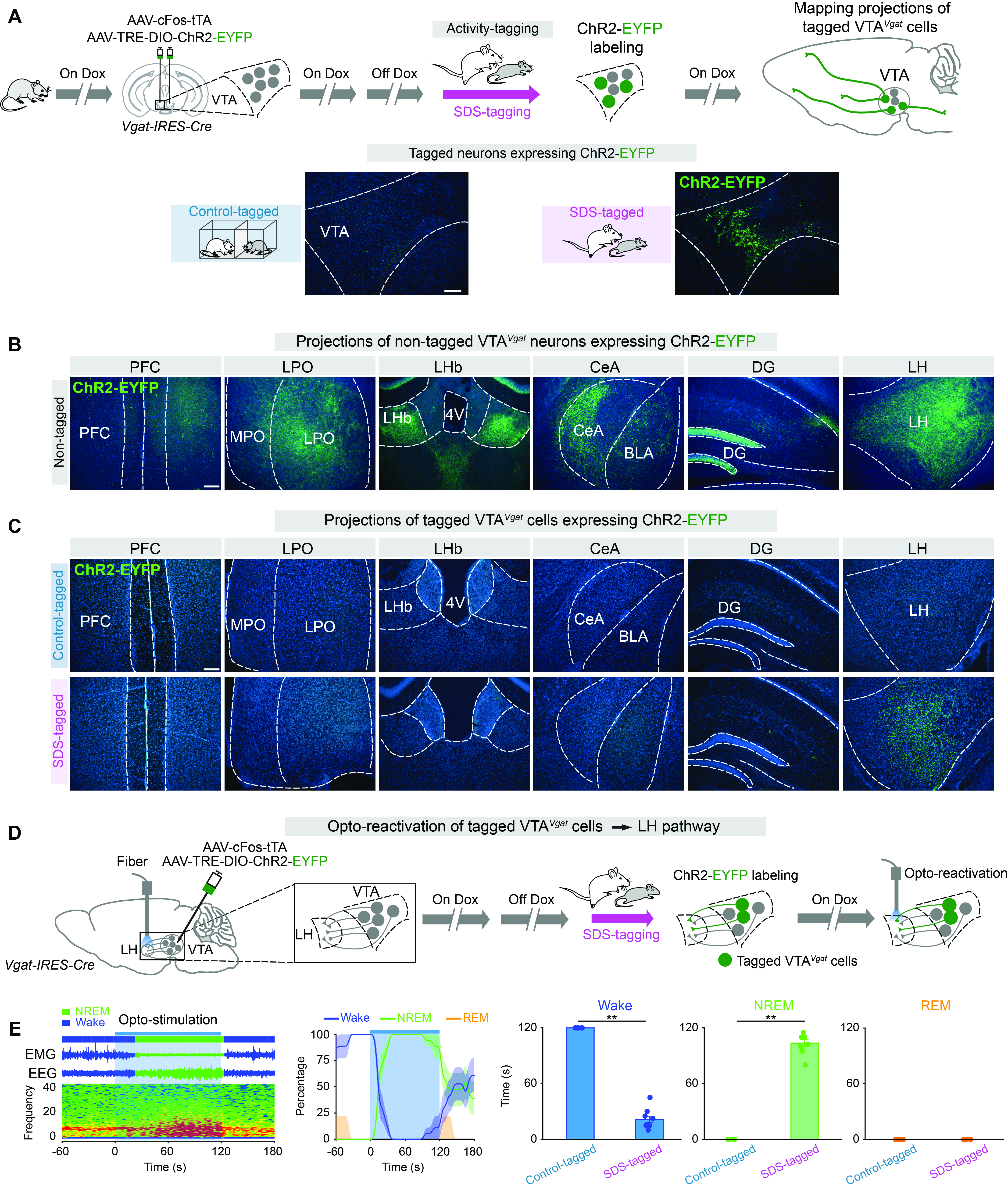

Supplement: Supp. Fig. S10 [file EMS145530-supplement-Supp__Fig__S10.jpg]

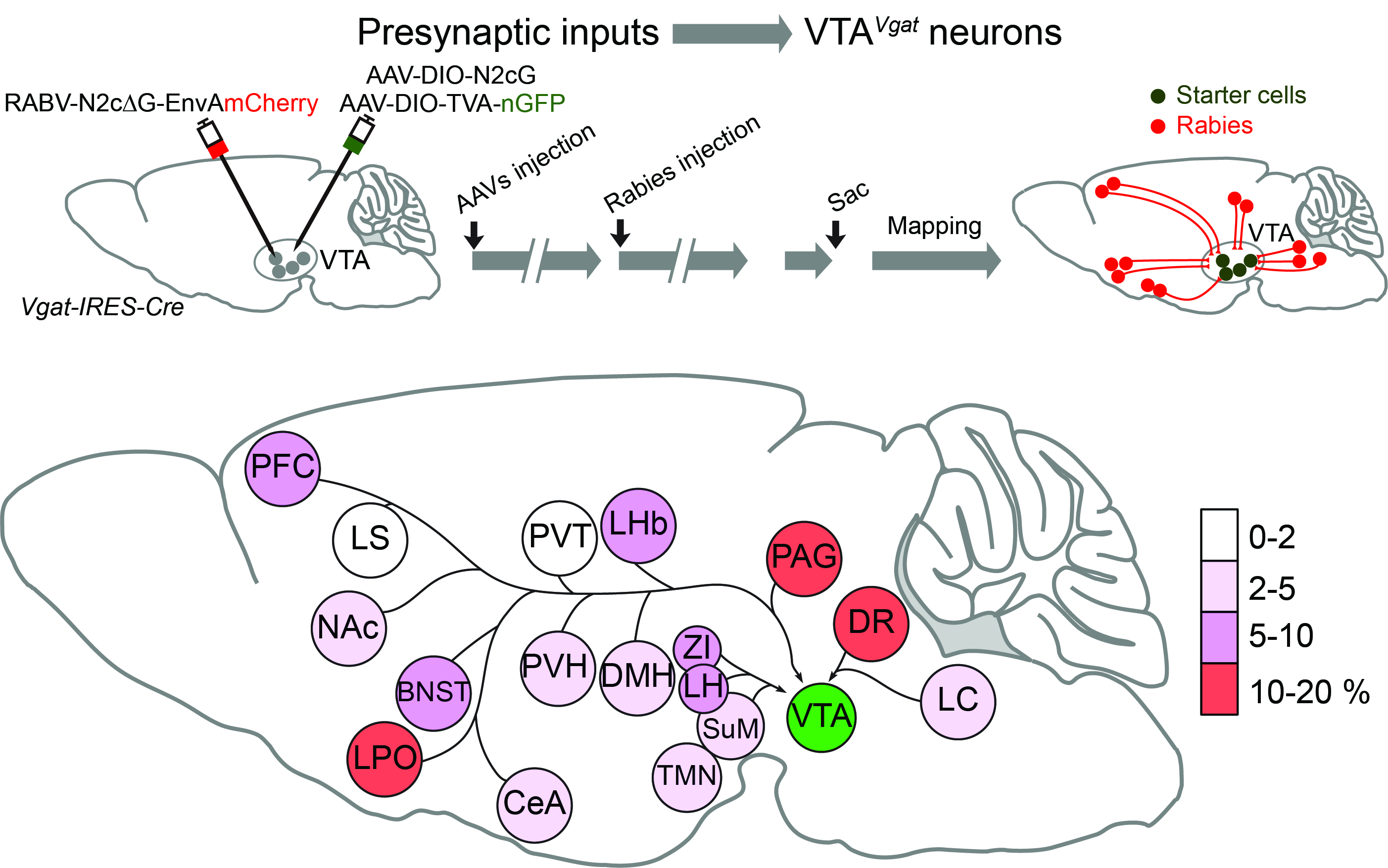

Supplement: Supp. Fig. S11 [file EMS145530-supplement-Supp__Fig__S11.jpg]

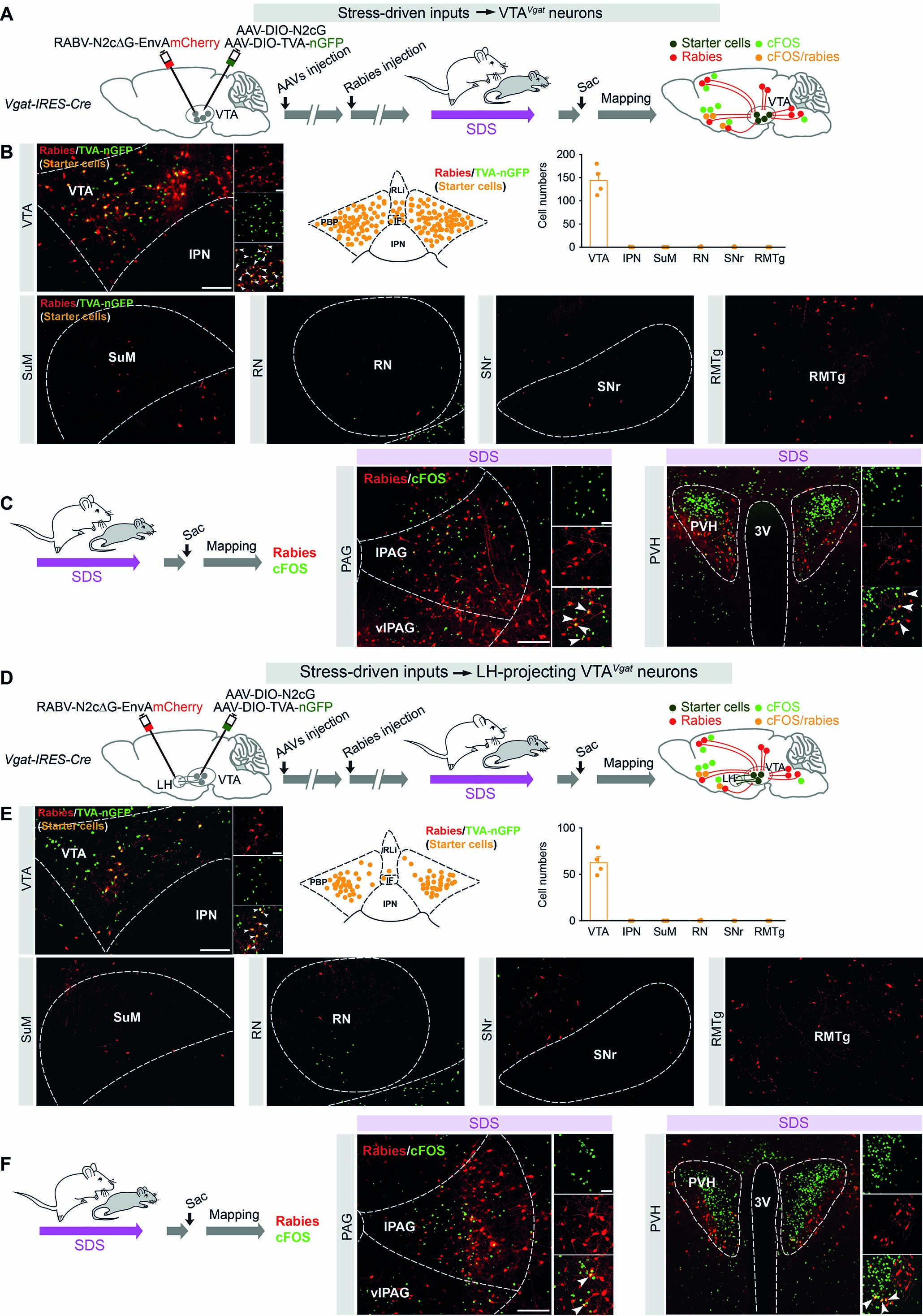

Supplement: Supp. Fig. S12 [file EMS145530-supplement-Supp__Fig__S12.jpg]

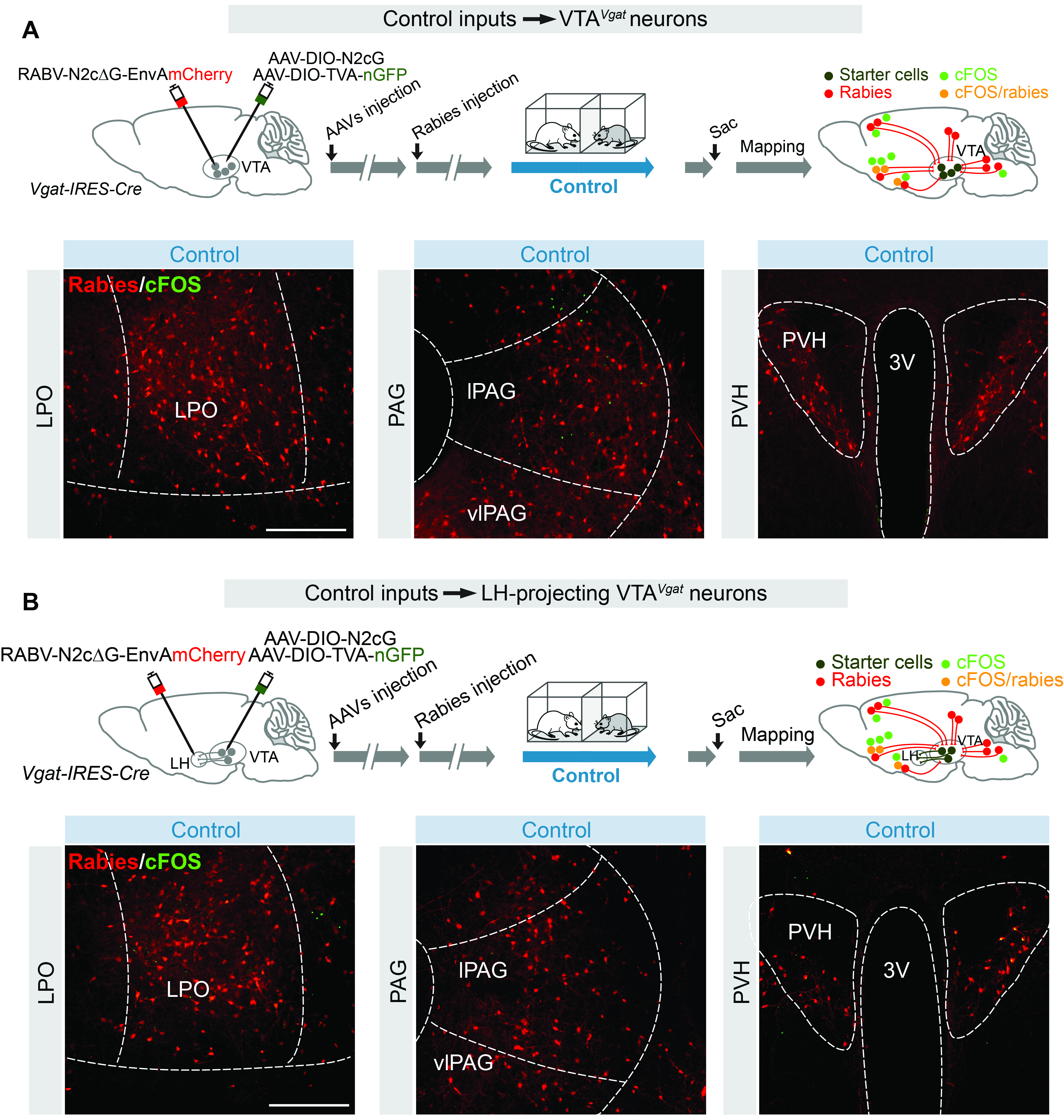

Supplement: Supp. Fig. S13 [file EMS145530-supplement-Supp__Fig__S13.jpg]

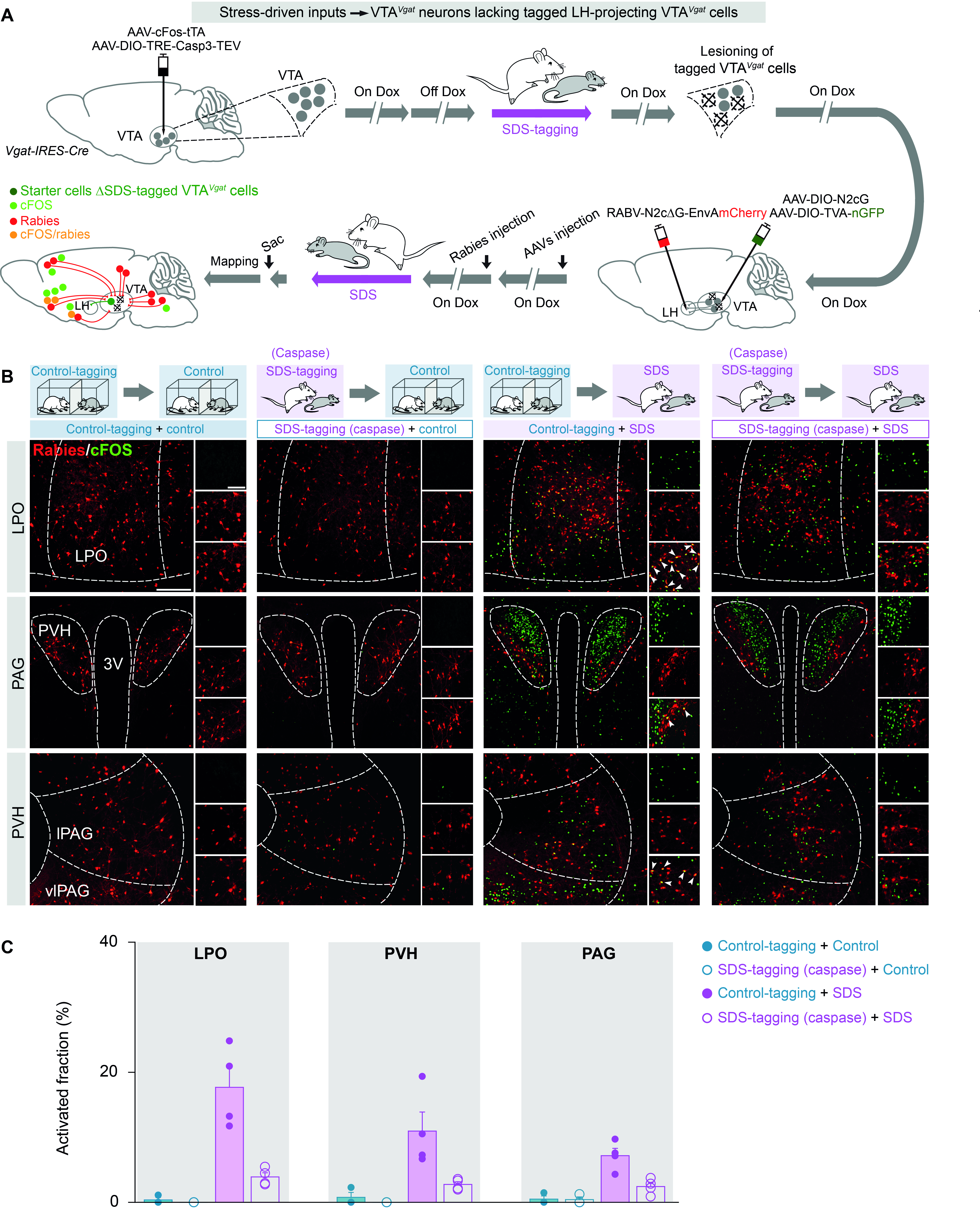

Supplement: Supp. Fig. S14 [file EMS145530-supplement-Supp__Fig__S14.jpg]

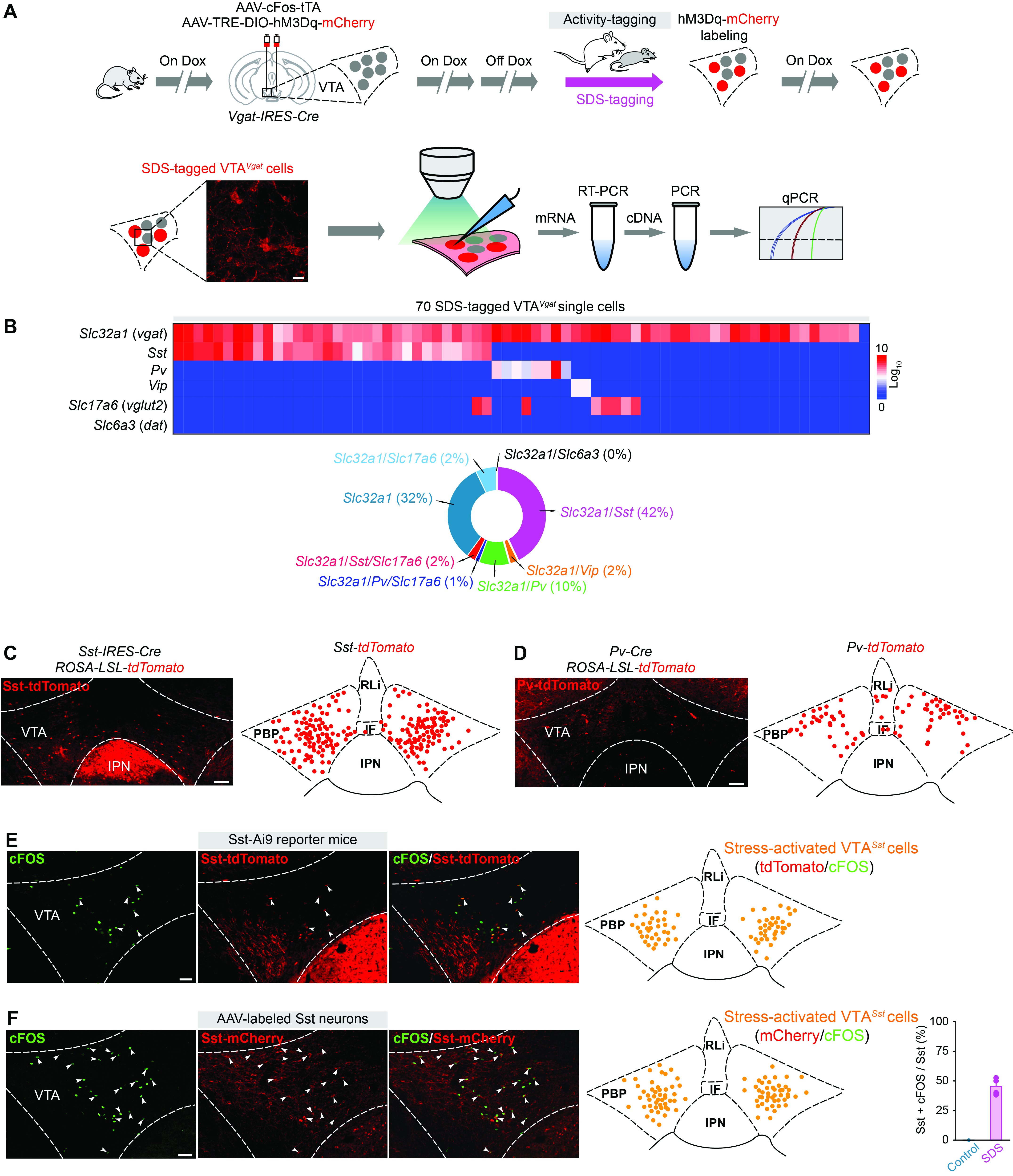

Supplement: Supp. Fig. S15 [file EMS145530-supplement-Supp__Fig__S15.jpg]

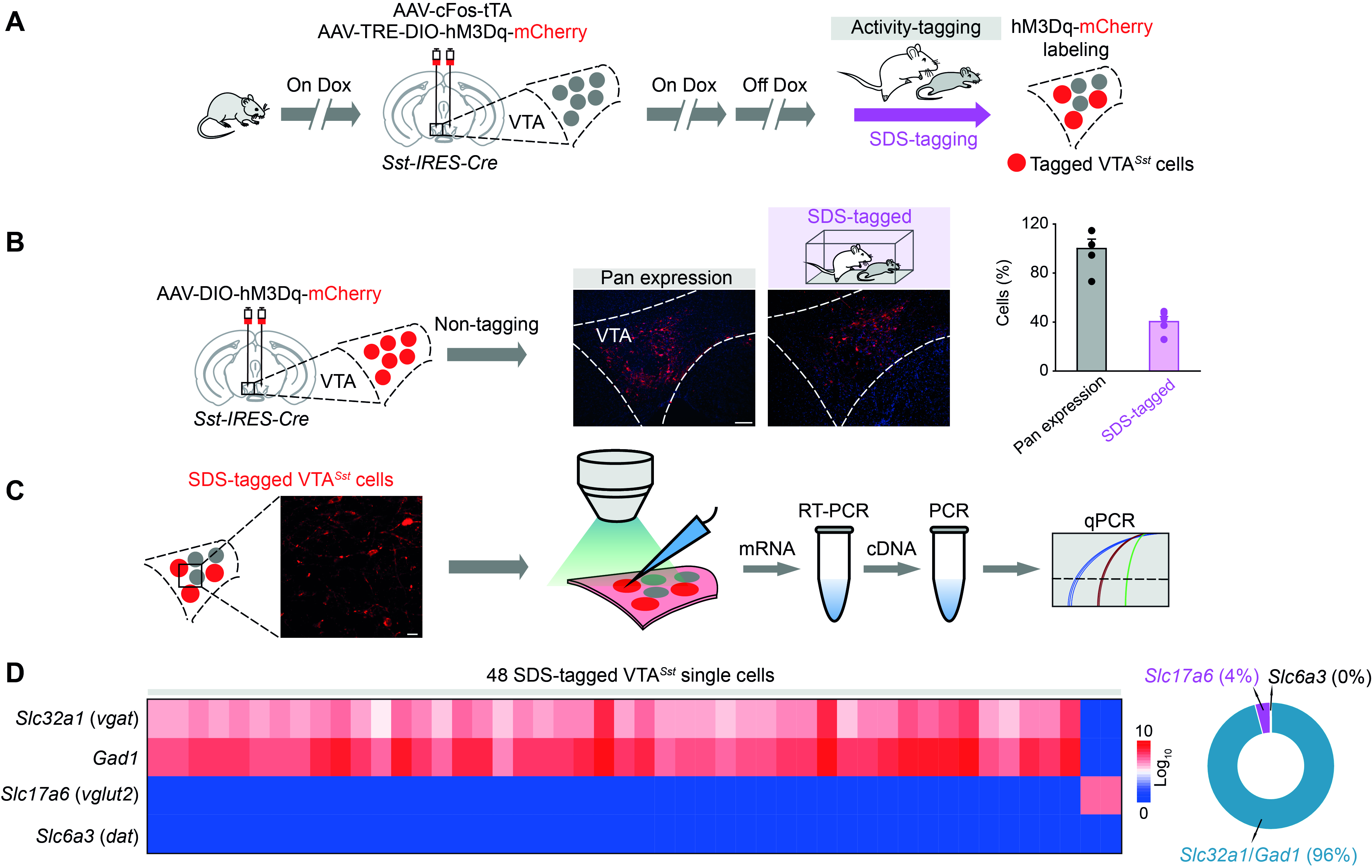

Supplement: Supp. Fig. S16 [file EMS145530-supplement-Supp__Fig__S16.jpg]

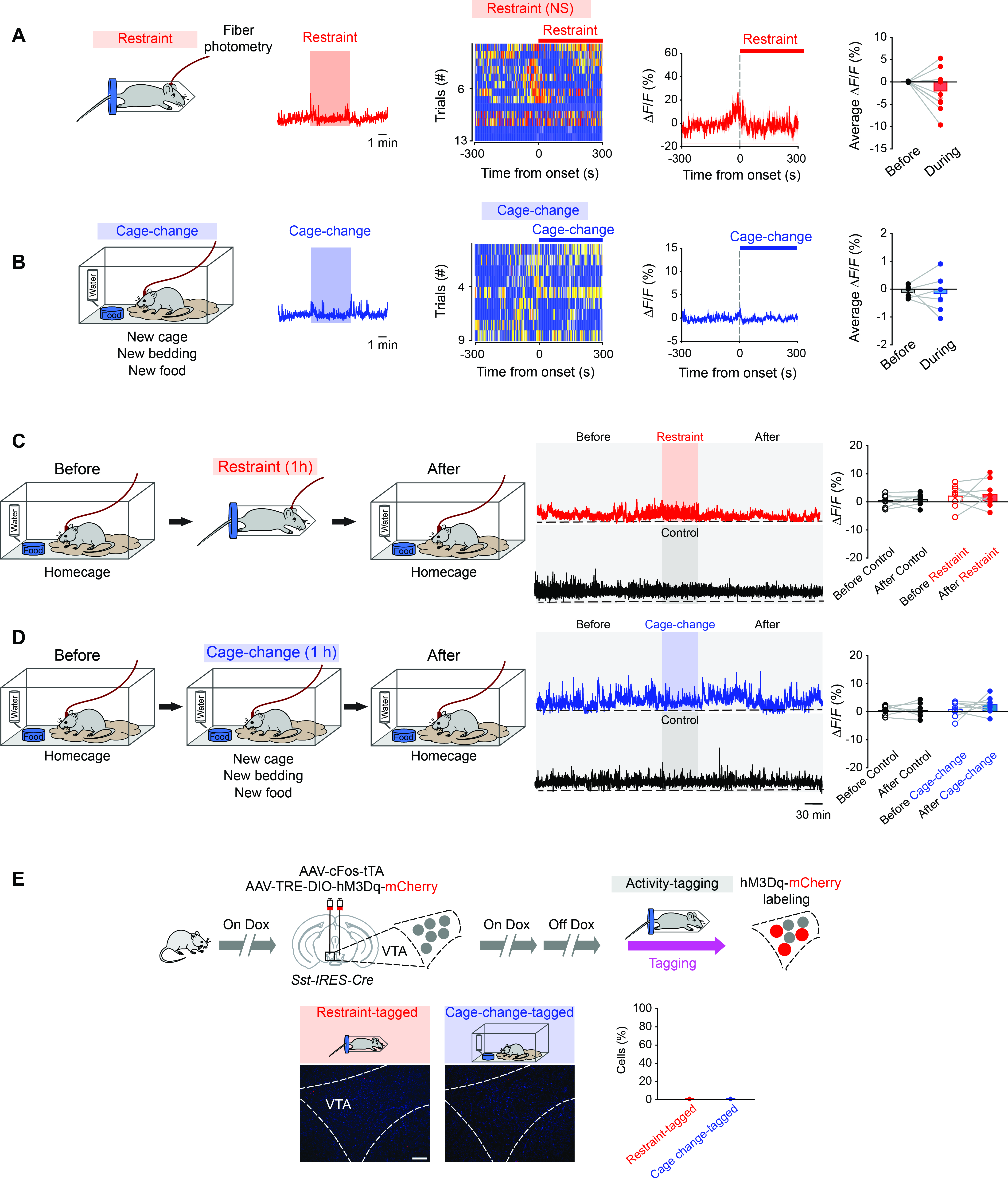

Supplement: Supp. Fig. S17 [file EMS145530-supplement-Supp__Fig__S17.jpg]

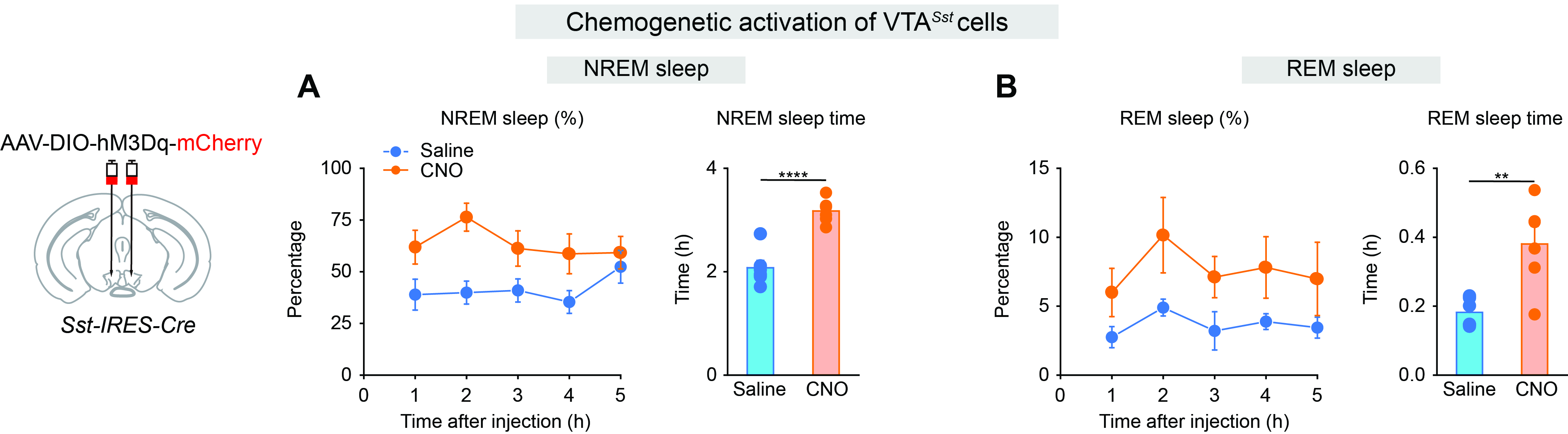

Supplement: Supp. Fig. S18 [file EMS145530-supplement-Supp__Fig__S18.jpg]

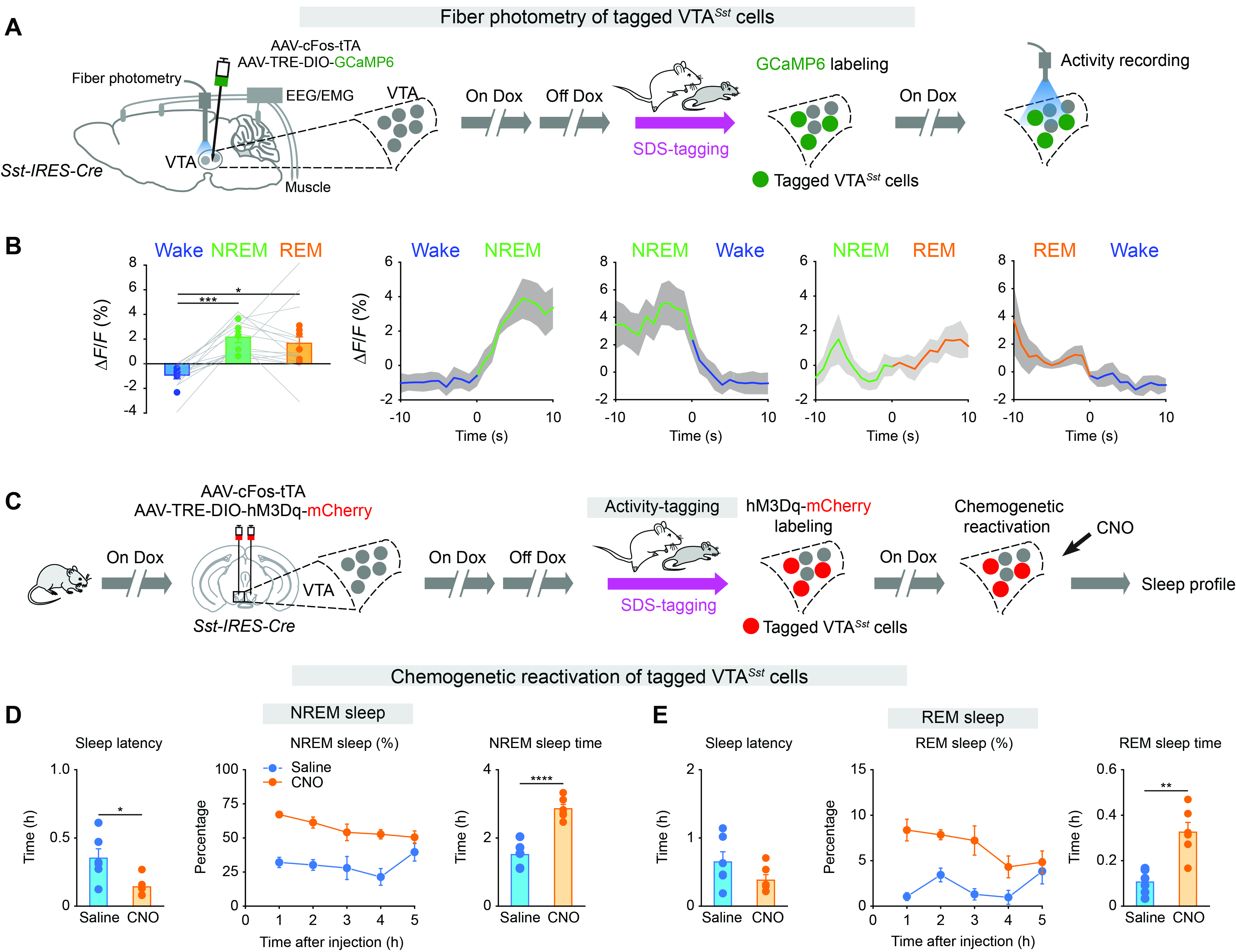

Supplement: Supp. Fig. S19 [file EMS145530-supplement-Supp__Fig__S19.jpg]

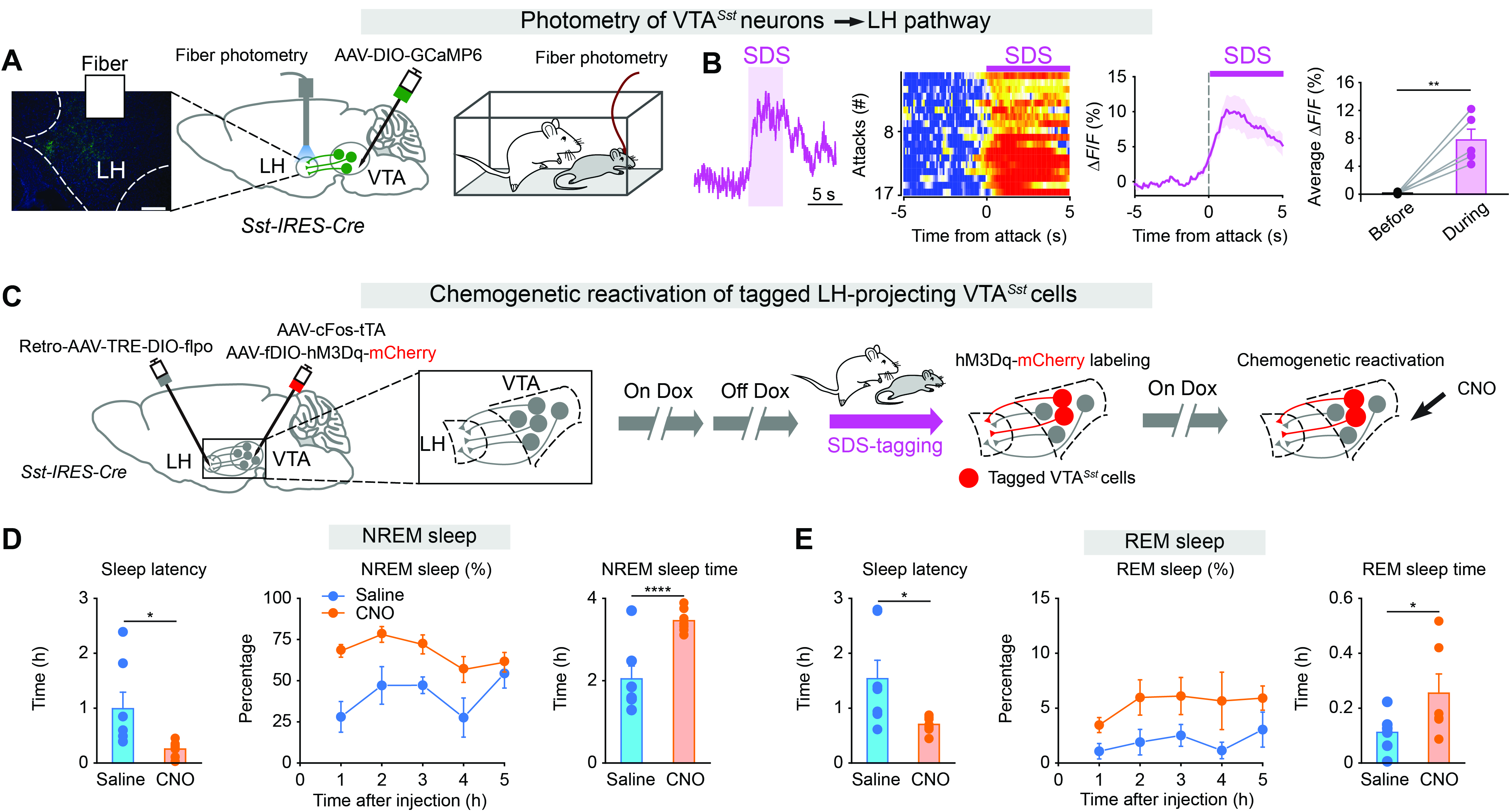

Supplement: Supp. Fig. S20 [file EMS145530-supplement-Supp__Fig__S20.jpg]

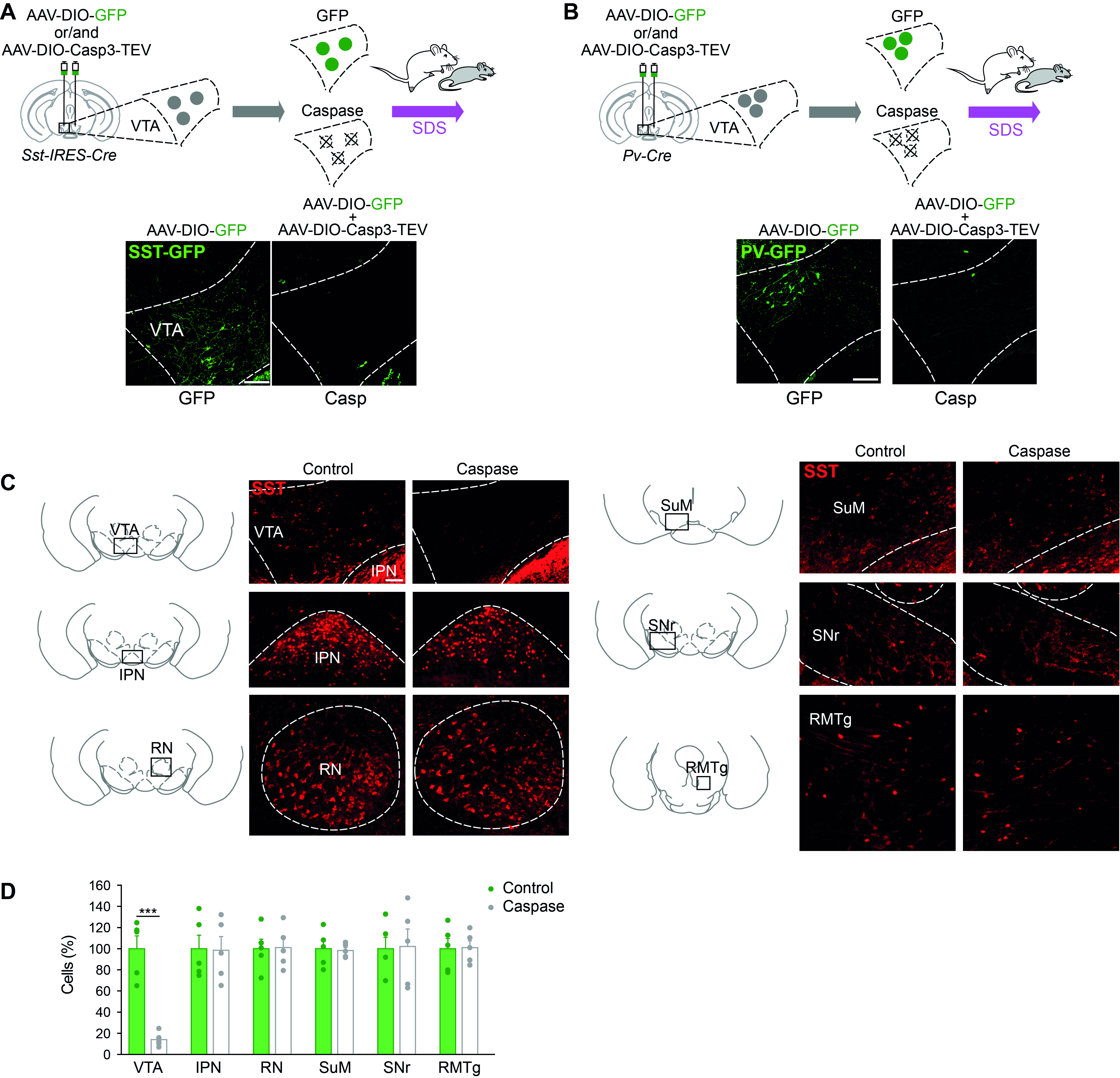

Supplement: Supp. Fig. S21 [file EMS145530-supplement-Supp__Fig__S21.jpg]

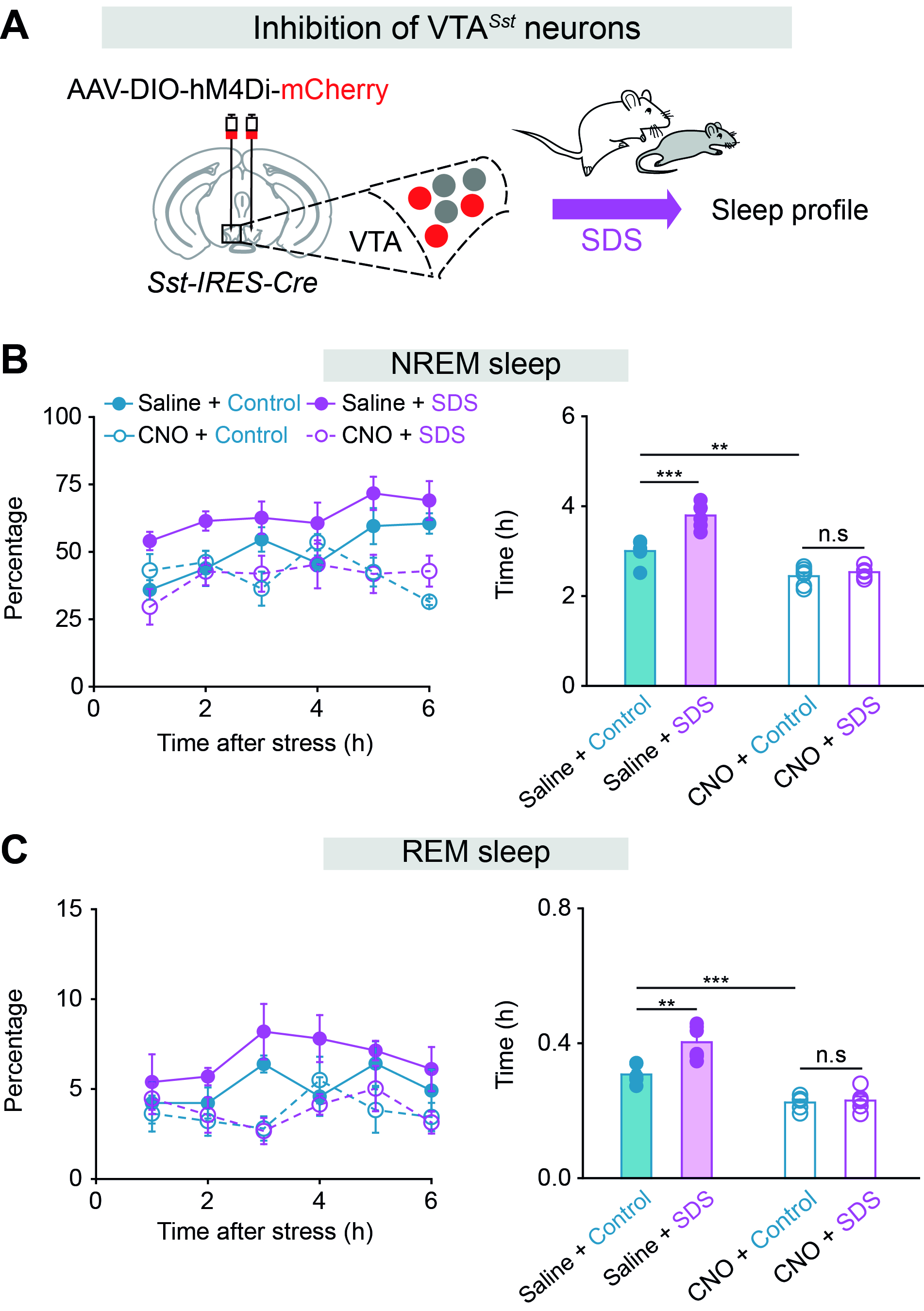

Supplement: Supp. Fig. S22 [file EMS145530-supplement-Supp__Fig__S22.jpg]

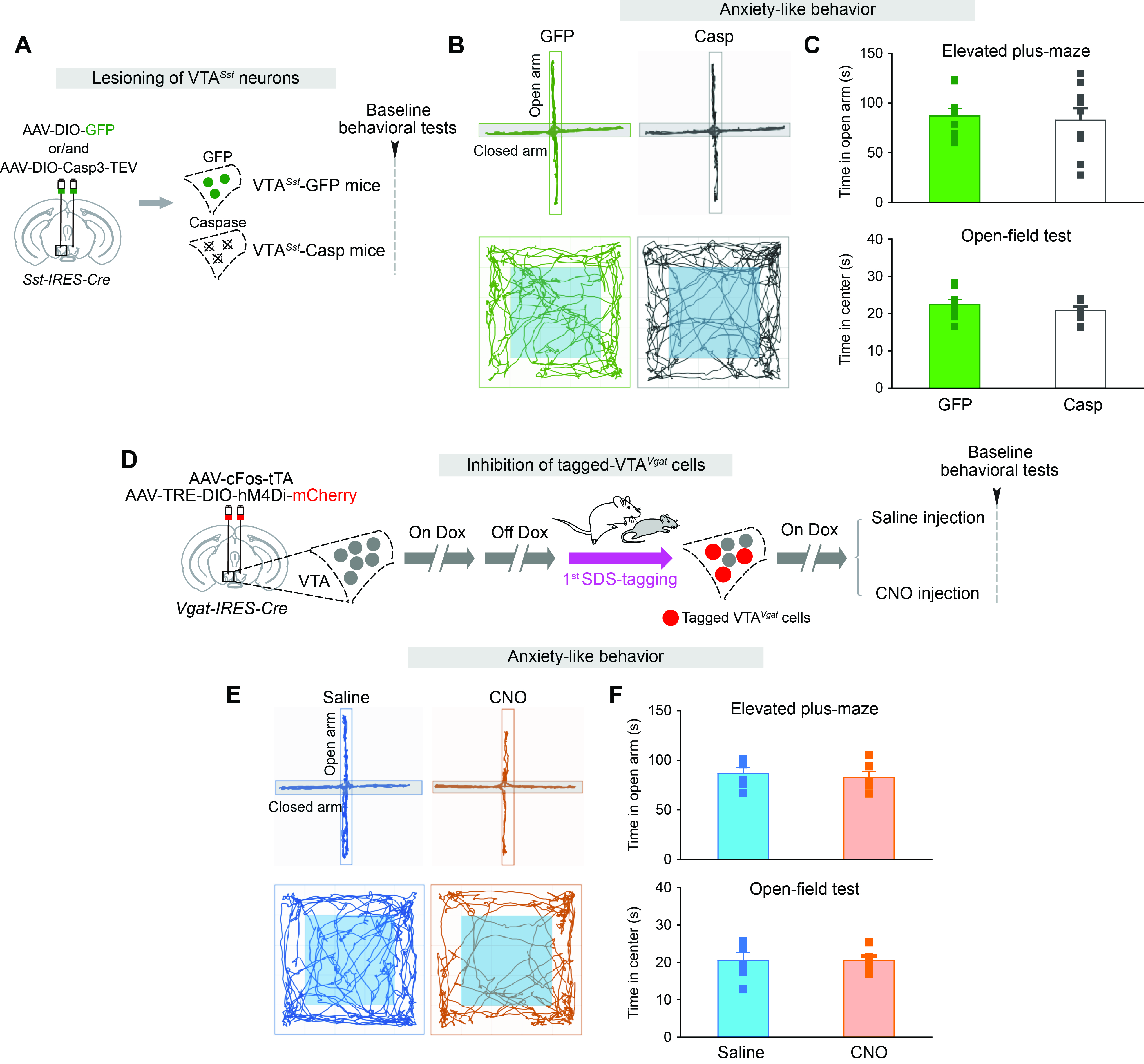

Supplement: Supp. Fig. S23 [file EMS145530-supplement-Supp__Fig__S23.jpg]

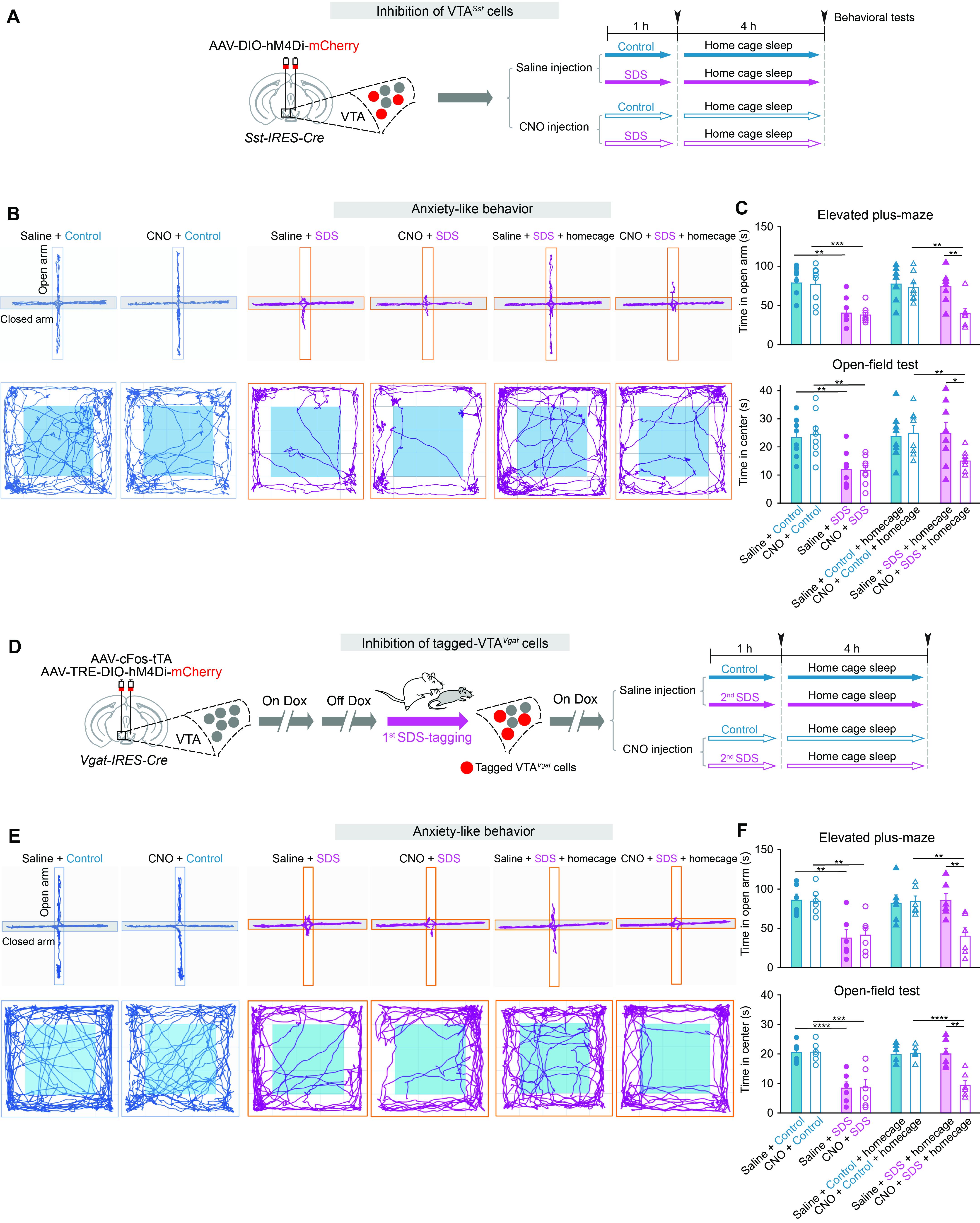

Supplement: Supp. Fig. S24 [file EMS145530-supplement-Supp__Fig__S24.jpg]

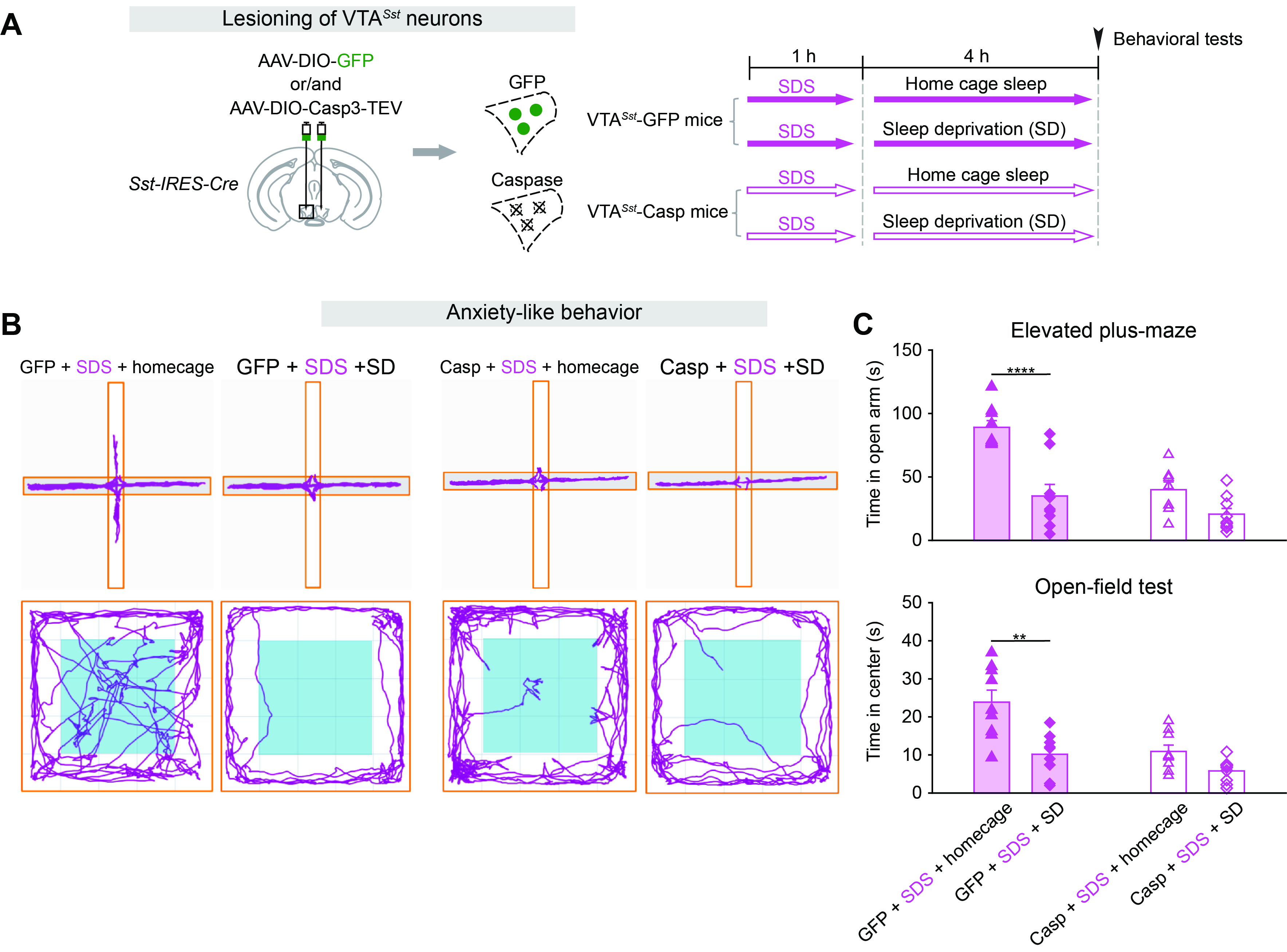

Supplement: Supp. Fig. S25 [file EMS145530-supplement-Supp__Fig__S25.jpg]

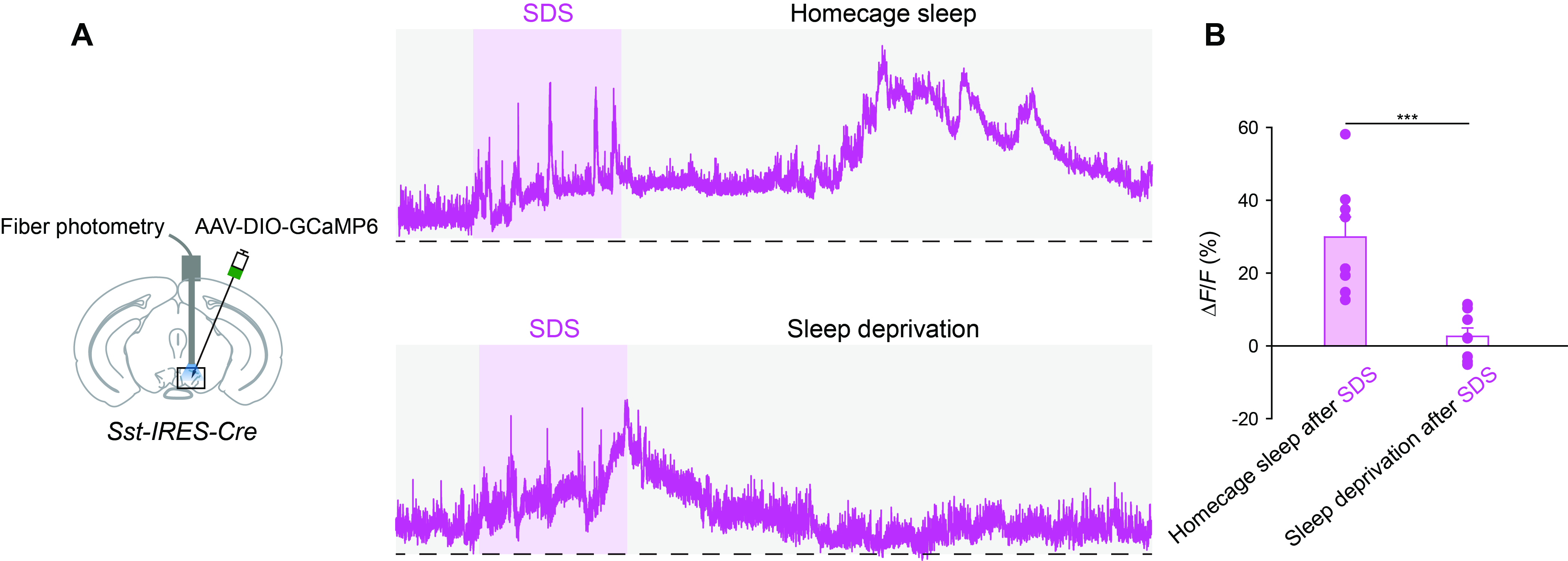

Supplement: Supp. Fig. S26 [file EMS145530-supplement-Supp__Fig__S26.jpg]

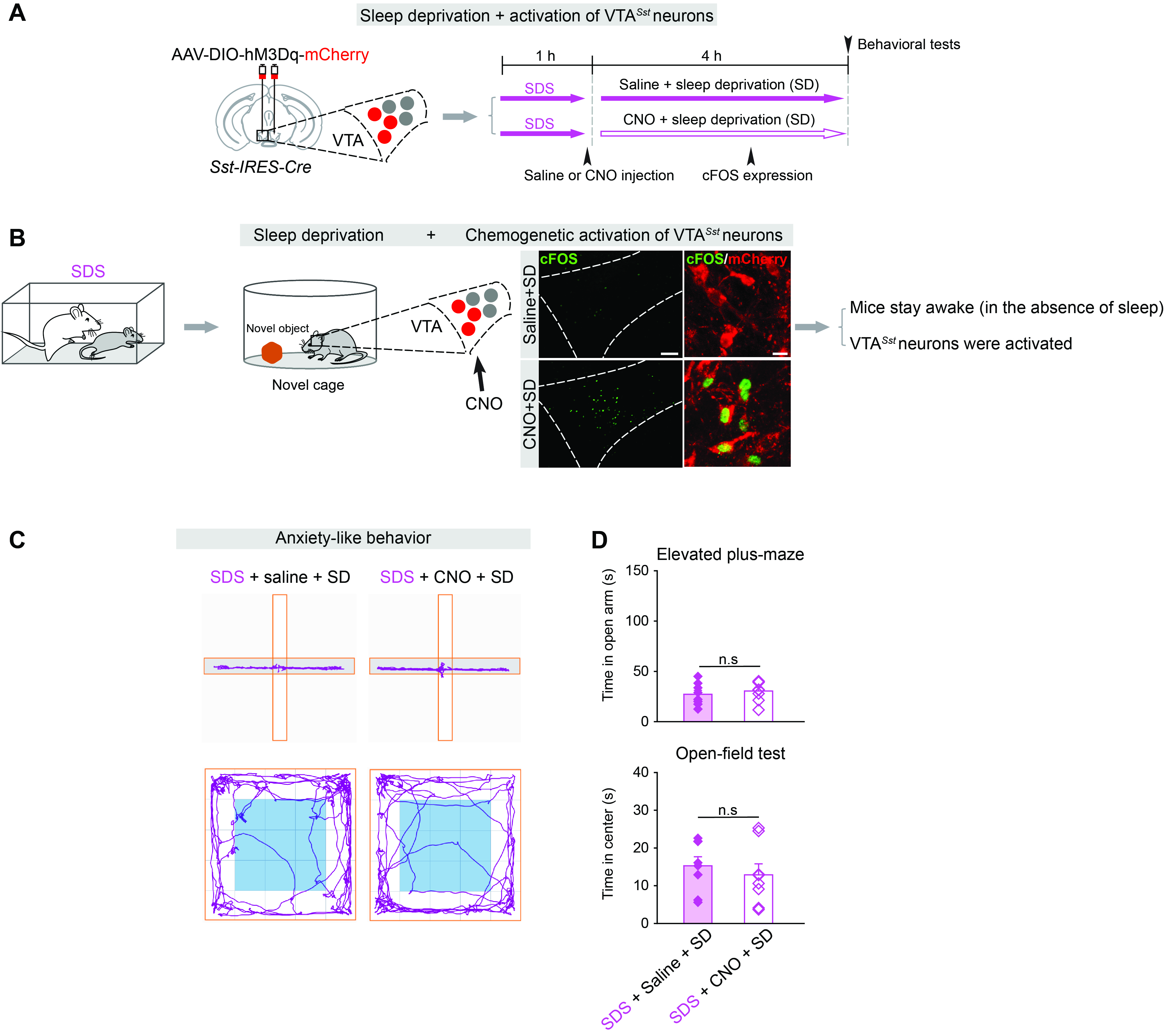

Supplement: Supp. Fig. S27 [file EMS145530-supplement-Supp__Fig__S27.jpg]

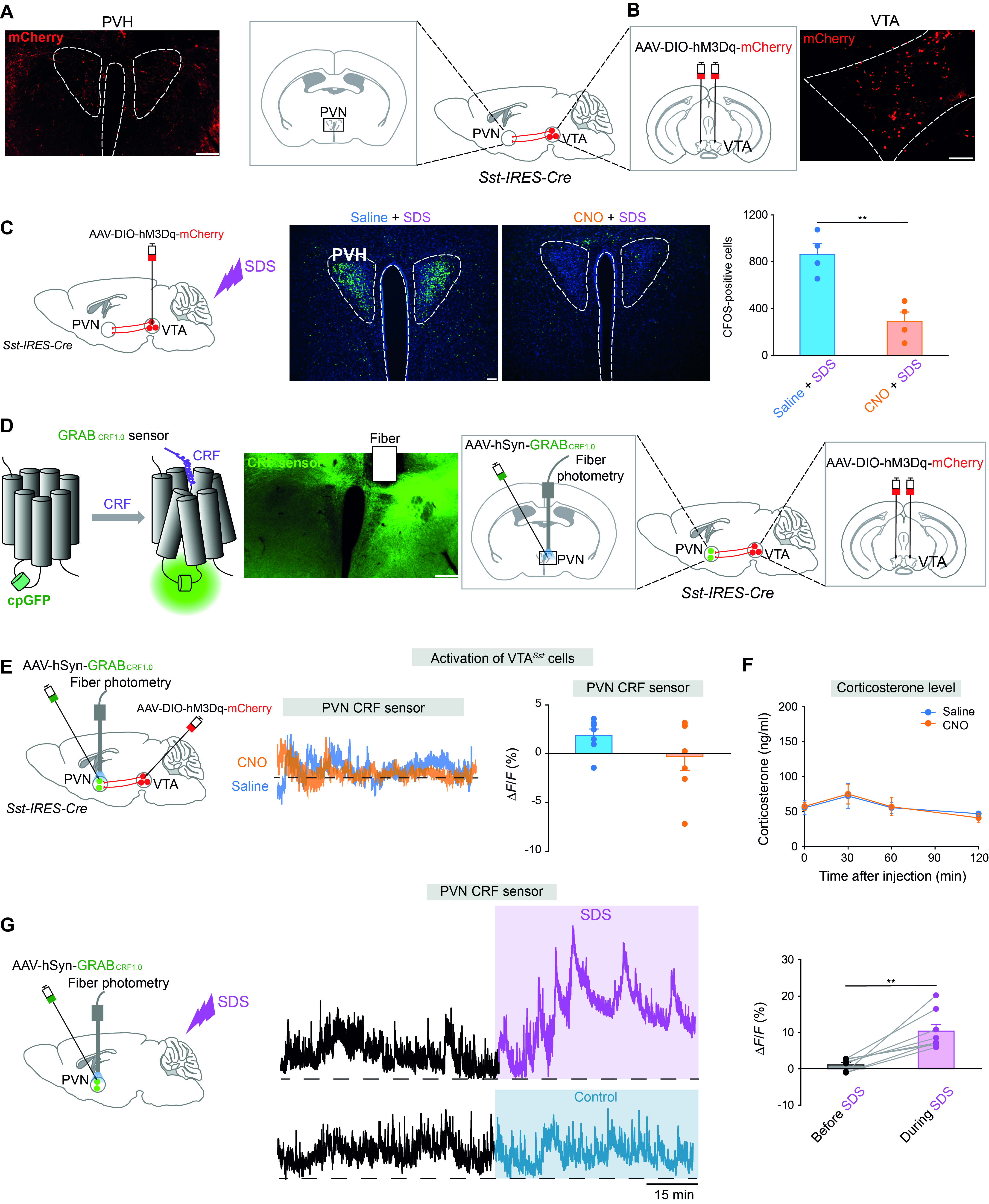

Supplement: Supp. Fig. S28 [file EMS145530-supplement-Supp__Fig__S28.jpg]

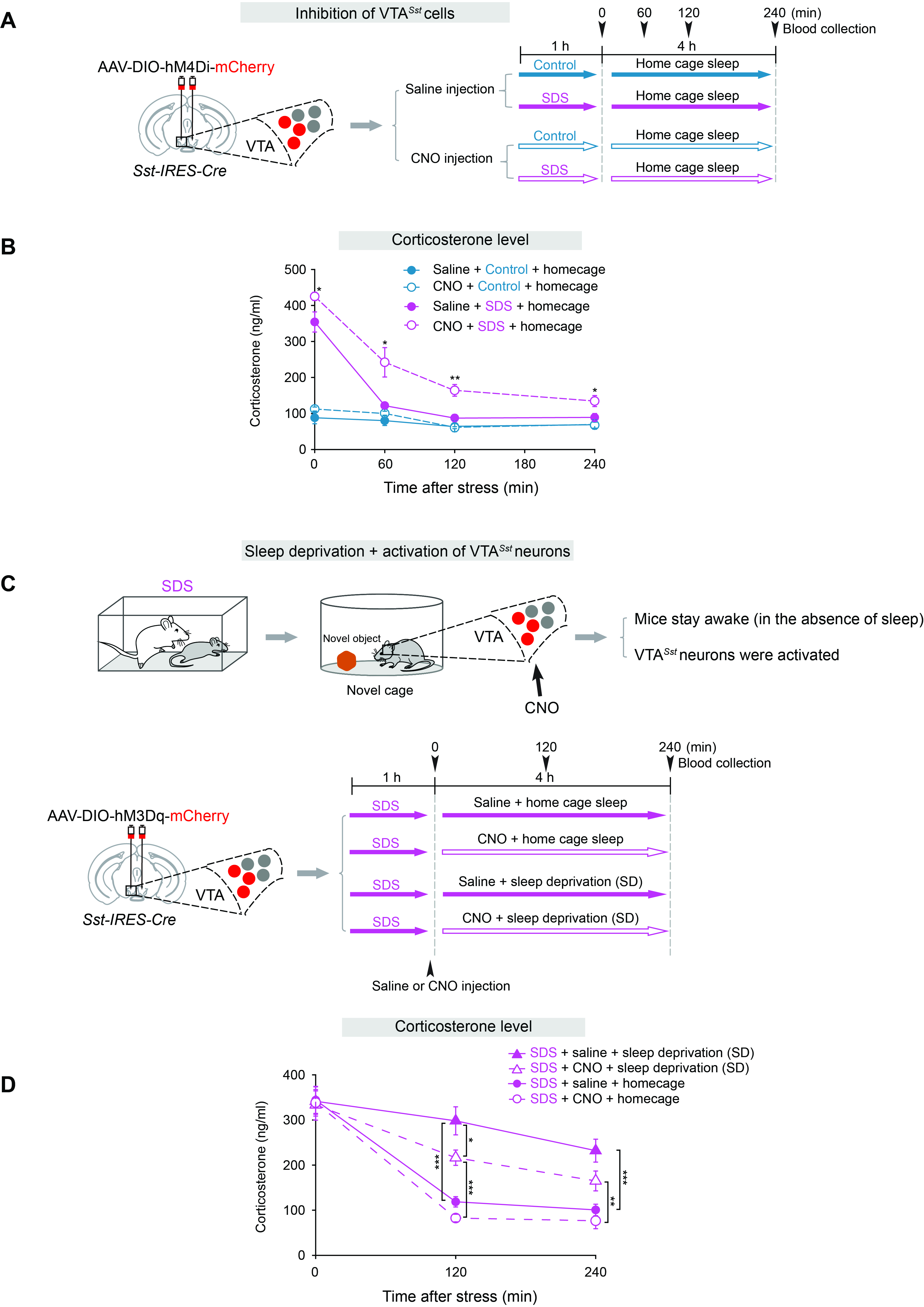

Supplement: Supp. Fig. S29 [file EMS145530-supplement-Supp__Fig__S29.jpg]

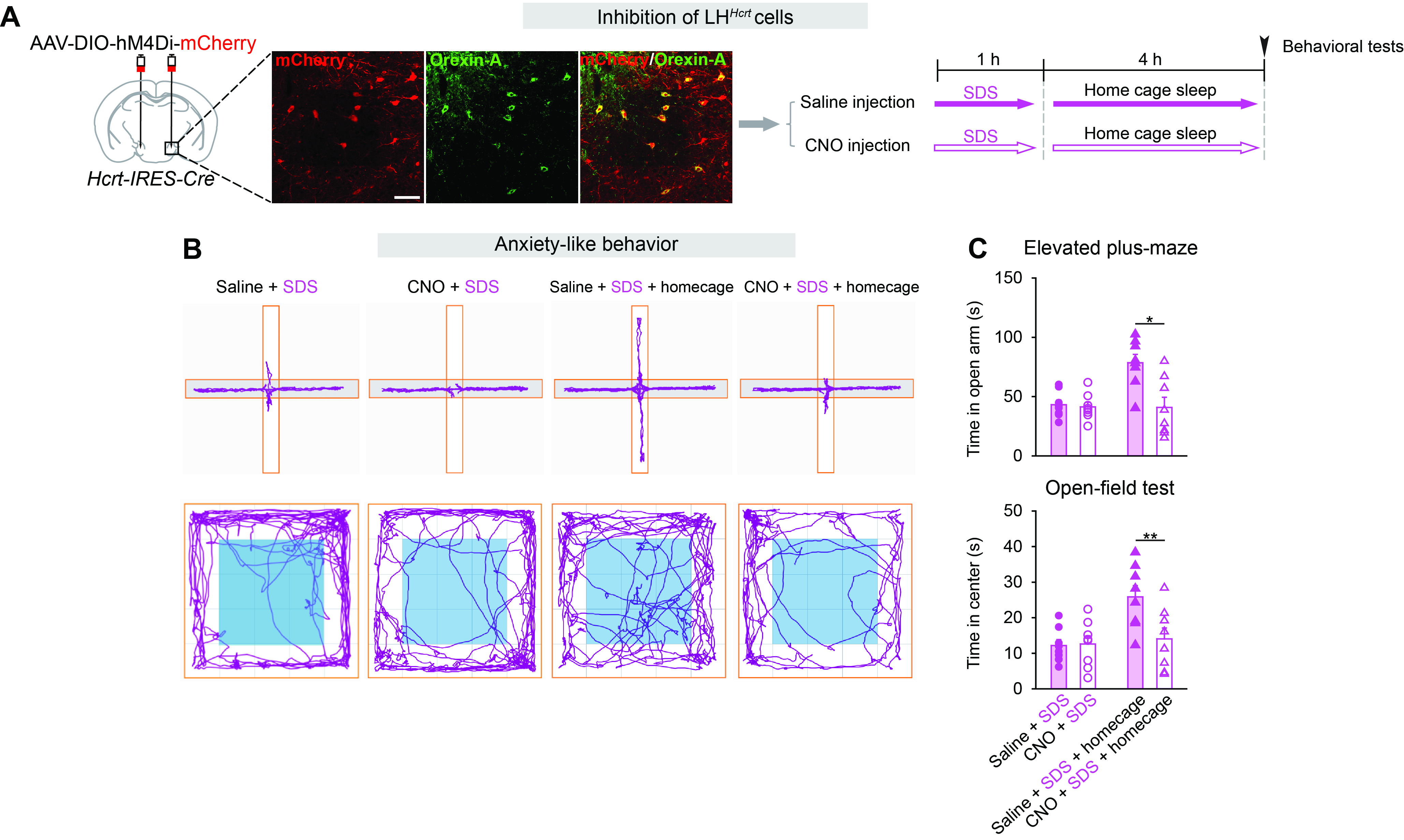

Supplement: Supp. Fig. S30 [file EMS145530-supplement-Supp__Fig__S30.jpg]

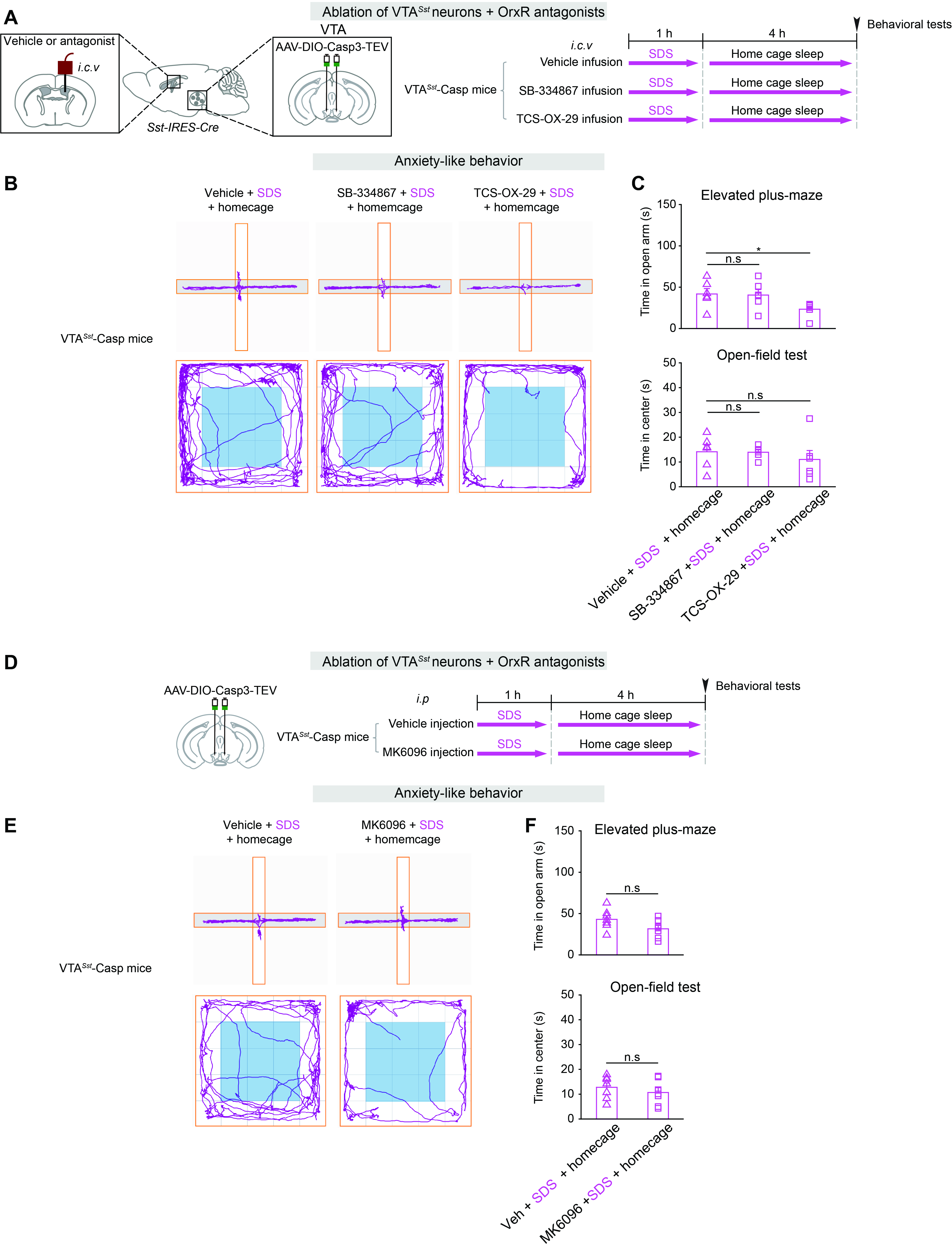

Supplement: Supp. Fig. S31 [file EMS145530-supplement-Supp__Fig__S31.jpg]

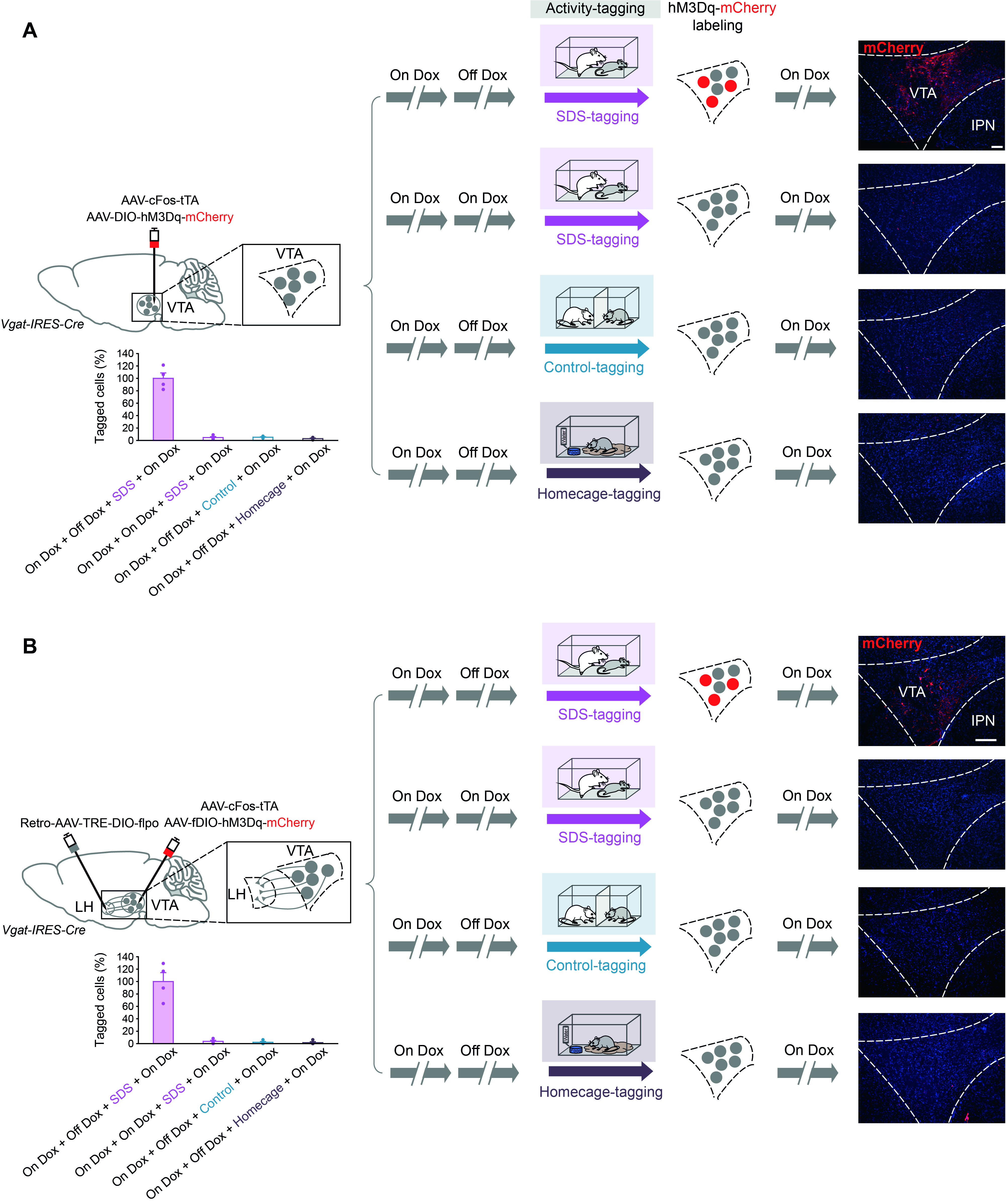

Supplement: Supp. Fig. S32 [file EMS145530-supplement-Supp__Fig__S32.jpg]

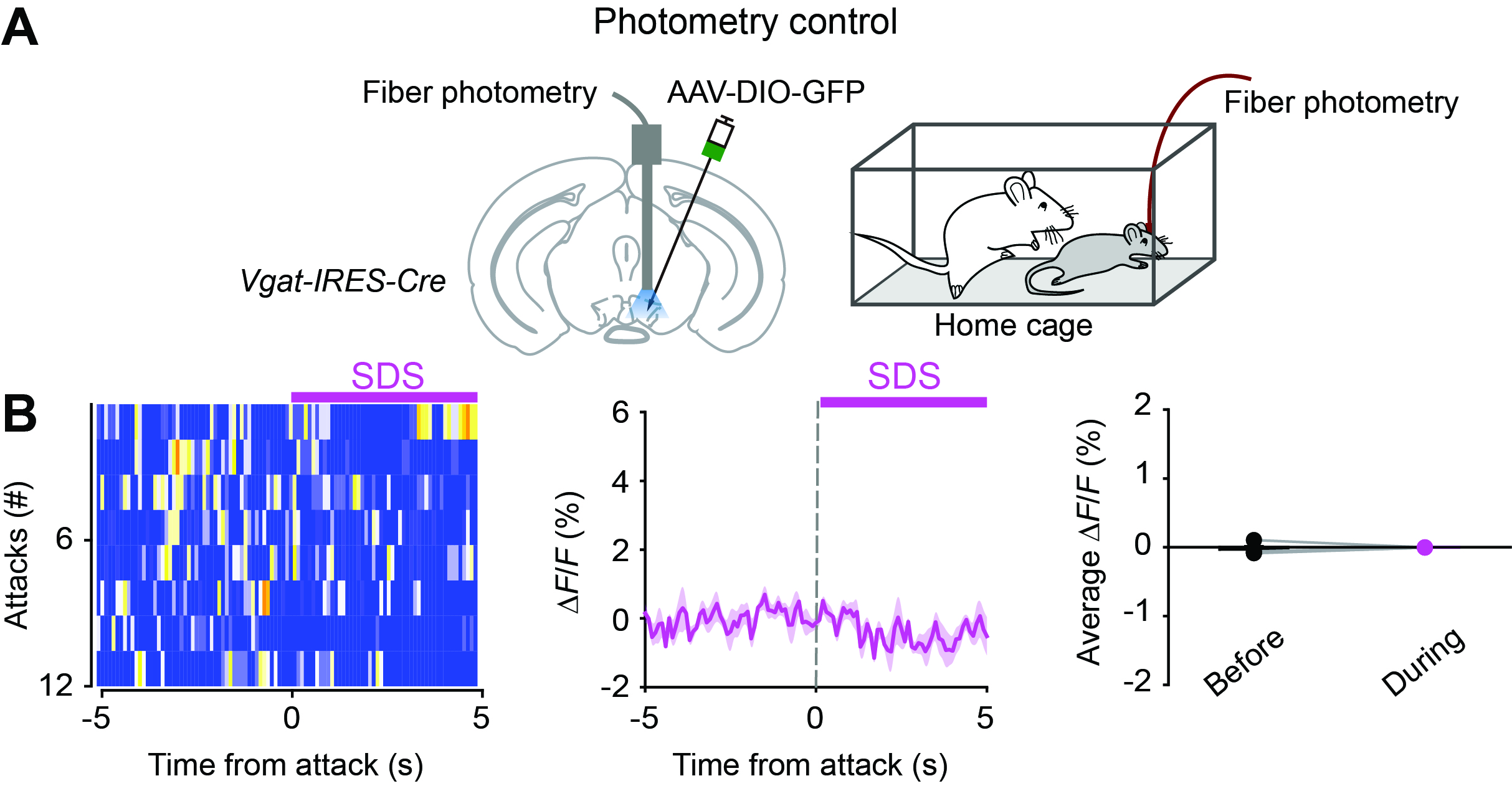

Supplement: Supp. Fig. S33 [file EMS145530-supplement-Supp__Fig__S33.jpg]
